# Dicer is essential for proper maturation, composition, and function in the postnatal retina

**DOI:** 10.1016/j.isci.2025.113794

**Published:** 2025-10-17

**Authors:** Seoyoung Kang, Daniel Larbi, Eik Bruns, Konstantin Hahne, Alireza Khodadadi-Jamayran, Chaitra Sreenivasaiah, Mariana Lima Carneiro, Monica Andrade, Khulan Batsuuri, Shaoheng Chen, Julia Jager, Suresh Viswanathan, Brian Stewart Clark, Stefanie Gabriele Wohl

**Affiliations:** 1 Department of Biological and Vision Sciences, The State University of New York, College of Optometry, New York, NY, USA; 2 Applied Bioinformatics Laboratories, Office of Science and Research, New York University School of Medicine, New York, NY, USA; 3 Indiana University School of Optometry, Bloomington, IN, USA; 4 John F Hardesty, MD Department of Ophthalmology and Visual Sciences and Department of Developmental Biology, Washington University School of Medicine, St. Louis, MO, USA

**Keywords:** Molecular biology, Neuroscience, cell biology, developmental biology

## Abstract

The impact of Dicer/miRNAs during postnatal retinogenesis is unknown. This study aimed to deplete Dicer in postnatal retinal progenitor/precursor cells (RPCs/PCs) and to evaluate the structure and function of the adult Dicer-cKO retina using optical coherence tomography (OCT), electroretinography (ERG), and histology. We found impaired rod function, a significantly reduced number of rod bipolar cells and their associated functions, a decreased Müller glia population, and an increased population of HuC/D + Pax6/+ amacrine cells in the adult Dicer-cKO retina. Transcriptional analyses conducted in one-week-old mice showed early alterations in mRNA and miRNA levels. Single-cell RNA sequencing and initial histological analysis in young mice revealed a delay in differentiation/incomplete maturation, as indicated by an enlarged progenitor/precursor population at young ages that persists into adulthood. This suggests that Dicer/miRNAs in late RPC/PCs are essential for the proper formation and maturation of late RPC progenies and may also play a role in regulating cell state.

## Highlights

• Postnatal progenitor Dicer/miRNAs are essential for retinal cell maturation
• Postnatal Dicer loss impacts rod, bipolar cell, and Müller glia development
• Postnatal Dicer loss in retinal progenitors leads to amacrine cell overproduction
• Dicer loss causes an immature cell population to persist until adulthood

## Graphical abstract

**Figure:**
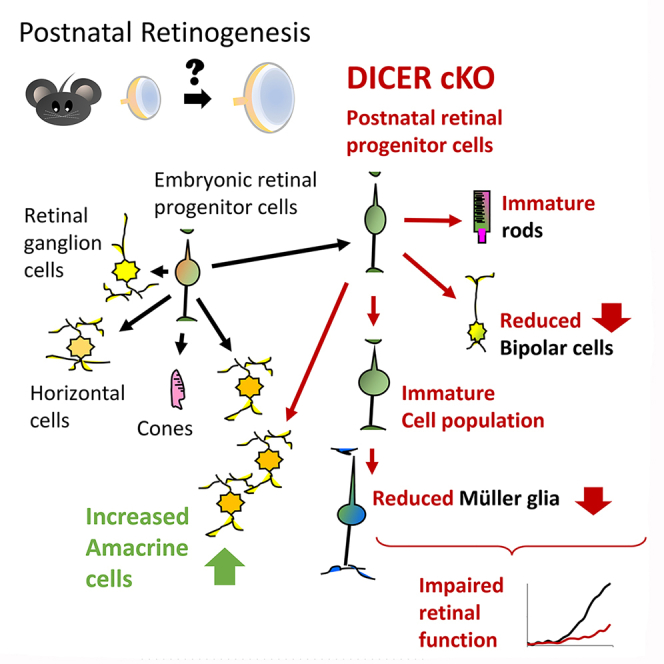

## Introduction

Retinogenesis is a complex and highly regulated process across all vertebrates. There are two principal phases in which all six retinal neuron types are generated: First, retinal ganglion cells (RGCs), cone photoreceptors, horizontal cells (HCs), and early-born amacrine cell types (ACs) arise from early (embryonic) retinal progenitor cells (eRPCs); second: rod photoreceptors, bipolar cells (BCs), late-born ACs, and Müller glia (MG), the only non-neuronal cell type, are generated from late, postmitotic (p) RPCs.^1^^,^^2^^,^^3^^,^^4^^,^^5^^,^^6^^,^^7^^,^^8^^,^^9^^,^^10^^,^^11^^,^^12^^,^^13^ All retinal cell types are found in designated retinal layers, forming distinct connections to neighboring cells, and express their cell-type-specific markers by postnatal day (P) 12. Full tissue maturation is, however, not completed until P28 (full visual acuity).^14^

Various molecules, primarily specific combinations of transcription factors, have been identified in the past decades that play a role in neuro- and gliogenesis in the nervous system, including the neural retina. Dicer/microRNAs (miRNAs) are known to regulate cell proliferation, cell fate decisions, cell differentiation, and maturation in various tissues, including the brain and the neural retina^15^^,^^16^^,^^17^^,^^18^^,^^19^^,^^20^^,^^21^^,^^22^^,^^23^^,^^24^^,^^25^^,^^26^^,^^27^^,^^28^^,^^29^*.* These short (18–22 nucleotides long) non-coding RNAs function as translational repressors.^30^^,^^31^^,^^32^^,^^33^ They are encoded in the exons or introns of genes and processed by the ribonuclease III (RNase III) enzyme complexes, known as Drosha- and Dicer-complexes. The Drosha-complex generates a precursor molecule from the primary transcript, while the Dicer-complex processes the precursor molecule into the mature functional miRNA.^31^^,^^32^^,^^33^ A very efficient method to study the role of tissue- or cell-type-specific miRNAs is therefore via loss-of-function experiments by conditionally deleting the enzyme (or its function) that generates mature miRNAs, Dicer. To our knowledge, Dicer-conditional knock-out (cKO) studies have only been carried out exclusively during embryonic retinal development, resulting in mostly severe outcomes, including microphthalmia, rosette formation, and massive cell loss, leading to severe functional defects.^34^^,^^35^^,^^36^^,^^37^ Intriguingly, the loss of Dicer/mature miRNAs in eRPCs (E12/14) led to an overproduction of early-born cells (RGCs, HCs, ACs) and a failure to produce late-born cells (rods, BCs, MG), as shown by two independent laboratories.^38^^,^^39^ However, most of these studies were performed over a decade ago. Many techniques have advanced over the years, now allowing for defined manipulation and evaluation at the single-cell level. Furthermore, to date, no data are available regarding the role of Dicer and miRNAs in postnatal RPCs/PCs in terms of the generation, maturation, and functionality of their progeny cells, specifically rods, BCs, late-born ACs, and MG.

This study aimed to analyze the impact of Dicer/miRNA loss in late (postnatal) RPCs/PCs on the composition, function, and overall health of the adult retina. We utilized the late RPC-specific *Ascl1CreERT:tdTomato* reporter mouse.^13^^,^^40^^,^^41^ *Ascl1* (Achaete-Scute homolog 1, also known as *Mash1*), a pro-neural basic-helix-loop-helix [bHLH] transcription factor, is expressed in late RPCs/PCs that generate the late-born retinal cells. It is not expressed in differentiated neurons or glia.^13^^,^^42^ This reporter mouse was crossed with the Dicer^f/f^ mouse.^43^ Reporter expression/Dicer loss was induced at postnatal days (P) 1–3, i.e., the peak of the second phase of retinogenesis.^9^^,^^44^ *In vivo* analyses using spectral-domain optical coherence tomography (SD-OCT) and electroretinography (ERG) in combination with histology were conducted predominantly at P56, as well as at P28, 3 months, and 4 months. Molecular analyses to confirm genetic alterations and initial histological instigations were performed at P7 and P14.

Our data show that loss of Dicer/mature miRNAs in postnatal RPCs/PCs results in prolonged cell division events and a delay of maturation in the young postnatal retina. This delay leads to functional impairments and a reduction of the overall number of late RPC/PC progenies in the adult retina, predominantly in the nasal periphery. The most affected were central rod BCs and peripheral MG. Overall, the process of retinogenesis appeared incomplete, resulting in a residual immature population in the adult retina. The failure to mature correctly results in functional impairments. Subsequent retinal degeneration becomes evident as early as two months after Dicer deletion, but progresses very slowly. Moreover, Dicer-cKO_RPC_ retinas have an increased HuC/D+ AC population. We hypothesized that increased *Elavl3* expression, an upregulated RPC-miRNA target and the gene encoding HuC protein, might cause this. Overall, postnatal RPC-miRNAs appear to be essential for the proper maturation of late-born retinal populations.

## Results

### Dicer loss in late RPCs/precursors results in delays in maturation

**Figure 1:**
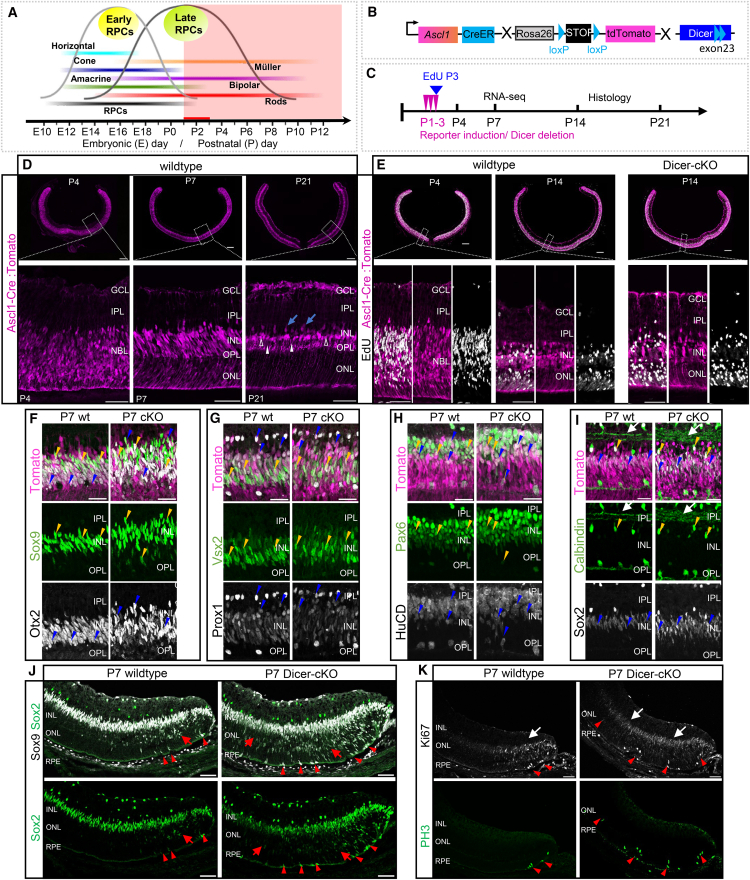
Dicer-cKO retinas have an enlarged progenitor/precursor cell population (A) Mouse retinogenesis schematic with time point of manipulation (red line, after Jin and Xiang^2^). (B) Simplified schematics of retinal progenitor cell (RPC) reporter mouse (wild type, wt) and Dicer-cKO mouse. Note that only Exon 23 of the Dicer1 gene is floxed. (C) Experimental design with time points of *Cre* induction and EdU labeling. (D and E) P4, P7, P14, and P21 wild type and cKO retinal cross sections with magnified insets visualizing endogenous reporter expression of RPC progenies labeled at P1-3, that underwent cell division at P3; blue arrows in D show presumptive amacrine cells, white arrowheads presumptive bipolar cells, unfilled arrowheads presumptive Müller glia; EdU+ progenies are shown in E (see also additional data Figure S1). (F–I) Labeling with antibodies against Sox9 and Otx2 (F), Vsx2 and Prox1 (G), Pax6 and HuC/D (H), and Calbindin and Sox2 (I) of the INL of P7 wild type or Dicer-cKO central retinal cross sections to characterize Tomato+ RPC progenies (arrowheads; see also additional data in Figure S2). Markers depicted in white are labeled in black font. Arrows indicate stratification in the INL. (J and K) Sox2 and Sox9 (J) or phosphohistone 3 (PH3) and Ki67 labeling (K) of P7 peripheral retinal wild type or Dicer-cKO cross sections; red arrows show migrating RPCs, white areas indicate Ki67+ zones; arrowheads indicate dividing RPCs. P4 wt *n* = 4, P4 cKO *n* = 2, P7 wt *n* = 4, P7 cKO *n* = 4, P14 wt *n* = 4, P14 cKO *n* = 3, P21 wt *n* = 5. Scale bars in D, E: 200 μm, insets: 50 μm; in F–I: 25 μm, in J–K: 50 μm. GCL, ganglion cell layer; IPL, inner plexiform layer; INL, inner nuclear layer; OPL, outer plexiform layer; ONL, outer nuclear layer; NBL, neuroblastic layer; RPE, retinal pigment epithelium.

To label, trace, and analyze the progenies of late RPCs and investigate the impact of Dicer loss in the second phase of retinogenesis (Figure 1A), we used the *Ascl1-CreERT:tdTomato* mouse (referred to as wild type) and crossed it with the Dicer-cKO_RPC_ mouse (referred to as cKO, Figure 1B). *Cre* induction at P1-3 (Figure 1C) resulted in sufficient reporter expression throughout the wild type retina. At P4, the vast majority of cells in the neuroblastic layer (NBL) were reporter+ (Figure 1D). At P7, many Tomato+ cells were found in the inner and outer nuclear layers (INL/ONL); hence, most late-born cells, including rod photoreceptors, were labeled. At P21, very bright Tomato+ cells were found throughout the INL. Reporter signals in photoreceptors in the ONL became fainter over time (Figure 1D), very likely due to the inactivation of Tomato expression in the *Rosa26* locus of mature neurons.^45^^,^^46^

*Cre* activation in the Dicer-cKO mouse at P1-3 led to the successful excision of exon 23 of the Dicer1 gene, resulting in a functionally inactive Dicer enzyme (Figure 1B). This was evident in P4 retinal lysates as well as in all FACS-purified P7 reporter+ cKO RNA-seq samples (reporter-negative or reporter+ wild-type samples had intact *Dicer1*, Figures S1A and S1B). To evaluate whether reporter+ cells were actively dividing RPCs, a single EdU pulse was administered at P3 (Figure 1C). At P4, the vast majority of Tomato+ cells (∼75%) were EdU+ in both wild type and cKO retinas, hence proliferating RPCs (Figures 1E and S1C). No drastic difference was seen in the center and periphery of both conditions. However, it appeared the cKO had slightly more EdU+ cells in the lower NBL. We then traced these cells until P14. EdU+Tomato+ progenies were predominantly found in the ONL (PRs) and the lower and center INL (BCs/ACs/MG) of the central retina of wild types and cKOs. Very few cells were also seen in the upper INL and GCL, and were very likely ACs. Overall, the central and peripheral P14 cKO retinas exhibited a similar pattern of EdU+ cell distribution to that of the wild types. Some areas displayed slightly more cells, but that was not consistent in all sections analyzed (Figures 1E and S1C).

We next performed an initial histological characterization of Tomato+ progenies (indicated via yellow/blue arrowheads) at P7 using the markers Otx2 and Sox9, Prox1, and Vsx2, HuC/D and Pax6, as well as Sox2 and Calbindin (Figures 1F–1I and S2). At this stage, these markers label various cell populations, including RPCs/PCs, young BCs and MG, as well as early and late ACs, and HCs, which are predominantly found in the INL. Many Tomato+ cells were Sox9+ and Otx2+, indicating that at this stage, RPCs, BC precursors/young BCs, and/or young MGs were present. In the cKO, some Tomato+Sox9+Otx2+ cells were also found in the ONL, likely representing migrating RPCs/BC precursors or PR precursors/young PRs. Most Tomato+ ONL cells, however, exhibited a very faint, doughnut-like Otx2 expression pattern, characteristic of the typical photoreceptor expression pattern (and were Sox9-negative, Figures 1F and S2A).

Vsx2 and Prox1, both RPC/BC markers, also labeled the majority of the central INL in both conditions. The cKO, however, appeared to have more cell rows than the wild type (Figures 1G and S2B). Prox1 also labels differentiated ACs found as a single layer in the upper INL, adjacent to the IPL, and HCs in the lower INL, adjacent to the OPL. These populations appeared to be minimally affected in the cKO. However, slightly more, yet fainter, small, round Prox1+ cells were observed in the upper cKO INL, possibly representing young ACs. Additional AC markers, such as Pax6 and HuC/D, were also co-expressed by Tomato+ progenies, in the INL, with more cell rows in the cKO (Figures 1H arrowheads and S2C). It also appeared that some HuC/D cells in the cKO were still migrating up to their location in the INL.

The Calbindin/Sox2 combination revealed that Tomato+Calbindin+ ACs were found in the upper INL in both conditions. Some co-expressed Sox2, hence being cholinergic ACs^47^ (Figures 1I and S2D). However, the cKO seemed to have some additional small Calbindin+Sox2-negative cells in the upper INL. Tomato+Calbindin+ HCs or RGCs were, as expected, not found in the wild type nor cKO. Nevertheless, the stratification in the IPL, which in the wild type showed two distinct synapse lines, was different in the cKO. Three less-defined lines were present, suggesting synaptic alterations early on (Figure 1I, white arrows). Moreover, most Sox2+ progenies were found in the INL, exhibiting a similar pattern to Sox9, and are probably RPCs/young MG. We therefore labeled young MG using Sox9 in combination with glutamine synthetase (GS, Figure S2E). GS was only found in retinal astrocytes in the GCL, which also expressed Sox2 and Sox9. GS was not found in MG at this age, in agreement with previous reports,^48^ and also not ectopically expressed in the cKO.

Since the retinal center is more differentiated, we next evaluated Sox9/Sox2 expression in the periphery. In the wild type, many still migrating Tomato+Sox9+Sox2+ RPCs/PCs with typical elongated nuclei were present in the outermost periphery adjacent to the ciliary body (Figure 1J, arrows). A few of them were still found in the vicinity of the RPE, where RPCs divide (arrowheads). In the Dicer-cKO_RPC_, however, the peripheral Tomato+Sox9+Sox2+ population was larger, with more migrating cells (Figure 1J, arrows). Co-labeling with the proliferation markers Ki67 and phosphohistone 3 (PH3, Figure 1K) showed a greater number of Tomato+Ki67+ cells (arrows) and approximately 2–3 times more Tomato+PH3+ cells in the periphery of cKO retinas compared to wild types (arrowheads). Similar results were found by Davis at al.,^39^ in *αCre* mice at embryonic stages, suggesting extended cell division events after miRNA loss.

**Figure 2:**
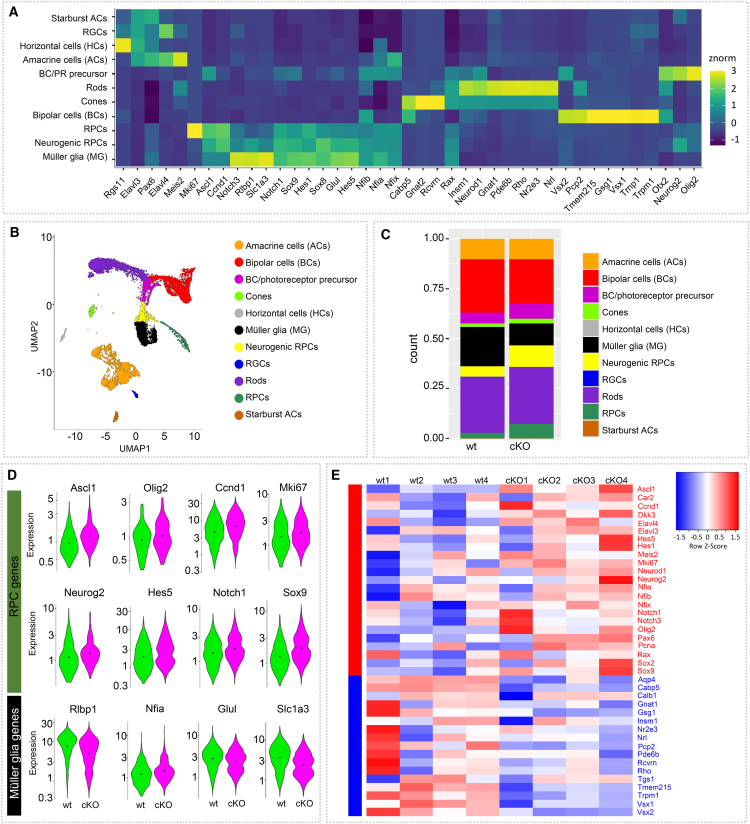
Dicer loss in late RPCs results in less mature progenies (A–D) scRNA-seq of FACS-purified P7 Tomato+ wild type (wt) and Dicer-cKO (cKO) progenies showing (A) a heatmap of expressed genes in annotated cell types, (B) UMAP-dimension reduction, and (C) cell proportions, colored by annotated cell type as determined by marker gene expression; (D) Violin plots of the cellular expression of marker genes of RPCs and Müller glia (MG), facetted by genotype; WT: *n* = 3 mice, pooled, cKO: *n* = 3 mice, pooled, one technical replicate. See also additional data in Figure S3. (E) Heatmap of selected 40 up- and downregulated genes of bulk RNA-seq FACS-purified P7 Tomato+ wild type and cKO progenies; wt: *n* = 4 biological replicates, cKO: *n* = 4 biological replicates, 4 independent experiments per condition, normalized counts, see also Table S1. RPCs, retinal progenitor cells; RGCs, retinal ganglion cells; PRs, photoreceptors.

Since the different markers used are not cell-type specific at this age, and histological characterizations are therefore limited, we performed single-cell and bulk RNA-seq to elucidate better the P7 cell populations and subpopulations, as well as their gene expression patterns (Figures 2 and S3). We FACS-purified P7 Tomato+ wild-type and cKO progenies, as well as collected the corresponding reporter-negative fractions of both conditions for scRNA-seq. Cell types/populations were identified based on known marker expression for retinal cell types^48^^,^^49^^,^^50^ (Figure 2A). The predominant cell populations found in the FACS-purified reporter+ wild type retinas included the expected late-born RPC progenies, i.e., rods, BCs, and MG. We also found BC/photoreceptor precursors, neurogenic RPCs, and RPCs (Figure 2B). Another separate, quite large population consisted of ACs. HCs, RGCs, and Starburst ACs, i.e., early-born cells, were not present or only as a tiny population within the Tomato-negative fraction (Figure S3A).

We next compared the cell proportions found in P7 wild type progenies with those of the cKO progenies. Overall, scRNA-seq confirmed our histological findings. The cKO had larger proportions of immature cells, i.e., RPCs, neurogenic RPCs, and BC/rod precursors (Figures 2C and S3A). Fractions for mature BCs and MG were smaller than those of the wild type, suggesting maturation deficits of these two populations. The fractions of rod and AC populations were, however, similar in both conditions. Wild type non-progenies (Tomato-negative cells) contained a large AC fraction, Starburst ACs, HCs, and RGCs. cKO non-progenies (Tomato-negative cells) contained a smaller number of ACs, only a few HCs, RGCs, and Starburst ACs (Figure S3A). This suggests that these cell populations may be indirectly affected by the loss of Dicer in late RPCs (e.g., missing proper connections), potentially impacting their health as well. We also found a small fraction of cones among our wild type and cKO progenies. Since cones are only born during embryonic stages, we believe that this result is due to cell carryover. MGs are very tightly associated with/wrap both PR types, resulting often in some PR contamination of FACS-purified cells with cytoplasmic reporter proteins.^51^

We next analyzed and compared the expression patterns in the specific populations of wild type and cKO progenies, starting with RPC/precursor genes and MG genes. This differentiation is not always easy since MG express many RPC genes.^52^^,^^53^ Interestingly, *Ascl1*, *Olig2*, *Neurog2*, and *Sox9*, all RPC/precursor genes as well as cell cycle genes, such as *Mki67* and *Ccnd1*, were upregulated in the cKO (Figures 2D and S3B). By contrast, known MG markers, including *Rlbp1*, *Nfia*, *Glul*, and *Slc1a3*^48^^,^^50^ showed reduced expression levels.

In addition to scRNA-seq, we also conducted bulk RNA-seq of FACS-purified P7 progenies. Although this dataset represents a heterogeneous population, bulk RNA-seq allows a better sequencing depth and validation. The heatmap in Figure 2A shows 30 selected genes known to be specific to RPCs, BCs, PRs, MG, and ACs.^48^^,^^50^^,^^54^^,^^55^^,^^56^ This selection also includes genes whose protein expression we have evaluated over time via histological analysis. We identified RPC/precursor genes (e.g., *Ascl1*, *Hes1*, *Rax*, *Dkk3*), including cell cycle genes (e.g., *Ccnd1*, *Mki67*, *Pcna*), which were upregulated (Figure 2E; Table S1). Also upregulated were *Meis2*, *Pax6*, *and Cdkn1b (encodes for* p57kip2*)* genes known to play a role in RPC state as well as AC fates.^57^^,^^58^^,^^59^^,^^60^^,^^61^ Furthermore, we found *Elavl3* and *Elavl4* upregulated, both genes with essential functions during development and AC formation.^62^^,^^63^^,^^64^^,^^65^^,^^66^ In contrast, genes of the late-born cells, including MG (*Aqp4*), BCs (*Cabp5*, *Insm*, *Vsx1*, *Tmem215*, *Gsg1*), and rods (*Gnat2*, *Rcvrn*, *Nrl*, *Nr3e3*, *Rho*), were downregulated in the cKO compared to the wild type (Figure 2E; Table S1). Apoptosis genes, including *Caspases* or *Bax*, were not found to be upregulated in P7 cKOs, suggesting no increased apoptosis at least at this stage (Table S1). This is different from observations made in embryonic Dicer loss studies.^36^^,^^38^

### P56 cKO mice have fewer Müller glia and an unknown putative immature population

**Figure 3:**
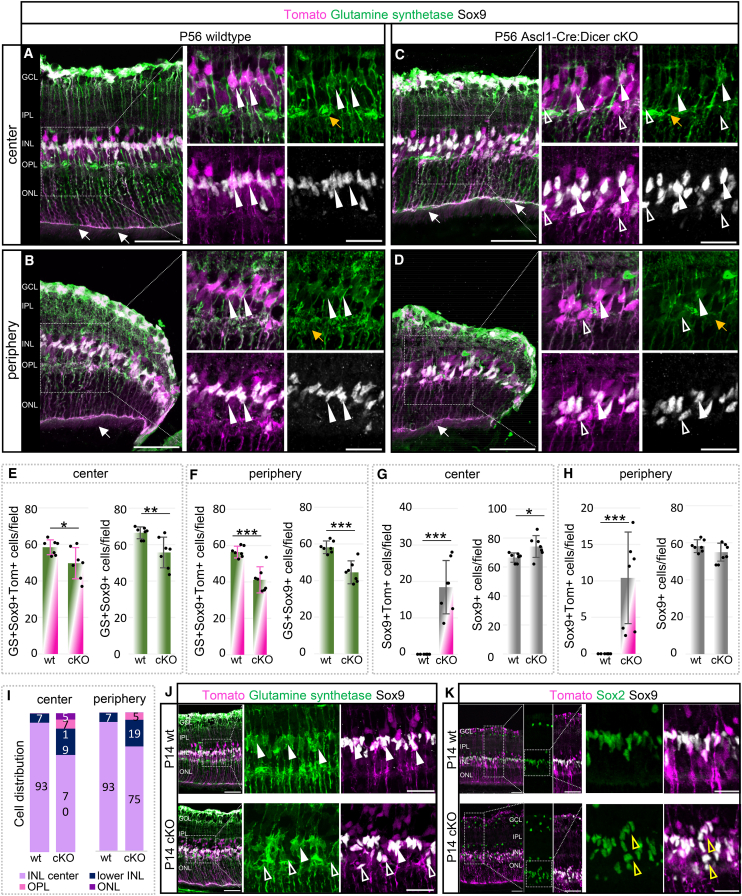
Dicer loss in late RPCs results in reduced Müller glia population and a new, unknown population (A–D) Müller glia (MG) labeling with antibodies against glutamine synthetase (GS) and Sox9 of central or peripheral P56 wild type (wt) or cKO retinas, with insets shown in high magnification; white arrowheads indicate GS+Sox9+ MG, unfilled arrowheads GS-negative Sox9+ cells, white arrows the external limiting membrane (ELM), yellow arrows alterations in MG processes. Markers depicted in white are labeled in black font. (E and F) Absolute number of Tomato+GS+Sox9+ MG and total GS+Sox9+ MG per field in the central or peripheral retina. (G and H) Absolute overall number of Tomato+Sox9+ cells and total number of Sox9+ (GS-) per field of the central or peripheral retina. (I) Tomato+Sox9+ cell distribution (percentage) in the center INL, lower INL, OPL, and ONL of the central and peripheral wild type and cKO retina. (J and K) GS and Sox9 (J) and Sox2 and Sox9 (K) labeling of P14 wild type and cKO central retinas, with higher magnification insets; white arrowheads show Sox9+GS+ MG, unfilled arrowheads Sox9+GS-negative cells, yellow arrowheads stacked cells; P14 wt *n* = 4, P14 cKO *n* = 3, P56 wt: *n* = 7, P56 cKO: *n* = 7, mean ± S.D., Mann-Whitney-U-test: ∗: *p* ≤ 0.05; ∗∗: *p* ≤ 0.01; ∗∗∗: *p* ≤ 0.001. Scale bars in A–D, in insets: 25 μm, scale bars in J–K: 50 μm, in insets and higher magnified insets: 25 μm. The layer description is given in Figure 1.

Based on our *in vivo* data, we chose P56 as the time point of in-depth analysis of the adult retina. Since MG seemed to be affected and play a crucial role in overall retinal health, we next examined adult cKO retinas concerning their cellular composition of the glia. To evaluate the total number of MG and the fractions of RPCs that differentiated into MG, we labeled P56 tissue with antibodies against glutamine synthetase (GS) and Sox9 (Figures 3A–3D). Wild type MG somata were almost exclusively located in the center of the INL (arrowheads), in central and peripheral areas. The MG processes spanned the entire retina to form the inner and outer limiting membranes (arrows), confirming that the vast majority of MG was labeled using this reporter mouse (Figures 3A and 3B).

In the Dicer-cKO_RPC_, central and peripheral retinal areas had many displaced and scattered Sox9+ cells throughout the INL, OPL, and ONL (Figures 3C and 3D). Most cells were GS+ MG (arrowheads). These glia, however, did not have fine, branched processes in the OPL (particularly in peripheral areas, indicated by yellow arrows), suggesting possible maturation deficits. Nevertheless, the OLM was formed and seemed intact (white arrows). Cell counts of the total number of Tomato+ MG as well as overall MG numbers in the central and peripheral cKO retina showed a ∼15% and ∼25% decline of both populations, respectively, compared to the wild type (Figures 3E and 3F). 90% of all MG found in the wild type or cKO were reporter+, and the fraction of Tomato-labeled MG was similar in wild type and cKO, with approximately 90%–97%.

Some of the displaced Sox9+ cells (ONL) in the cKO had elongated nuclei. They were GS-negative (Figures 3C and 3D, unfilled arrowheads), suggesting a putative immature cell population that did not migrate to its intended destination (residual RPCs). This population comprised approximately one-fourth of all Sox9+ cells in the center and about one-sixth in the periphery. The overall number of Sox9+ cells in the cKO was, however, not much different from that in wild types, suggesting that these displaced cells are very likely immature cells that failed to differentiate into glia, resulting in their ectopic location in the outer retina (18%–23% displaced cells, Figures 3G and 3H, the layer distribution (%) is shown I). Interestingly, a similar phenotype, i.e., dislocated Sox9+ cells, mostly GS-negative MG, has been reported previously in an MG-specific Dicer-cKO.^67^ Although in the MG study, young differentiated MG were targeted and manipulated, which differs from the present study in which RPC/PCs, including MG progenitors/precursors, were targeted and manipulated, possible dedifferentiation events could cause this outcome, at least in the central (more mature) retina of this RPC-Dicer-cKO study.

Since we could not detect any GS expression in P7 MG (Figure S2E), we next analyzed P14 tissue to ascertain whether these Tomato+Sox9+GS-negative cells were present early on (Figure 3J, unfilled arrowheads). We found displaced Tomato+Sox9+ cells (GS-negative) in the P14 cKO, but not in the wild type. These displaced cells were also Sox2+ and were partly arranged in stacks (Figure 3K, unfilled arrowheads), presumably representing remaining resident RPCs. Interestingly, similar progenitor stacks/columns were also seen in embryonic Dicer-cKO studies.^39^

### miRNA loss in late retinal progenitor cells results in impaired rod photoreceptor function but does not affect cones

**Figure 4:**
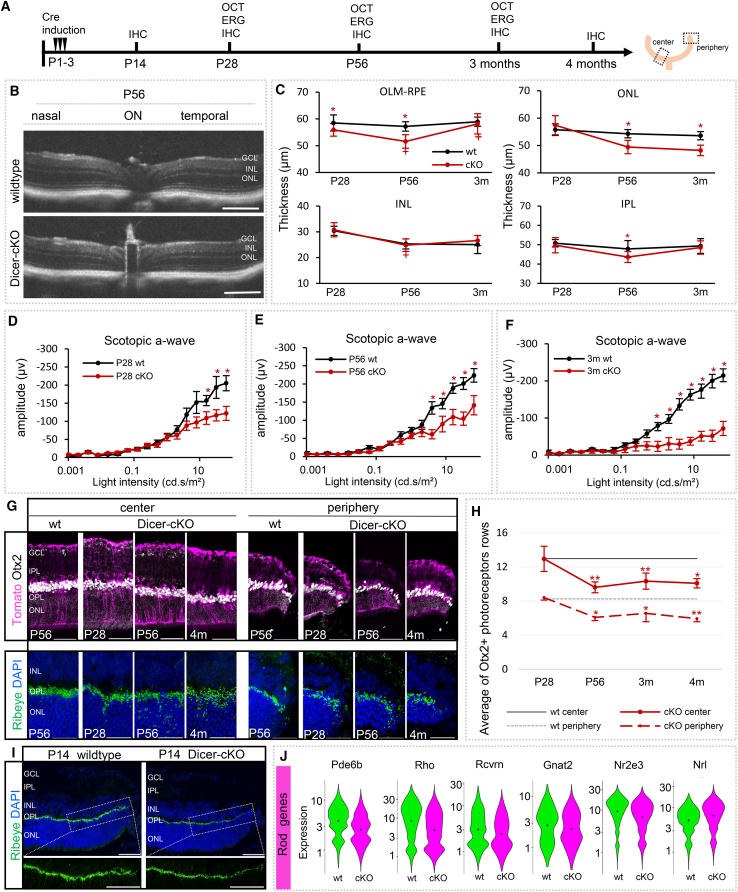
Dicer loss in late RPCs leads to reduced rod function (A) Experimental design and timeline. (B) Optical coherence tomography (OCT) images of wild type (wt) and cKO center retinas at the nasal-temporal axis. (C) Timeline plots showing specific layer thickness changes in OCT for P28 wt (*n* = 13) and cKO (*n* = 10), P56 wt (*n* = 8) and cKO (*n* = 9), and 3-month wt (*n* = 8) and cKO (*n* = 4), mean ± S.D. See also additional data in Figures S4 and S5. (D–F) Full-field scotopic electroretinogram recordings showing a-wave amplitudes of wt and cKO mice at P28 (wt: *n* = 9 vs. cKO: *n* = 10, D), P56 (wt: *n* = 11 vs. cKO: *n* = 7, E), and 3 months (wt: *n* = 5 vs. cKO: *n* = 5, F), mean ± S.E.M. See additional data in Figure S6. (G) Antibody labeling against Otx2 or Ribeye, as well as DAPI nuclear staining of central or peripheral wt or cKO retinas at P28, P56, and 4 months. Markers depicted in white are labeled in black font; additional data are shown in Figure S7. (H) Time course of the number of Otx2+ photoreceptor (PR) rows (10 averaged values per image) in the central or peripheral ONL in wt (*n* = 8, baseline), P28 (*n* = 4), P56 (*n* = 6), 3-month (*n* = 4), and 4-month-old cKO mice (*n* = 4), mean ± S.D. (I) Antibody labeling against Ribeye of P14 wt (*n* = 4) or cKO (*n* = 3) peripheral retinas with higher magnified insets. (J) Violin plots of the cellular expression of marker genes of rod photoreceptors in scRNA-seq FACS-purified P7 progenies (*n* = 3 mice per condition, on technical replicate). Mann-Whitney-U-test: ∗*p* ≤ 0.05, ∗∗*p* ≤ 0.01. Scale bars in B: 200 μm, in G upper Otx2 section: 50 μm, lower Ribeye section: 25 μm, in I: 50 μm. Layer description: see Figure 1.

To investigate the observed impact of this maturation delay on structural and functional outcomes in the adult cKO mouse, we performed SD-OCT and ERGs, paired with histological evaluations at P28, P56, as well as 3 and 4 months of age (Figure 4A). SD-OCT was conducted at the central retinas of approximately 650 μm radius from the optic nerve (ON). Measurements were performed across the nasal-temporal and superior-inferior axes (Figures 4B, 4C, S4, and S5). The OCT scans of wild types across the different ages showed intact retinas with a total thickness of about 220 μm, in accordance with previous reports.^68^^,^^69^^,^^70^ Somewhat surprisingly, the OCT of P28 Dicer-cKO_RPC_ mice appeared normal, and the overall retinal thickness was also not much altered later on. However, initial alterations were found at the layer level, starting at P28 and affecting the OLM-RPE area. This area comprises the outer/external limiting membrane (OLM/ELM), the inner and outer segments (IS/OS) of photoreceptors, and the RPE. We found a ∼5% reduction of this area in the cKO retinas (*p* = 0.03), suggesting photoreceptor segment impairments. The ONL, however, which contains the photoreceptor cell bodies, did not show any significant alteration at P28 (*p* = 0.08). Hence, most photoreceptors were formed and present at that age. However, starting at P56, signs of degeneration were trackable. We found a ∼14% reduction in the thickness of the OLM-RPE compared to wild types, suggesting an increased impairment of photoreceptor segments (Figure 4C). The ONL displayed an initial thinning (∼11% decrease) in P56 Dicer-cKO_RPC_ mice that remained at that level at 3 months of age (*p* = 0.414). This photoreceptor loss, however, seemed to progress slowly. Notably, the initial loss of thickness of the OLM-RPE seemed “restored” in 3-month cKOs. However, fluctuation in the thickness of the subretinal space (OLM-RPE) is a characteristic during retinal degeneration, very likely due to swelling in this area (edema formation) or accumulation of cellular debris.^71^^,^^72^^,^^73^ No drastic alterations were found in the inner retinal layers, except for the IPL, suggesting impairment of neuronal processes at P56 and potential swelling events in 3-month cKOs.^73^

To measure rod photoreceptor function, scotopic ERGs were conducted (Figures 4D–4F and S6). Wild types displayed normal healthy responses, with a scotopic a-wave amplitude of about −200 μV. Scotopic a-wave amplitude recordings in Dicer-cKO_RPC_ mice showed a significant reduction to ∼130 μV at P28, indicating impairment of photoreceptor function. Amplitudes remained unchanged in P56 cKOs mice but further dropped in 3-month-old mice (∼75 μV).

Since the P28 Dicer cKO exhibited no structural impairments in the ONL (unchanged thickness via OCT), all photoreceptors (cell bodies, histology) were generated; however, they apparently were not fully functional (differentiated). Furthermore, the additional functional loss at 3 months of age could suggest the onset of secondary neuronal degeneration, as reported before in an embryonic Dicer loss study.^34^ This functional loss may be caused by the immaturity of the cells, as no cell loss was detected via OCT.

To analyze photoreceptor impairment at the histological level, we labeled retinal cross sections of various ages with the markers Otx2 (photoreceptor nuclei) and Ribeye (ribbon synapses, Figures 4G–4I and S7A–S7D). We first evaluated the number of Otx2+ photoreceptor rows in the central and peripheral regions of the retina. While P28 cKO retinas appeared intact, a 26% reduction in the ONL was found at P56 in both center and periphery. However, no further cell loss was evident up to 4 months of age (Figure 4H), confirming our OCT data.

The evaluation of ribbon synapses showed dense synapses restricted to the OPL in the center and the peripheral wild type retina (Figures 4G and S7B) In the P28 Dicer-cKO_RPC_, however, ribbon synapses were more diffuse, not only found in the OPL but also in the ONL. In P56 up to 4-month-old cKO mice, they seemed more and more diffuse and scattered throughout the OPL and ONL, predominantly in the central retina. This suggests remodeling or rewiring events. To determine whether these synaptic impairments were already present at a younger age, we evaluated P14 tissue (Figures 4I, S7C, and S7D). In the wild type, Otx2 expression appeared normal, and a clear band of ribbon synapses was formed in the central OPL. In the periphery of Dicer-cKO_RPC_ retinas, Otx2 also seemed to be normal. Ribeye, however, exhibited reduced or absent signals. This suggests that peripheral ribbon synapses were not correctly formed, which may have led to progressive structural impairments and functional defects in the adult retina that spread from the periphery toward the center. Our transcriptomic analysis supported these findings. As mentioned before, the overall proportions of rods in the scRNA-seq datasets were strikingly similar in wild type and cKO (s 2C). Nevertheless, rod gene transcripts were found to be reduced in bulk RNA-seq FACS-purified P7 cKO progenies (Figure 2E; Table S1). scRNA-seq further confirmed this finding, showing reduced levels of essential rod genes, including *Pde6b*, *Rho*, *Rcvrn*, *Gnat2*, *Nr2e3*, *or Nrl*^48^^,^^50^^,^^54^^,^^55^^,^^56^ in the P7 cKO rod progeny (Figure 4J).

**Figure 5:**
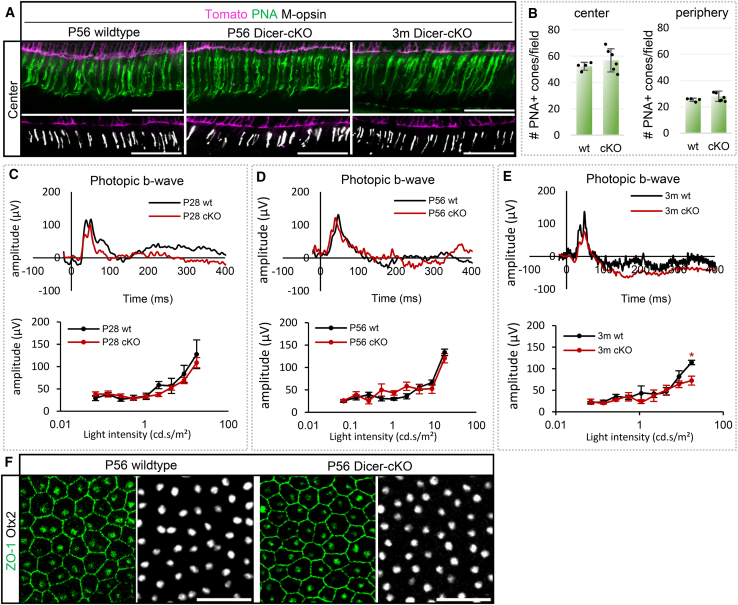
Cone photoreceptors display normal function in young Dicer-cKO mice (A) Antibody labeling against peanut agglutinin (PNA) or M-opsin of central P56 wt or cKO retinas. (B) Absolute number of PNA+ cone segments per field in P56 wt (*n* = 6) and cKO retinas (*n* = 6), mean ± S.D., *p* = 0.454 and 0.412, respectively. Additional data are shown in Figure S7. (C–E) Full-field photopic electroretinogram recordings showing b-wave amplitudes as selected wave forms and intensity-dependent graphs for P28 (C, wt: *n* = 9 vs. cKO: *n* = 10), P56 (D, wt: *n* = 11 vs. cKO: *n* = 7), and 3-month-old (E, wt: *n* = 5 vs. cKO: *n* = 5) wt and cKO mice, mean ± S.E.M., Mann-Whitney-U-test ∗: *p* ≤ 0.05. See additional data in Figure S6. (F) ZO-1 and Otx2 antibody labeling of P56 wt (*n* = 5) or cKO RPE (*n* = 5). Scale bars in A, F: 50 μm. Layer description: see Figure 1. Markers depicted in white are labeled in black font.

Next, we evaluated cone photoreceptor health using peanut agglutinin (PNA) and M-opsin. The density and pattern of PNA+ and/or M-Opsin+ cone segments in wild type and P56 or 3-month Dicer-cKO_RPC_ retinas were not different (Figures 5A, 5B, and S7E). Furthermore, the photopic b-wave in P28 and P56 cKOs was similar to that of wild types (about 150 μV), suggesting intact, healthy cones (Figures 5C–5E and S6). Hence, early-born cones were not affected in our Dicer-cKO. In 3-month-old Dicer-cKO_RPC_ mice, however, initial functional cone impairments were detected, possibly as a response to the malfunctioning and/or reduced rods at P56 or due to MG impairment,^70^ or both.

To exclude the possibility that the RPE contributed to the observed photoreceptor impairment phenotype as seen in RPE-specific^74^^,^^75^ and MG-specific Dicer-cKO studies,^70^ we examined flat-mounted RPE at P56. We used ZO-1, a marker for the structural and functional integrity of tight junctions (TJs) in the RPE, and Otx2, a marker for RPE nuclei (Figure 5F). There was no reporter expression found in the RPE of wild type and Dicer-cKO mice. The RPE of P56 Dicer-cKO mice was intact and similar to wild type RPE; hence, a direct contribution of the RPE in photoreceptor loss was ruled out.

### Dicer loss in late retinal progenitor cells leads to a reduction in bipolar cell number and functional deficits

We next analyzed BCs, which are also located in the INL and which form between P0 and P7. According to our P7 transcriptomic data, the cKO BC precursor population was increased at the expense of the mature BC population (Figure 2C), very likely resulting in maturation delays and functional impairments. Furthermore, as mentioned before, BC gene transcripts were reduced in the cKO (bulk RNA-seq, Figure 2E).

**Figure 6:**
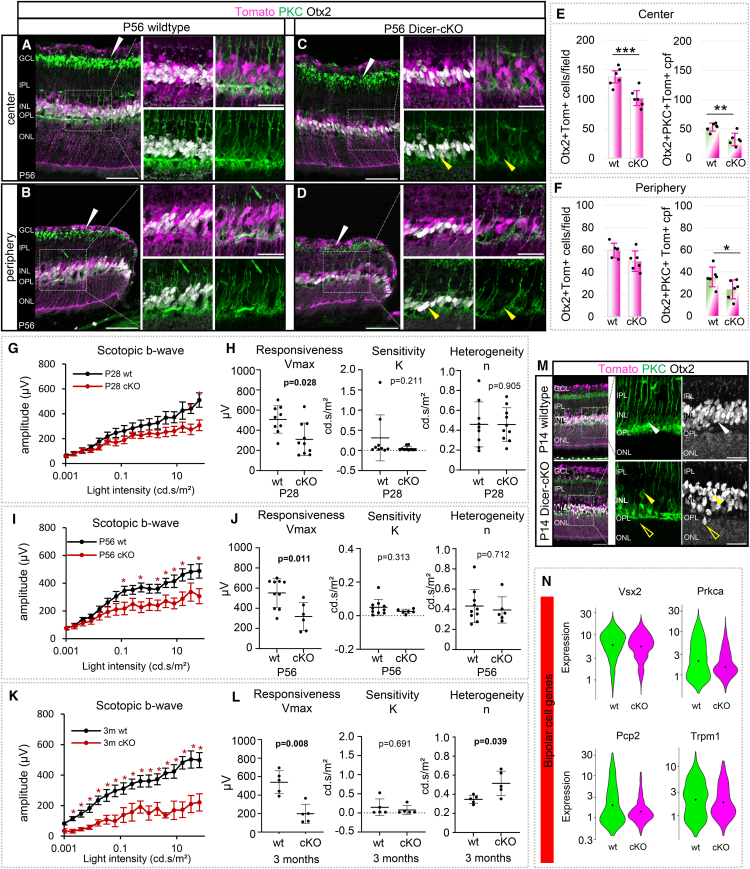
Dicer loss in late RPCs leads to a reduced bipolar cell population and functional defects in the inner retina (A–D) Antibody staining to label Otx2+ overall bipolar cells (BCs) and PKC+ rod BCs of central or peripheral P56 wt or cKO retinas, with insets showing higher magnification; white arrowheads show PKC+ dendrites in IPL; yellow arrowheads show the absence of rod BCs. Markers depicted in white are labeled in black font; additional data are shown in Figure S8. (E and F) Absolute number of Tomato+Otx2+ BCs and Tomato+PKC+Otx2+ rod BCs per field in the central or peripheral retina; wt: *n* = 6, cKO: *n* = 6, mean ± S.D. (G–L) Full-field scotopic electroretinogram recordings showing b-wave amplitudes of P28 (G), P56 (I), and 3-month-old (K) wt and cKO mice. Estimated saturated amplitudes (Vmax, responsiveness), semi-saturation (K, sensitivity), and slope (n, heterogeneity) using the Naka-Rushton equation for P28 (H), P56 (J), and 3-month-old mice (L), P28 wt: *n* = 9 vs. cKO: *n* = 10; P56 wt: *n* = 11 vs. cKO: *n* = 7; 3m wt: *n* = 5 vs. cKO: *n* = 5, mean ± S.E.M. See additional data in Figure S6. (M) PKC and Otx2 antibody labeling of central and peripheral P14 wt (*n* = 4) or cKO (*n* = 3) retinas with insets showing higher magnification; white arrowhead shows wt rod BC, yellow arrowheads displaced rod BCs; unfilled yellow arrowheads displaced PKC-negative Otx2+ cells. (N) Violin plots of the cellular expression of marker genes of BCs in scRNA-seq FACS-purified P7 progenies (*n* = 3 mice per condition, on technical replicate). Significant differences are indicated, Mann-Whitney-U-test: ∗: *p* ≤ 0.05; ∗∗: *p* ≤ 0.01; ∗∗∗: *p* ≤ 0.001). Scale bars: A–D: 50 μm, insets: 25 μm, in M: 50 μm; insets: 25 μm. Layer description: see Figure 1.

Although OCT scans did not reveal any cell loss in the INL, we evaluated the overall BC population histologically using antibodies against Otx2 and PKC at P56, the time point at which we found an impaired IPL (Figures 6A–6D and 4C). In the adult retina, Otx2 is a generic BC marker, while PKC labels specifically rod BCs, which constitute approximately one-third of the overall BC population.^76^^,^^77^ In the wild type and Dicer-cKO_RPC_ retinas, Otx2+ BCs were found in the lower INL, adjacent to the OPL, in the central and peripheral retina. The vast majority (88%) of all BCs were reporter+ in both areas. However, the total number of Tomato+Otx2+ BCs was reduced in the cKO compared to the wild type (−26%), and a trend toward reduction was seen in the periphery as well (Figures 6E and 6F). 15%–20% of all Otx2+ BCs were reporter-negative, possibly due to incomplete reporter recombination, cells not being affected by the Dicer-cKO, or loss of their fluorescence tag. Nevertheless, the decrease in BC progenies resulted in a reduction in the overall BC population in the cKO (center: approximately −26%, periphery: approximately −23%; Figure S8).

The most affected BC type appeared to be the rod BCs. Rod BC somata (PKC+Otx2+) are located in the lower INL. Their processes and dendrites, which are beautifully visualized via PKC, extend to the GCL as well as to the OPL, in both the central and peripheral retina (Figures 6A and 6B, white arrowheads). In the P56 Dicer-cKO_RPC_, rod BCs were present, but their number appeared reduced, and some were dislocated (yellow arrowheads). Furthermore, their processes appeared less dense and less developed (Figures 6C and 6D, white arrowheads). The quantification of the overall number of Tomato+PKC+Otx2+ rod BCs revealed a 40% and 32% reduction in the center and periphery, respectively (Figures 6E and 6F). This deficit resulted in an overall rod BC reduction of 42% in the center and 35% in the periphery in P56 Dicer-cKO_RPC_ retinas (Figure S8).

We next evaluated rod BC function (Figures 6G–6L and S6). In the Dicer-cKO_RPC_, as early as P28, the scotopic b-wave was reduced (∼300 μV vs. wt: ∼550 μV), very likely due to the reduced observed a-wave response. An in-depth analysis using the Naka-Rushton equation revealed a significantly reduced Vmax, i.e., reduced responsiveness. Sensitivity, reflected by the semi-saturation constant (K) as well as heterogeneity, reflected by the slope (n), were, however, surprisingly like wild type values at P28 and P56 (Figures 6H and 6J). In 3-month-old Dicer-cKO_RPC_ mice, the scotopic b-wave amplitudes were approximately 200 μV, indicating a trend toward further functional reduction. 3-month-old cKO mice had a reduced Vmax and a higher slope n, suggesting a potential secondary neuronal degenerative event in the INL at this age, or MG malfunction (Figure 6L).

To determine whether this phenotype onset was observed early, P14 retinal cross sections were evaluated (Figure 6M). Overall, P14 wild type retinas displayed a similar Otx2 and PKC expression pattern as seen in adult wild types. P14 cKO retinas, however, had fewer dense Otx2+ cells (likely due to cell loss), and some cells were found in ectopic locations within the ONL. Rod BCs were reduced, and some were misplaced (Figure 6M, arrowheads) and PKC-negative (unfilled arrowheads). The PKC+ dendrites in the IPL were less dense/less developed, overall displaying the phenotype seen at P56 and confirming developmental defects. Hence, the aforementioned downregulated BC genes (e.g., *Cabp5)* in P7 cKO progenies (bulk RNA-seq, Figure 2E) seemed to be the cause of this outcome. scRNA-seq of P7 BC progenies confirmed these bulk sequencing findings, showing a reduction in important BC genes, including *Prkca* (which encodes PKC), *Vsx2*, *Trpm1*, *Pcp2*, as well as *Cabp5*, *Insm1*, and *Gag1*, in the cKO BC population (Figures 6N and S3B).

### P56 Dicer-cKO retinas have increased amacrine cell numbers

**Figure 7:**
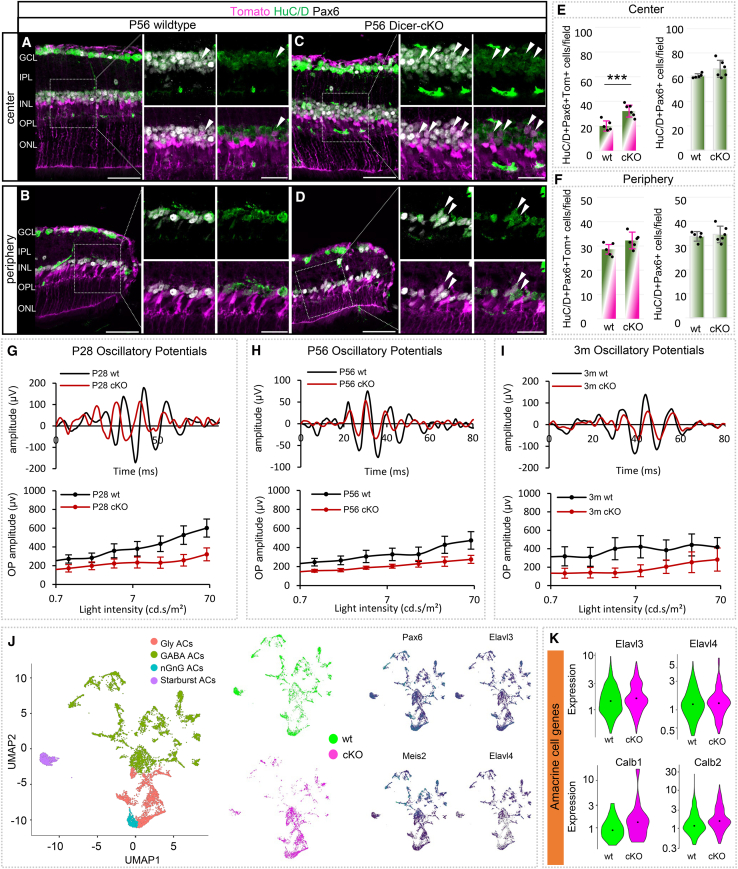
Dicer loss in late RPCs results in an increased amacrine cell population (A–D) Antibody staining against HuC/D and Pax6 to label amacrine cells (ACs) in the central or peripheral P56 wt or cKO retina, with insets in higher magnification; white arrows show AC progenies, partially arranged in clusters in the cKO. Markers depicted in white are labeled in black font. (E and F) Absolute number of Tomato+ Pax6+ HuC/D+ ACs and the overall number of Pax6+HuC/D+ ACs per field in the central or peripheral retina; wt: *n* = 5, cKO: *n* = 6, mean ± S.D. (G–I) Oscillatory potential (OP) amplitudes of full-field scotopic electroretinogram recordings showing selected wave forms and intensity-dependent graphs for P28 (G, wt: *n* = 8, cKO: *n* = 5), P56 (H, wt: *n* = 10, cKO: *n* = 6), and 3-month-old wt and cKO mice (I, wt: *n* = 5, cKO: *n* = 4), mean ± S.E.M. See additional data in Figure S6. (J) UMAP-dimension reduction of scRNA-seq (one technical replicate) FACS-purified integrated P7 Tomato+ wild type (WT, *n* = 3) and Dicer-cKO (cKO, *n* = 3) progenies, colored by annotated cell population or cell type as determined by marker gene expression, with representation of selected AC transcripts. (K) Violin plots of the cellular expression of selected AC transcripts in scRNA-seq P7 progenies (*n* = 3 mice per condition, on technical replicate), additional data are shown in Figure S9. Significant differences are indicated, Mann-Whitney-U-test: ∗: *p* ≤ 0.05; ∗∗: *p* ≤ 0.01; ∗∗∗: *p* ≤ 0.005. Scale bars in A–D, insets 25 μm. Layer description: see Figure 1.

Since we found some additional faint Prox1+ or Calbindin+ cells in the upper INL in P7 cKOs, we next investigated the ACs in the adult retina. Adult ACs were stained with antibodies against HuC/D and Pax6, both markers that are also in retinal ganglion cells (RGCs) in the GCL (Figures 7A–7D). Similarly, as in P7 retinas, P56 cKO retinas had 1-2 more Pax6+ and HuC/D+ cell layers in the INL (wt center 2–3 Pax6+ HuC/D+ cell layers, wt periphery: 1–2 Pax6+ HuC/D+ cell layers). All HuC/D+ cells were Pax6+, while very few Pax6+ wells were HuC/D-negative and likely MG.^78^^,^^79^^,^^80^ Many of these ACs were Tomato+ (arrowheads) and may have partially compensated for INL cell loss (BCs), which could explain why OCT scans did not show any reduction in the INL. The quantification of the total number of Tomato+HuC/D+Pax6+ ACs in the INL showed a 60% increase in reporter+ ACs in the central cKO retina, and a trend toward an increase (10%) in the periphery compared to wild types (Figures 7E and 7F). The overall amacrine cell population, however, was stable with a trend toward an increase, probably because the vast majority of ACs (∼67%) were generated before the manipulation.

To investigate whether this ectopic cell population impacts retina function, oscillatory potentials (OPs) were evaluated (Figures 7G–7I and S6). OPs reflect inner retinal activity, predominantly neuronal synaptic activity in inhibitory feedback pathways mediated by ACs. They exhibit rapid changes in adaptation and represent both photopic and scotopic processes, hence depend on photoreceptor and BC input. The OPs of wild types across all ages analyzed were approximately the same, with amplitudes ranging between 400 and 600 μV. The OPs in the cKO mice did not show any significant differences (*p* values for highest intensities obtained: P28 wt vs. cKO: *p* = 0.065; P56 wt vs. cKO: *p* = 0.181, 3m wt vs. cKO: *p* = 0.786). However, a trend toward a reduced amplitude (300 μV) was observed at all ages. This is very likely predominantly due to the decreased input (a- and b-wave, Figures 4D–4F and 6G–6L^81^). It, however, could also suggest possible amacrine cell impairment/rewiring events.

Since we identified some ectopic ACs in our adult cKOs, we extracted the AC-progeny population from the P7 scRNA-seq dataset to evaluate its composition in the wild type and cKO (Figures 7J and 7K). The major populations found within the P7 wild type AC fraction based on marker expression (Figure S9A) included glycinergic (born E16-P1) and GABAergic ACs (born E14-P0) as well as cholinergic Starburst ACs (born E8-E16) and non-GABAergic non-glycinergic ACs (nGnG, born postnatally, Figure 7J).^82^^,^^83^ Interestingly, the comparison of cell counts and proportions found in cKOs showed alterations in both the absolute and relative numbers (Figures S9B and S9C). The predominant cKO AC populations were glycinergic and nGnG ACs (Figures 7J, S9B, and S9C), both of which were late-born. Furthermore, overall, the GABAergic (early-born) ACs were reduced, but not only within the progenies (Tomato+) but also within the non-progenies (Tomato-negative, Figure S9B). This could suggest not only a direct but also an indirect impairment of GABA-signaling in the cKO. Glycinergic (late-born) ACs, on the other hand, were the dominating AC population in the P7 cKO. nGnG ACs, though (also late-born), seemed reduced (Figures S9B and S9C).

As mentioned earlier, among the genes upregulated in the P7 cKO bulk RNA-seq dataset were the AC genes *Elavl3* and *Elavl4* (Figure 2E), which encode HuC and HuD, respectively. Not much is known about *Elavl3/4* in the retina. However, *Elavl* genes are highly conserved across species^84^^,^^85^^,^^86^ and play a role in neuronal differentiation, maintenance, and axogenesis in the brain, as well as AC subtype differentiation during retinogenesis for maintaining normal retinal function in the retina.^63^^,^^65^

P7 scRNA-seq revealed that *Elavl3* was expressed in all AC subtypes together with *Pax6*, *Meis2*, and *Calb2* (encodes for Calretinin, Figures 7J and S9A). It seemed to have somewhat higher levels in the glycinergic and nGnG ACs, though. *Elavl4* was also found across all AC populations, yet it appeared to be more restricted to GABAergic ACs, which co-expressed Slc6a1 (encoding GABA transporter 1, GAT1) and Gad1 (encoding glutamic acid decarboxylase (GAD) 67). A quantification of the cell counts and proportions of the wild type AC progenies revealed that the major populations found were glycinergic (largest), GABAergic, and nGnG ACs (Figures 7K and S9B). In the cKO, an even larger glycinergic but smaller nGnG population was found compared to wild types (Figures 7K, S9B, and S9C). Therefore, although the overall AC fraction/population in the P7 cKO was similar to that in wild types (Figure 2C), the composition of cKO AC subtypes was different. This suggests a role of Dicer/mature miRNAs in AC subtype specification. Moreover, the overall number of captured ACs in the cKO (Tomato+ and Tomato-negative cells) was lower than in the wild type (Figure S9C). Since we found higher cell numbers histologically, the cKO ACs (even those without the reporter) may be more fragile/impaired and have died during cell sorting. Nevertheless, the evaluation of the AC gene expression in these P7 cKO AC-progenies showed increased levels of *Elavl3 and Elavl4*, as well as *Calb1* and *Calb2* (Figure 7K), confirming our bulk RNA-seq data (Figure 2E).

**Figure 8:**
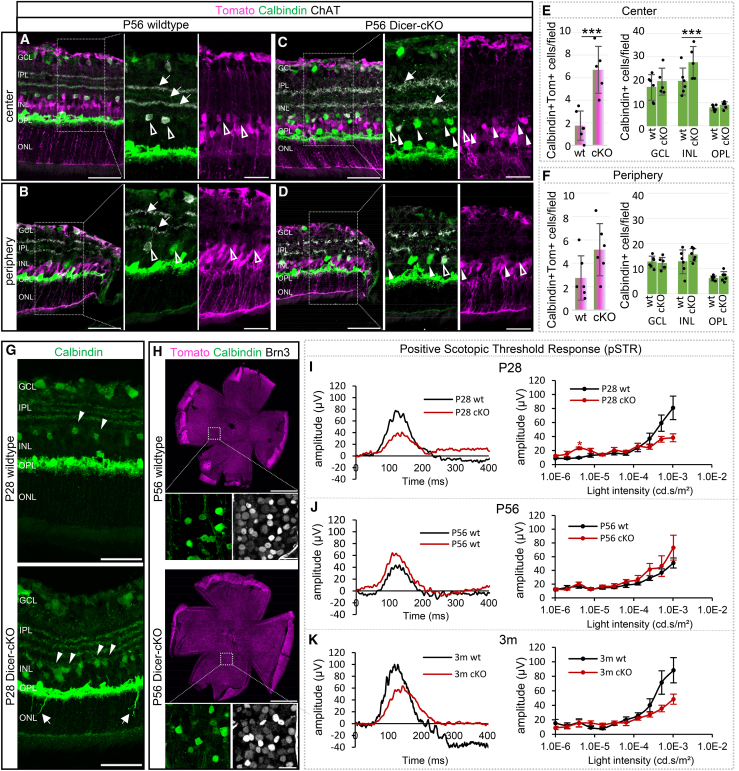
Dicer loss in late RPCs leads to an increased Calbindin+ amacrine cell population but does not affect ganglion cells (A–D) Antibody staining against Calbindin and choline acetyltransferase (ChAT) to label amacrine cells (ACs) in central or peripheral P56 wt or cKO retinas, with insets in higher magnification; arrows show stratification in IPL, white arrowheads Tomato+ ACs; unfilled arrowheads indicate Tomato-negative ACs. Markers depicted in white are labeled in black font. (E and F) Absolute numbers of Tomato+Calbindin+ cells per field in the INL and the overall number of Calbindin+ cells in the GCL, upper INL, and lower INL/OPL of central and the peripheral retinas; wt: *n* = 6, cKO: *n* = 6, mean ± S.D. (G) Calbindin labeling of P28 wt (*n* = 4) and cKO (*n* = 4) retinal cross sections showing ectopic horizontal cell neurites (arrows) in regions with high amacrine cell density (arrowheads) in cKO retinas. (H) Calbindin and Brn3 labeling of retinal flatmounts to visualize ACs and ganglion cells in the GCL of P56 wt and cKO mice, with insets showing higher magnifications. (I–K) Selected wave forms and intensity graphs of positive scotopic threshold responses (pSTRs) of full-field scotopic electroretinogram recordings of P28 (I, wt: *n* = 9, cKO: *n* = 10), P56 (J, wt: *n* = 10, cKO: *n* = 6), and 3-month-old wt and cKO mice (K,: *n* = 5 cKO: *n* = 5), mean ± S.E.M. See additional data in Figure S6. Significant differences are indicated, Mann-Whitney-U-test: ∗: *p* ≤ 0.05; ∗∗: *p* ≤ 0.01; ∗∗∗: *p* ≤ 0.001. Scale bars in A–D, insets: 25 μm, scale bars in G: 50 μm, scale bars in flat-mounted retinas in H: 1 mm, GCL insets: 25 μm. Layer description: see Figure 1.

Since we found ectopic Calbindin+ cells and increased transcripts in the P7 cKO retina (Figures 1I and 7K), we next evaluated this population in P56 retinas. In the P56 wild type, Calbindin+ ACs are located in the upper INL, with about 40% of them expressing choline acetyltransferase (ChAT, Figures 8A and 8B ). Calbindin labeling also beautifully visualizes the synaptic interactions in the IPL, which appear as three distinct synapse lines in the central retina (arrows). ChAT+ cells were found in the upper INL and GCL with synaptic connections in the IPL, overlaying the first and third Calbindin synapse layer. In the P56 cKO retinas, similar to the observations made at P7, many Calbindin+ cells were found in the upper INL. These cells were partly Tomato+ but mostly ChAT-negative (Figures 8C and 8D, Tomato+ arrowheads, Tomato-negative unfilled arrowheads). The quantification of the number of Tomato+Calbindin+ cells showed that numbers were doubled/tripled in the central cKO retina, compared to the wild type (Figures 8E and 8F). This led to an overall increase in the Calbindin+ AC population in the upper INL in the cKOs. Calbindin+ ACs, however, constitute only a minority of the overall population (∼20–30%). In the periphery, however, no AC increase was observed; yet, a trend toward increased Tomato+Calbindin+ ACs and overall Calbindin+ ACs was noted (Figures 8E and 8F). Nevertheless, the increased presumptive AC population seen at P7 was still present at P28 (Figure 8G arrowheads) and remained until P56. The rather loose-appearing processes in the IPL seen at P7 were still present at P28 and P56 and were also ChAT+ (Figures 8C, 8D, and 8G), which, however, did not result in significant functional AC impairments (OPs, Figures 7G–7I).

### Horizontal cells and retinal ganglion cells are not directly affected by miRNA loss in late RPCs

Because Calbindin is also expressed in RGCs and displaced ACs in the GCL, as well as HCs in the lower INL, adjacent to the OPL, we included these cell types in our analysis. RGCs and HC are early-born cell types that should not be affected by Dicer loss in postnatal RPCs/precursors. Indeed, HCs and RGCs were reporter-negative in both conditions, and cell numbers were similar in both conditions (Calbindin+ cell number counts in GCL and OPL are given in Figures 8E and 8F). However, although HCs were reporter-negative, they exhibited some morphological abnormalities, specifically ectopic neurites extending into the ONL (Figure 8G, arrows). This was particularly evident in areas with a higher AC density, suggesting some neuronal remodeling. A similar phenotype was reported in the *Chx10Cre*: *Dicer-cKO*, which, however, had its onset at embryonic stage.^34^ On a different note, some of these ectopic Calbindin+ ACs had cell bodies that somewhat resembled the typical MG somata (Figure 8G).

We next examined RGC pattern and density in retinal flatmounts and used antibodies against Calbindin in combination with antibodies against Brn3 (Figure 8H). We did not detect any density difference in all 4 quadrants in the wild type and Dicer-cKO, confirming our observation in cross sections. We concluded that RGCs were not affected (Brn3: wt 105 ± 2 cells/field vs. cKO: 104 ± 3 cells/field, Calbindin: wt: ∼13 ± 4 cells/field vs. cKO: 12 ± 1 cells/field, wt *n* = 3, cKO *n* = 3).

To measure RGC function, the positive scotopic threshold response (pSTR) was evaluated in P28, P56, and 3-month-old wild types and Dicer-cKO_RPC_ mice. The pSTR reflects activity in RGCs and a subpopulation of ACs.^87^ At all ages analyzed, we could not detect any significant differences between wild type and cKO pSTRs (Figures 8I–8K and S6D, *p* values for highest intensities obtained: P28 wt vs. cKO: *p* = 0.065; P56 wt vs. cKO: *p* = 0.875, 3m wt vs. cKO: *p* = 0.095). There was, however, a trend toward reduced amplitudes in P28 and 3-month-old cKOs, which is likely a result of the already decreased input (a- and b-wave). The slightly improved waveforms at P56 are unexpected and interesting. This could indicate a potential compensatory mechanism in the cKO at this stage and should be further analyzed in subsequent experiments.

### Possible miRNA regulators of the Dicer-cKO_RPC_ phenotype

**Figure 9:**
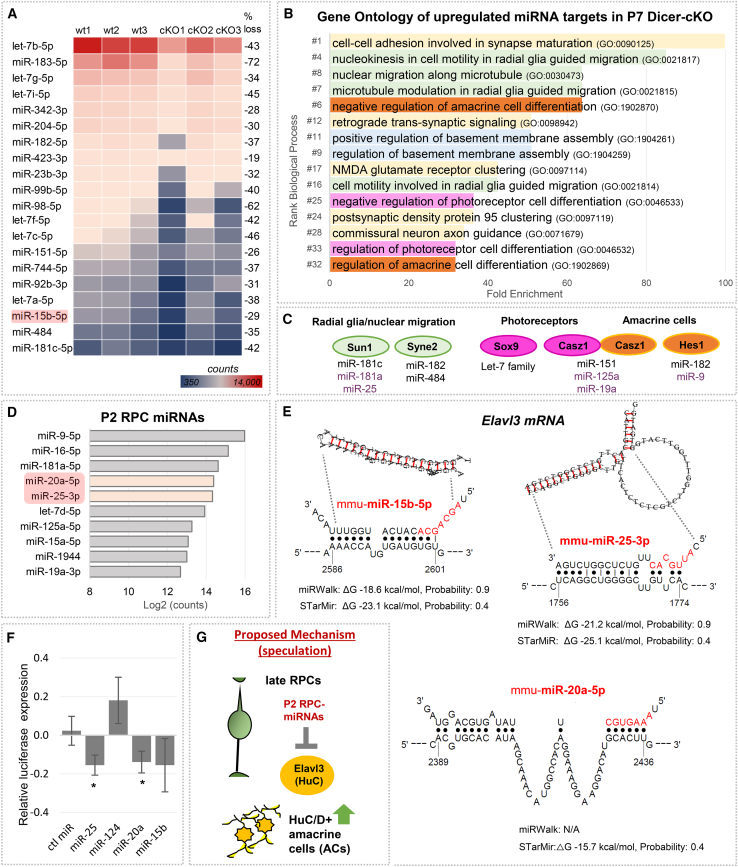
Processes regulated by RPC miRNAs include amacrine cell genes (A) Heatmap of the top 20 miRNAs highly expressed in FACS-purified P7 wild type progenies and their expression levels in the cKO, with emphasis on miR-15b (small RNA-seq, *n* = 3 per condition); see also Table S2. (B) Biological processes (gene ontology, GO, fold enrichment) of genes targeted by RPC-miRNAs found upregulated in P7 cKO FACS-purified progenies (>20%, bulk RNA-seq dataset, see also Table S3). (C) Selected genes associated with major GO biological processes and their targeting P7 and P2 (purple) RPC miRNAs, see also Table S1. (D) miRNAs highly expressed in P2 RPCs (log2 counts from Wohl et al.^101^), not found in the P7 miRNA dataset with emphasis on miR-20a/miR-25, see also Table S4. (E) Predicted binding sites for miR-15b-5p, miR-25-3p, and miR-20a-5p in the 3′UTR of the *Elavl3* mRNA using STarMir and miRWalk. miRNA seed sequences are indicated in red. Guide values for ΔG (hybrid stability): <−15 kcal/mol, probability: ≥0.5 indicate high hybrid stability/probability. Additional data shown in Figure S10 and Table S5. (F) Normalized relative luciferase activity of *Elavl3* 3′UTR reporter plasmids co-transfected with control miRNAs (ctl miR) or RPC mimics, 3 technical replicates, 6 independent experiments, mean ± S.D., Mann-Whitney U-Test: ∗: *p* < 0.05. (G) Schematic of proposed possible mechanism for AC overproduction: loss of late RPC-miRNAs miR-20a/25 might increase *Elavl3* mRNA, leading to ectopic HuC+ ACs.

We next aimed to identify possible miRNA candidates that might be involved in the observed Dicer-cKO phenotype. We performed and evaluated the data of small RNA-seq, which includes miRNAs, of FACS-purified P7 wild type and Dicer-cKO_RPC_ retinas. Dicer deletion results in interrupting the miRNA maturation of most, not all, miRNAs.^88^^,^^89^^,^^90^ Out of a total of 1200 miRNAs found in our dataset, 285 (∼24%) had detectable expression levels in the P7 wild type. 216 miRNAs out of these 285 miRNAs (∼76%) were downregulated in the cKO (up to 72% reduction), confirming successful Dicer deletion and impairment of miRNA biogenesis. The top 20 P7 wild type miRNAs and their expression levels in the cKO are shown in a heatmap in Figure 9A and are listed in Table S2. Among these top miRNAs were the let-7 family (with let-7b having the highest expression levels), as well as miR-183 and miR-204. We next used the Diana Tool microT to identify predicted targets of the top 20 miRNAs. We found about 12,660 hits with an interaction score of 0.7–1. We then compared this list of predicted targets of the top 20 downregulated miRNAs in the P7 cKO with the list of upregulated genes in the P7 cKO (at least 20% increase). We found 164 matches. Gene ontology (https://pantherdb.org) identified the biological processes in which these genes are involved (Figure 9B; Table S3). Major processes included synapse formation, signaling, and axon guidance, which might explain some of the observed differentiation deficits of differentiating neurons. Nucleokinesis and radial glia migration/cell motility were also prominent biological processes and a possible explanation for the displaced Sox2+/Sox9+ cells in the cKO. Processes related to photoreceptor and amacrine cell differentiation were also identified, which may be the reason for the robust rod cell status in the cKO and the ectopic AC population we observed. The genes associated with these major processes and their targeting miRNAs were *Sun1* (Sad1 And UNC84 domain containing 1) and *Syne2* (spectrin repeat containing nuclear envelope protein 2), involved in glial/nuclear migration. *Sun1* was found to be upregulated in the P7 cKO bulk RNA by 1.2-fold/23% (Table S1). Both genes are regulated by miR-181c/182/484 (Figure 9C; Table S1). *Sox9* (1.6-fold/55% increase in cKO) and *Casz1* (Castor zinc finger 1, 1.2-fold/24% increase in cKO) were associated with photoreceptor differentiation and are targeted by the let-7 family and miR-151, respectively. *Hes1* (1.4-fold/41% increase in cKO) and *Casz1* play a role in ACs differentiation. *Hes5* (1.3-fold/28% increase in cKO) is regulated by miR-182 (Figure 9C; Table S1). Notably, *Casz1*, which appeared in two different processes, is a well-studied gene. It not only regulates photoreceptor differentiation but also maintains late RPC identity.^91^^,^^92^^,^^93^ *Sox9* and *Hes1* are key factors in embryonic and postnatal brain/retinal development, as well as essential for glial development.^94^^,^^95^^,^^96^^,^^97^^,^^98^^,^^99^^,^^100^

A somewhat unexpected finding was that many miRNAs known to be expressed by RPCs/PC, including miR-9, miR-16, miR-25, miR-20a, miR-15a, and miR-19a^21^^,^^51^^,^^102^^,^^103^ were not detectable in our P7 dataset (or only at very low levels, Table S4). It was reported several years ago that P0-P2 RPCs, including miR-16, 15a/b, and miR-25, decline rapidly within the first postnatal days.^103^ However, this fact could also be due to the different methodology used and/or RNA degradation, as our yields of captured miRNAs were relatively low (25%). To rule out the possibility of missing essential miRNA candidates that might play a role in the cKO phenotype, we plotted the top 10 miRNAs found in P2 RPCs (Figure 9D; Table S4). Interestingly, several of them also target *Sun1*, *Casz1*, and *Hes*2 (as shown in Figure 9C, in purple font), including miR-25. Interestingly, miR-25, together with miR-15 and other P2 RPC miRNAs, was shown to induce RPC features in primary MG when overexpressed.^101^ These RPCs subsequently adopt BC features, but not AC features. Since our RPC-miRNA loss leads to an almost opposite outcome, we hypothesized that these P2 miRNAs might be involved in the observed BC/AC phenotype.

One major AC gene that caught our attention was *Elavl3*, which encodes HuC. HuC, together with HuD, is only expressed in RGCs (which are not affected in our model) and ACs, but not in BCs, HCs, or MG. Furthermore, *Elavl3* was found to be upregulated in P7 cKO progenies (bulk and scRNA-Seq) and may be a contributing factor to the higher number of HuC/D+ ACs observed in the cKO. To explore the possibility that P2 RPC-miRNAs target *Elavl3* mRNA, we utilized MirWalk and STarMir, two comprehensive databases for mRNA:miRNA target prediction. These two tools, based on AGO-Clip data, provide extensive information about and visualize the binding sites (Figure 9E; Table S5). Diana Tools and TargetScan were also used, although they do not utilize the most current mRNA transcripts. We identified three P2 miRNAs, namely miR-15b-5p, miR-20a-5p, and miR-25-3p, which are predicted to target *Elavl3*. The predominant binding sites were at the 3′UTR, but also sites at the CDS (coding sequence, Figures 9E and S10; Table S5, ΔG should be less than −15 kcal/mol, and the probability for binding should be higher than 0.5 and close to 1^104^). It should be noted that the predicted binding sites were rather unusual miRNA target sites with only partial seed matches. Nevertheless, for an initial screening of potential interactions with any of the three identified miRNA candidates, we utilized dual luciferase assays. We cloned the full-length 3′UTR directly downstream of the firefly luciferase gene. Cloning the entire 3′ UTR, rather than just small fragments, is considered more biologically accurate. It allows for capturing the effect of multiple miRNAs, which was the purpose of this pilot assay. It, however, will not reveal whether an interaction is direct or indirect. Nonetheless, we transfected miR-25-3p, miR-20a-5p, miR-15-5p, as well as miR-124-3p as a reference miRNA,^62^ or control mimics, together with a dual luciferase plasmid containing the 3′UTR of *Elavl3* mRNA. We found that miR-25-3p and miR-20a-5p significantly reduced luciferase activity. miR-15b-5p showed a trend toward reduction (Figure 9F). This supports our hypothesis that the reduction/loss of these miRNAs around P2-P4 via Dicer loss could have contributed to the increase in their target *Elavl3* mRNA (Figure 9G).

## Discussion

In the past decades, a variety of studies showed that Dicer and proper miRNA biogenesis are required for interspecies developmental processes of many organs and systems, including the central nervous system.^35^^,^^105^^,^^106^^,^^107^^,^^108^^,^^109^^,^^110^^,^^111^^,^^112^ In the neural retina, miRNAs have been shown to play a fundamental role during the embryonic phase, regulating the competence of early RPCs and the fates of their progenies.^21^^,^^36^^,^^37^^,^^38^^,^^39^ Whether Dicer and miRNAs also play a role in postnatal retinogenesis has not been explored *in vivo* yet.

Here, we demonstrate that Dicer in postnatal RPCs/PCs is essential for the proper differentiation and maturation of all late-born progeny, ensuring a fully functional retina. Overall, we observed five primary outcomes in the adult retina: a reduction in BCs (1) and MG number (2), impaired rod maturation and function (3), the presence of an ectopic immature population (4), and an ectopic AC population (5). Although somewhat similar observations regarding BC, rods, MG, and immature cells have been reported after Dicer loss during embryonic phases, to our knowledge, AC overproduction has not been documented. We propose *Elavl3*, which encodes HuC, and miR-20a/25 as possible key players and speculate that their interaction may be a possible regulatory mechanism underlying this AC phenotype. We also present and discuss additional possible key players that may be involved in the observed maturation delays, as a potential starting point for subsequent analyses.

### The vital role of Dicer in embryonic and postnatal retinogenesis

Dicer and miRNAs are crucial during the embryonic stages of retinogenesis. The first study was conducted by the Strettoi and Harfe Labs,^34^ using the *Chx10-Cre* mouse.^113^ *Chx10*, also known as *Vsx2*, is induced early, around E11.5.^113^^,^^114^^,^^115^^,^^116^^,^^117^ Although this manipulation did not cause a severe phenotype due to mosaic expression of *Chx10-Cre*, which was not traceable, one notable abnormality was the temporary formation of rosettes at P16. These rosettes appeared to cause dysfunction in rod and cone photoreceptors and ongoing degeneration in adult mice (up to 5 months). Signs of retinal remodeling, such as neurite sprouting of HCs, were observed, similar to our findings. Using the inducible *Ascl1CreER* transgenic mouse,^13^^,^^40^ we induced Dicer loss after birth, specifically targeting postnatal RPCs/PCs. Unlike Damiani et al.,^34^ we identified only functional deficits in rod photoreceptors (not cones) in young adult mice. Cone dysfunction was only seen later on, indicating secondary impairment due to rod malfunction^81^ and/or MG dysfunction.^70^ We did not see rosettes; however, horizontal cell (HC) neurite sprouting suggests similar remodeling mechanisms are present in both embryonic and postnatal Dicer-cKO models.

A second RPC Dicer-cKO study was conducted at the embryonic stage (E10.5) using the *αPax6Cre*:*R26EYFP* mouse. This model allowed for a more detailed investigation of Dicer-cKO cells through lineage tracing.^38^ In this mouse, only peripheral RPCs lost Dicer, enabling extensive tissue analysis into adulthood. Unlike Damiani et al.,^34^ a notably severe and interesting phenotype was observed in the affected periphery: early-born RGCs and HCs were produced in abundance, whereas late-born cell types (rods, MG, and BCs) were not formed. Late RPC genes, including *Ascl1*, were downregulated, and cell proliferation was slightly reduced at E16 and P1.^38^ This contrasts with our findings in postnatal RPCs, where we observed an increase in *Ascl1* transcripts and cell proliferation. This suggests that the specific progenitor state at which Dicer is deleted is essential, as that particular cell state seems to be maintained or extended. Additionally, embryonic and postnatal RPCs express somewhat different sets of miRNAs,^103^ which likely regulate different genes (targets) and biological processes, including RPC state and competence.^21^

A subsequent embryonic Dicer-cKO study, conducted by the Ashery-Padan Lab,^39^ confirmed the overproduction of RGCs at the expense of late-born retinal cells (HCs, PRs, BCs, ACs, and MG), as seen by Georgi et al.^38^ Davis et al.^39^, focused on neuroepithelial cells, including those of the iris and ciliary body, and used different *Cre* lines, including the *αCre* that labels optic cup progenitors (E10/11). Interestingly, similar to our findings, an increase in proliferation markers (i.e., Ki67, PH3, and Ccnd1) and Sox2+ cells (a progenitor marker) was observed, resulting in columns of progenitor cells that persisted until P8.^39^ An ectopic progenitor population was also found within the ciliary margins.^39^ Although our ectopic immature population was not restricted to the periphery and the ciliary marginal zones in our Dicer-cKOs did not show any abnormalities, these outcomes are comparable. This may suggest shared mechanisms for maintaining cells in an immature state. Interestingly, Davies et al.^39^ reported a differentiation failure of ACs and PRs in the *αCre-*cKO at E18.5, just before birth. This was due to the absence of Syntaxin1 and altered expression of *Bhlhe22* and *Tfap2a*. Both genes are found in differentiating ACs.^118^^,^^119^^,^^120^ However, Foxn4, a marker of cycling AC and HC precursors,^121^ and Ptf1, a marker expressed in postmitotic AC precursors,^122^ were present.^39^ This led the authors to conclude that miRNAs are dispensable for AC type specification but essential for their differentiation.^39^ In our study, all later-born AC types were present, but their proportions were altered. We also observed additional, ectopic Calbindin+Pax6+HuC/D+ ACs, which persisted at least until P56. This suggests some differences related to the RPC stage at the time Dicer is deleted (continued discussion below).

A very severe phenotype, with most cells gone by P14, was reported by the Watanabe Lab using the *Dkk3-Cre* mouse.^36^ *Dkk3-Cre* is active during the embryonic stage (E10.5),^123^ but throughout the entire retina. As a result, massive RPC death during embryonic phases was found, leading to microphthalmia. GCL and IPL were not formed. However, specific differentiation markers were expressed, including the MG/astrocyte marker GS (scattered throughout the Dicer-cKO retina). This is interesting since *Dkk3* is known to be expressed in MG,^123^^,^^124^^,^^125^ hence one would expect them to be affected most. Since that was not the case, MG progenitors/precursors appear to be relatively resilient cell types and may be less affected by Dicer/miRNA loss than neuronal progenitors/precursors. This could partly explain why, also in our study, MG was formed. Nevertheless, Dicer loss in young MG leads to MG impairment, which, however, takes several months to become evident.^67^^,^^70^

Nonetheless, cells most affected in the *Dkk3-Cre: Dicer-cKO* were BCs (rod BCs), showing weak PKC expression both *in vivo* and *ex vivo* (after electroporating P0 retinas and maintaining them in culture for 12 days^36^). Since we too found impaired BCs with predominantly impaired PKC+ rod BCs, this suggests that BC progenitors/precursors are more susceptible to Dicer/miRNA loss. The P0 *Dkk3-Cre: Dicer-cKO* explants also displayed an unchanged rod number, consistent with our findings, a slight increase in MG number, and a ∼66% reduction in Islet+ cells (RGC/ACs). Impaired ACs, indicated by weak Pax6/HuC/D expression, were also present *in vivo.*^36^ Hence, ACs seem to be affected differently in the *Dkk3-Cre* study. A possible explanation could involve the *Cre* lines used. Although *Dkk3* and *Ascl1* are both expressed in RPCs, some MGs, and mature ACs,^126^ their expression patterns do not appear to overlap. This is likely due to their opposing roles, as Ascl1 inhibits Dkk3, as demonstrated during fish regeneration.^127^ As a result, different subpopulations of RPC/precursors may have been manipulated in the Iida et al.^36^ study and our study. Additionally, the Dicer phenotype can be accelerated *ex vivo*^67^; therefore, a culture experiment does not necessarily reflect the same timeline observed *in vivo*.

### RPC miRNAs and RPC state—Peripheral versus central retinal impairments

One of the other significant findings in our study was a delay in tissue maturation. This was very likely due to increased and/or sustained transcripts associated with the cell cycle (more Ki67+ PH3+ cells in cKOs). A key miRNA regulator that plays a fundamental role during development by controlling cell cycle dynamics is let-7. let-7 (lethal-7) is a family of miRNAs consisting of let-7a, b, c, d, e, f, g, and i, which are expressed in mouse RPCs, MG, and neurons.^21^^,^^51^ During retinal development, let-7 accumulates in RPCs, and its levels and activity oscillate, mediating cell cycle exit in a concentration-dependent manner.^20^ One well-known target of let-7 is Cyclin D1.^128^^,^^129^^,^^130^^,^^131^ The *Ccnd1* gene encodes Cyclin D1, a transcript we found upregulated in the cKO mouse. *Ccnd1* is a major regulator of cell division and is downregulated during cell cycle exit and retinal cell maturation.^132^^,^^133^^,^^134^ Alterations in *Ccnd1* have been shown to affect cell fates significantly, and as a consequence, the cellular composition of the retina.^135^^,^^136^ Besides regulating the RPC cell cycle, let-7 also appears to play a role in primary mouse MG proliferation events.^101^ Therefore, the decline of members of the let-7 family we found in our study might have contributed to the prolonged cell cycle events we observed. Furthermore, in addition to cell cycle control, let-7 also negatively regulates *Ascl1* expression. This occurs in RPCs^21^ as well as in fish MG after initiating a natural regeneration program in response to injury.^111^^,^^137^^,^^138^ An induction of *Ascl1* was also observed in the aforementioned primary MG study after let-7 inhibition.^101^ Since the P7 cKO-progenies in our study displayed increased *Ascl1* transcripts, the loss of let-7 might be responsible for this outcome as well.

Because the retina matures and differentiates from the center to the periphery, we conducted thorough analyses of both central and peripheral regions of the adult retina. Compared to findings from embryonic studies,^36^^,^^38^ our results were somewhat unexpected, such as the still relatively normal size of the overall retina, which meant no drastic cell loss of the affected cells; a normal OCT, even though this was misleading since the cellular composition differed, and ectopic immature and AC populations in the central retina. Moreover, despite being impaired, the retina was still functional up to 4 months of age. The slow degeneration is an intriguing outcome that will require further investigation, and can be discussed in this study only to a limited extent. Part of the explanation might, however, lie in the cell state of the affected cells.

Furthermore, although most Tomato+ progenies were identified as proliferating RPCs, we also affected more committed precursor cells, especially in the central retina.^12^^,^^44^^,^^97^^,^^114^^,^^139^^,^^140^ As a result, these BC, rod, MG, and AC precursor cells may respond differently to Dicer/miRNA loss than late RPCs, leading to even more diverse outcomes. It appears that the impact of Dicer/miRNA loss on RPCs is faster than in more mature cells. This is probably because RPC miRNAs fluctuate and can change within a few days.^103^ Hence, their rapid turnover might enhance the Dicer-cKO phenotype. Conversely, miRNAs of specific differentiating cells appear relatively stable, at least under normal (injury-free) conditions. This may allow survival for several months, even if most cells are affected.^67^^,^^75^^,^^141^^,^^142^ The stability/half-lives of Dicer (both protein and mRNA), as well as miRNA, are also influential. This means that, although Dicer was impaired by P4, the mRNA and protein produced up to that point remained present and functional. The half-lives of miRNAs vary, ranging from hours to weeks, especially when bound to other factors like argonaut proteins.^143^^,^^144^^,^^145^^,^^146^^,^^147^ Consequently, not only the miRNA itself but also its half-life can be cell-type specific.^147^ As a result, the dynamics of miRNA may differ across retinal cell types, leading to distinct outcomes. Moreover, there is a Dicer-independent miRNA pathway.^88^^,^^148^ Although only a small number of miRNAs are processed this way, these miRNAs could still influence the canonical pathway.

Another aspect we want to address is the immature Sox9+Sox2+GS-negative population found in both the peripheral and central cKO retina. While the peripheral cells might indeed be remaining RPCs that stay undifferentiated, the central cells could belong to a different population. Although we observed these cells at P7 and P14, they may already have been more committed MG precursor cells, possibly even young glia, by the time the miRNA loss occurred. Furthermore, some of the ectopic ACs seen in the central retina could even be cells that originated from impacted MG precursors. This is because some of their nuclei were somewhat triangular and MG-like rather than round. Furthermore, Dicer/miRNA loss in P11 MG leads to proliferation and migration of the MG, which also lose GS expression over time.^67^ This could suggest a similar mechanism. The impairment of MG precursor/young glia could also be part of the events resulting in the relatively moderate degeneration we observed. It is conceivable that the glial reactivity of young glia in response to cell loss leads to less secondary neuronal loss.

### miRNAs of postnatal RPCs as possible regulators of amacrine cell populations

As mentioned earlier, another significant outcome of our postnatal RPC/PC study was the ectopic AC population, a result not previously reported. Since this is a new outcome, we decided to conduct an initial investigation on what potential regulators may have caused this phenotype. It is more straightforward to analyze first the upregulated genes in a Dicer-cKO and identify potential direct miRNA targets. Another reason for focusing first on ACs was the relatively slow degeneration we observed in the adult retina and the absence of functional defects of ACs. This may suggest a possible compensatory mechanism via these additional neurons (neuroplasticity).

So, what is causing the generation of these additional ACs? We do not have a complete answer to that question yet, and it is very likely a complex event involving many factors and miRNAs. For instance, early factors such as *Pax6*, *Sox2*, *Meis2*, and *Cdkn1c* (which encodes p51kip2), all found to be upregulated in P7 cKO progenies, are known to influence the competence of retinal progenitors and significantly affect the development of ACs from the early progenitor pool.^57^^,^^58^^,^^59^^,^^60^^,^^61^^,^^83^^,^^149^^,^^150^^,^^151^ In particular, Pax6 and Meis2 are required for the generation of late-born glycinergic AC types.^57^^,^^149^^,^^152^^,^^153^ Meis2 is also an early marker for postmitotic GABAergic ACs,^57^ which is targeted by miR-204.^150^ miR-204 was found in the P2 and our P7 progeny population, with declining levels in the cKO. Hence, the increase of ACs might also be partially regulated via the miR-204 and *Meis2*.^150^

Sox2, expressed in cholinergic and GABAergic subsets of ACs in the inner INL of the adult retina,^154^ also influences AC fates. Overexpression of Sox2 causes progenitor cells to shift toward an AC fate,^154^ while its knockdown results in a switch from amacrine to bipolar fates.^154^ Since we observed an increase in Sox2 and ACs, but a loss of BCs, Sox2 may act as an upstream regulator promoting AC overproduction at the expense of BC development. p57Kip2 was shown to regulate AC proportions and appears involved in determining GABAergic AC fates. Loss of p57Kip2 led to an increase in Calbindin+ ACs, although this p57Kip2-expressing AC population does not colocalize with markers like Calbindin, Calretinin, ChAT, or others,^61^ suggesting an indirect regulation. However, since we found that the transcripts of these genes were increased in our P7 cKO retinas (bulk RNA-seq data), they may contribute to the observed phenotype and may be regulated by RPC miRNAs.

Nevertheless, we would like to propose another possible mechanism, introducing new potential key players: miR-20a/25/15 and *Elavl3*. This remains speculative at this point, but it also provides a starting point for further in-depth analysis. *Elavl3* encodes HuC and was found to be expressed across all AC populations and upregulated in cKO progenies. Furthermore, the ectopic AC population in the P56 retina was HuC/D+. HuC/D is exclusively expressed in ACs and RGCs, but not in HCs, BCs, or MG. Because of that, and since RGCs are unaffected in our model, this marker serves as a relatively specific AC marker in our study. Additionally, *Elavl3* remains a relatively unexplored gene, despite initial studies being conducted over two decades ago. It is known that the Hu family of proteins contains RNA-binding proteins related to the Drosophila protein ELAV (embryonic lethal abnormal visual system) and are essential for nervous system development and maintenance (see reviews by Soller and White as well as Perrone-Bizzozero and Bolognami^155^^,^^156^).

There are four Hu family members in vertebrates with HuB, HuC, and HuD being neuron-specific, encoded by *Elavl2*, *Elavl3*, and *Elavl4*, respectively.^86^ These factors are first expressed in the GCL and later in the inner part of the INL, which contains ACs.^84^^,^^85^ *Elavl* genes play a vital role in neuronal differentiation, maintenance, and axogenesis in the brain.^64^^,^^66^^,^^157^ Recently, *Elavl* genes have been reported to be involved in AC subtype differentiation during retinogenesis to maintain normal retinal function.^63^^,^^65^ Therefore, *Elavl3* might be involved in AC fate, as well as overall tissue maturation processes. Two of the predicted miRNAs, namely miR-20a and miR-25, interacted with *Elavl3 3′UTR* (luciferase assays). However, whether that interaction is direct or indirect requires further and more detailed investigation. Since we cloned the full-length 3′UTR directly downstream of the firefly luciferase gene, it should be noted that this approach also bears the risk of indirect effects. The tested miRNA(s) might affect the expression of another factor that, in turn, influences the luciferase reporter. Therefore, further validation of the individual miRNA-specific binding sites, for instance by mutating them, would be required. Moreover, the luciferase assay is an artificial system that not only operates in an unrelated cell line, but it also separates the 3′ UTR from its natural coding sequence and places it in a non-native context. This can result in findings that don’t perfectly reflect *in vivo* gene regulation, which might also be species-specific. Murine primary cultures or explants in subsequent experiments might be useful tools to confirm our hypothesis and speculation.

Nevertheless, these miRNAs are known to be expressed in P2-4 RPCs^101^^,^^103^ and might be regulators since the overexpression of these RPC-miRNAs (in cocktails or separately) in young primary MG predominantly drives BC fates, but not primarily AC fates.^101^ ACs are, however, the other primary cell type usually generated in MG reprogramming experiments.^158^^,^^159^^,^^160^ This led us to hypothesize that these miRNAs might influence BC/AC fate determination. Although *Elavl3* hasn’t been examined in these reprogramming studies, it could be a direct target of miR-20a and miR-25. This regulation is thereby not limited to the *3′UTR*. We also found binding sites for these miRNAs in the coding sequence (CDS), which would result in the inhibition of *Elavl3* translation.^161^^,^^162^ Additionally, it is important to note that there are multiple binding sites for miR-25-3p. One appears to be a target-directed miRNA degrading site (TDMD site), leading to the degradation of the miRNA itself.^163^ Therefore, other regulatory events might also occur. To better understand these complex interactions, further experiments will be necessary.

Taken together, late RPC Dicer/miRNAs seem to regulate processes essential for the proper differentiation and maturation of all three major late-born cell types: rods, BCs, and MG. The loss of miRNA primarily affected rod BCs; therefore, BC progenitors and precursors appear to be the most vulnerable, regardless of whether Dicer loss occurred in late RPC/PCs (this study) or early RPCs, as shown in previous studies. In contrast, rod and MG progenitors and precursors appear to be quite resilient. AC progenitors and precursors, however, show opposing reactions depending on the timing of Dicer/miRNA loss, which seems beneficial in the postnatal retina. We speculate that a potential partial explanation for the observed AC phenotype might involve *Elavl3*, which encodes HuC. *Elavl3* appears to be regulated by miR-25/20a, at least in part, directly or indirectly. Overall, Dicer and mature miRNAs play a vital role in maintaining and ensuring the survival of not only embryonic but also postnatal RPCs and their progeny in the adult retina.

### Limitations of the study

This study presents the first comprehensive *in vivo* report on the structure and function of the adult retina after Dicer/miRNA depletion in postnatal RPC/PCs. The primary objective was to assess the overall retinal health with emphasis on cellular composition and function in adult mice, for which comprehensive histological analysis and cell quantifications were performed. Transcriptomic analyses, i.e., bulk RNA-, scRNA-, and small RNA-seq were conducted at P7 to confirm successful Dicer deletion. We chose this time point intentionally, primarily to confirm the early onset of the observed phenotypes. scRNA-seq served also as a primary tool to visualize and characterize the major cell populations as well as their subpopulations in P7 wild type and cKO retinas. Histological validation of important markers was conducted to confirm the presence of the protein and to show the location of the cells expressing these markers (protein). An in-depth histological quantification and cellular characterization at early postnatal stages was, however, not the aim of this study, as the primary objective is the adult retina. Additional transcriptomics at later stages is also beyond the scope of this study. Moreover, we used P7 transcriptomics data to identify potential key players, since they manifest early on. Several potential regulators were identified as a result of the aim of the study, but they were not the primary objective. They represent a possible starting point for subsequent in-death analysis. The thorough validation of these identified putative regulators requires tissue culture experiments, which cannot be covered in this study. Lastly, small RNA-seq currently cannot be performed at single-cell resolution, so these data reflects a heterogeneous cell population with an unknown cellular composition.

## Resource availability

### Lead contact

Further information and requests for resources and reagents should be directed to and will be fulfilled by the lead contact, Stefanie Gabriele Wohl, (swohl@sunyopt.edu).

### Materials availability

Mouse lines generated in this study can be obtained after obtaining permission from the vendor selling the original strains and after completing a materials transfer agreement. This study did not generate new, unique reagents. Plasmids generated in this study will be made available on request, but we may require a completed materials transfer agreement.

### Data and code availability

All relevant data and resources can be found within the article and its supplementary information. scRNA-seq as well as bulk RNA (long RNA) and miRNA (short RNA) data files generated in this study were deposited at Gene Expression Omnibus (GEO), accession number GSE288381 and are publicly available. The late RPC miRNAs dataset (Nanostring dataset from^101^ is also available at GEO (GSE135835, under SuperSeries accession number GSE135846).

## Acknowledgments

The authors would like to thank Dr. Adriana Heyguy and her outstanding team from the NYU Langone Health Genome Technology Center for handling our miRNA-seq and bulk RNA-seq samples, as well as for their exceptional support. We thank Dr. Aristotelis Tsirigos from the NYU Applied Bioinformatics Laboratories (ABL) for his support. We thank Autumn Cholger from FluentBio/Illumina for excellent training and in-depth troubleshooting. We thank Prof. Dr. Volker Busskamp and his team from the University of Bonn for providing protocols, reagents, and advice for the Luciferase assays. We thank Dr. Joseph A. Brzezinski IV from the University of Colorado for sharing the EdU injection protocol. We thank Dr. Ching-Hwa Sun from Weill Cornell Medicine for sharing HEK 293T cells. We also thank Dr. Miruna Ghinia-Tegla for suggesting the Ribeye antibody. This study was funded by the 10.13039/100000053National Eye Institute (NEI, R01 EY032532) to S.G.W., The New York State Empire Innovation Grant to S.G.W., 10.13039/100007258SUNY Start up to S.G.W., 10.13039/501100001655DAAD stipend to E.B. (2022-2023), Förderverein Augenoptik/Optometrie der Berliner Hochschule für Technik e.V.—stipend to K.H. and E.B., 10.13039/100000053NEI T35 2T35 EY020481 to J.J., and SUNY Graduate Assistantship to D.L., S.K., and S.C., as well as 10.13039/100000053NEI, R01 EY035381 to B.S.C. and a Career Development Award to B.S.C. and unrestricted funds to the WashU Department of Ophthalmology and Visual Sciences from Research to 10.13039/100010264Prevent Blindness.

## Author contributions

Conceptualization: S.G.W., B.S.C., and A.K.-J.; data curation: S.K., D.L., E.B., K.H., A.K.-J., C.S., M.L.C., M.A., K.B., S.C., J.J., B.S.C., and S.G.W.; formal analysis: S.K., D.L., E.B., K.H., A.K.-J., C.S., M.L.C., K.B., B.S.C., and S.G.W.; funding acquisition: S.G.W., B.S.C., E.B., K.H., S.K., D.L., S.C., and J.J.; investigation: S.K., D.L., E.B., K.H., A.K.-J., C.S., M.L.C., M.A., K.B., S.C., J.J., B.S.C., and S.G.W.; methodology: S.G.W., B.S.C., A.K.-J., C.S., S.V., and K.B.; project administration: S.G.W.; resources: S.G.W. and S.V.; supervision: S.G.W. and S.V.; validation: S.K., D.L., E.B., K.H., A.K.-J., C.S., M.L.C., K.B., B.S.C., and S.G.W.; visualization: S.K., D.L., A.K.-J., C.S., M.A., K.B., B.S.C., and S.G.W.; writing – original draft: S.K., D.L., E.B., K.H., A.K.-J., M.A., K.B., B.S.C., and S.G.W.; writing – review and editing: S.G.W., B.S.C., and A.K.-J.

## Declaration of interests

The authors declare no competing interests.

## STAR★Methods

### Key resources table

**Table:** 

REAGENT or RESOURCE	SOURCE	IDENTIFIER
**Antibodies**		

Rat α-RFP	Antibodies online	Cat# ABIN334653; RRID: AB_10795839
Mouse α-Brn3a	Millipore	Cat# MAB1585; RRID: AB_94166
Mouse α-glutamine synthetase	Millipore	Cat# MAB302; RRID: AB_2110656
Rabbit α-Sox9	Millipore	Cat# AB5535; RRID: AB_2239761
Goat α-Sox2	R&D Systems	Cat# AF2018; RRID: AB_355110
Mouse α-PKC	Millipore	Cat# P5704; RRID: AB_477375
Rabbit α-Pax-6	Invitrogen	Cat# 42–6600; RRID: AB_2533534
Rabbit α-Ribeye	SYSY	Cat# 192103; RRID: AB_2086775
Rabbit α-HuC/D	Fisher Scientific	Cat# A-21271; RRID: AB_221448
Goat α-Otx2	R&D System	Cat# AF1979; RRID: AB_2157172
Mouse α-Calbindin	Millipore	Cat# ABN 2192; RRID: AB_2935805
Goat α-ChAT (Choline Acetyltransferase)	Millipore	Cat# AB144P; RRID: AB_2079751
Rabbit α-ZO-1	Invitrogen	Cat# 61–7300; RRID: AB_2533938
Rat α-PH-3	Novus	Cat# NB600-1168; RRID:AB_10002855
Rabbit α-Ki67	Abcam	Cat# ab15580; RRID: AB_443209
Rabbit α-*M*-opsin	Sigma	Cat# AB5405; RRID:AB_177456
Peanut glutamine (PNA)	VectorLabs	Cat# FL-1071; RRID:AB_2315097
Rhodamine Red 570 Donkey Anti-Rat IgG (H + L)	Jackson ImmunoResearch Laboratories, Inc.	Cat# 712-296-150; RRID: AB_2340677
Alexa Fluor 488 Donkey Anti-Mouse IgG (H + L)	Jackson ImmunoResearch Laboratories, Inc.	Cat# 715-546-150; RRID: AB_2340849
Alexa Fluor 488 Donkey Anti-Rabbit IgG (H + L)	Jackson ImmunoResearch Laboratories, Inc.	Cat# 711-546-152; RRID: AB_2340619
Alexa Fluor 488 Donkey Anti-Goat IgG (H + L)	Jackson ImmunoResearch Laboratories, Inc.	Cat# 705-546-147; RRID: AB_2340430
Alexa Fluor 647 Donkey Anti-Rabbit IgG (H + L)	Jackson ImmunoResearch Laboratories, Inc.	Cat# 711-606-152; RRID: AB_2340625
Alexa Fluor 647 Donkey Anti-Goat IgG (H + L)	Jackson ImmunoResearch Laboratories, Inc.	Cat# 705-606-147; RRID: AB_2340438

**Chemicals, peptides, and recombinant proteins**		

Corn Oil	Aqua Solutions	Cat# 8001-30-7
4,6-diamidino-2-phenylindole (DAPI)	Sigma	Cat# D9542
Fetal Bovine Serum (FBS)	Corning	Cat# MT35010CV
Fluoromount G	SouthernBiotech	Cat# 0100-01
Hank’s balanced salt solution (HBSS) with Ca^2+^ and Mg^2+^	Corning	Cat# 21-023-CV
High-sensitivity DNA kit	Agilent	Cat# 5067-4626
**Normal Horse Serum**	**Jackson Immunoresearch Lab**	**Cat# 008-000-121**
Ketamine	Dechra Veterinary Products	Cat# 20040127
1% Methylcellulose	Allergan	Cat# 396264
Papain Dissociation System	Worthington Biochemical	Cat# LK003153
Paraformaldehyde (PFA)	Avantor	Cat# 100503-917
Phenol-free Neurobasal medium	Gibco	Cat# 12349-015
Phenylephrine hydrochloride 2.5%	Bausch and Lomb	Cat# 3M379
Phosphate buffered saline (PBS)	Fisher Scientific	Cat# BP399-4
Sucrose	Fisher Scientific	Cat# BP220-212
Tamoxifen	Cayman	Cat#13258
Triton X-100	Sigma	Cat# T8787-100 ML
1% Tropicamide	Sandoz	Cat# 120L4
Xylazine	Akorn	Cat# 081401A
mmu-miR-124-3p mimics	Horizon Dharmacon	Cat# C-310390-05-0020 MIMAT0000134
mmu-miR-15a-5p mimics	Horizon Dharmacon	Cat# C-310510-05-0020 MIMAT0000526
mmu-miR-20a-5p mimics	Horizon Dharmacon	Cat# C-310514-05-0002 MIMAT0000529
mmu-miR-25-3p mimics	Horizon Dharmacon	Cat# C-310564-05-0020 MIMAT0000652
miR-negative control	Horizon Dharmacon	Cat# CN-001000-01-50; RRID: MIMAT0000039

**Critical commercial assays**		

Dual-Glo Luciferase Assay System	Promega	Cat# E2920
miRNeasy Tissue/Cells Advanced Micro Kit	Qiagen	Cat# 217684

**Deposited data**		

P7 Ascl1 wildtype bulk mRNAseq fastq and count tables_	This paper	GEO GSE288381, GSM9156032-GSM9156035
P7 Ascl1 cKO bulk mRNAseq fastq and count tables_	This paper	GEO GSE288381, GSM9156036-GSM9156039
P7 Ascl1 wildtype small RNAseq fastq and count tables_	This paper	GEO GSE288381, GSM9156040-GSM9156042
P7 Ascl1 cKO smallRNAseq fastq and count tables_	This paper	GEO GSE288381 GSM9156043-GSM9156046
P7 Ascl1 wildtype scRNAseq fastq	This paper	GEO GSE288381, GSM9156046 + GSM9156048
P7 Ascl1 cKO scRNAseq fastq	This paper	GEO GSE288381, GSM9156047 + GSM9156049

**Experimental models: Cell lines**		

Hek293T	sample from Dr. Ching-Hwa Sung, Weill Cornell Medicine	RRID: CRL_3216, ATCC

**Experimental models: Organisms/strains**		

*Ascl1tm1.1(Cre/ERT2)Jejo/J*	Jackson Laboratory	RRID:IMSR_JAX:012882
*B6.Cg-Dicer1tm1Bdh/J*	Jackson Laboratory	RRID:IMSR_JAX:006366
*B6.Cg-Gt(ROSA)26Sortm14(CAG-tdTomato)Hze/J*	Jackson Laboratory	RRID:IMSR_JAX:007914
*129S1/SvImJ*	Jackson Laboratory	RRID:IMSR_JAX:002448

**Oligonucleotides**		

Genotyping primer Ascl1Cre wildtype Forward: TCC AAC GAC TTG AAC TCT ATG G Reverse: CCA GGA CTC AAT ACG CAG GG	Sigma Millipore	WD12369750 WD12369751
Genotyping primer Ascl1Cre mutant Forward: AAC TTT CCT CCG GGG CTC GTT TC Reverse: CGC CTG GCG ATC CCT GAA CAT G	Integrated DNA Technologies	427612814 427612813
Genotyping primer tdTomato wildtype Forward: AAG GGA GCT GCA GTG GAG TA Reverse: CCG AAA ATC TGT GGG AAG TC	Sigma Millipore	WD11246623 WD11246624
Genotyping primer tdTomato mutant Forward: CTG TTC CTG TAC GGC ATG G Reverse: GGC ATT AAA GCA GCG TAT CC	Sigma Millipore	WD11246621 WD11246622
Genotyping primer Dicer Forward: CCTGACAGTGACGGTCCAAAG Reverse: CATGACTCTTCAACTCAAACT	Sigma Millipore	WD12191088 WD12191089
Genotyping primer Dicer Deletion Forward: CCT GAC AGT GAC GGT CCA AAG Revers2: CCT GAG CAA GGC AAG TCA TTC	Sigma Millipore	WD12191088 WD12191090
*Elavl3* primers Forward: AATTCTAGTTGTTTAAACG AGCTCGGCATTTATTGCCCTCCCTCCC Reverse: CAGCTTGCATGCCTGCAGG TCGACTTTAAGATTCTGAACTTTTTA TT TTCTGGCA TG	Sigma Millipore	WD10998934 WD10998935

**Recombinant DNA**		

pmiRGLO empty	Kutsche et al.^164^	Cat# 78128; RRID:Addgene_78128
pmiRGLO *Elavl3 3′UTR*	This paper	N/A

**Software and algorithms**		

Adobe Bridge	Adobe, https://www.adobe.com/	Version 2022
Adobe Photoshop	Adobe, https://www.adobe.com/	RRID: SCR_014199; Version 2022
Affinity Photo 1.8.5	Serif Europe, https://affinity.serif.com/	RRID: SCR_016951
Diana-TarBase v.8	http://diana.imis.athena-innovation.gr/DianaTools/index.php?r=tarbase/index	RRID: SCR_010841
InVivoVue Diver Release_2.0	Bioptigen, https://bioptigen-invivovue-diver.software.informer.com/	Not registered
Sigma Plot 10.0	Systat Software Inc., https://grafiti.com/sigmaplot-detail/	Not registered
Fluoview FV31S	Olympus, http://mirwalk.umm.uni-heidelberg.de	Not registered
miRWalk	http://mirwalk.umm.uni-heidelberg.de/	RRID: SCR_016509
STarMir	http://sfold.wadsworth.org	Not registered
TarPmiR	https://hulab.ucf.edu/research/projects/miRNA/TarPmiR/	Not registered
TargetScan	https://www.targetscan.org/vert_80/	RRID: SCR_010845

**Other**		

Agilent 2100 Bioanalyzer System	Agilent	Cat# G2938C; RRID:SCR_018043
BD Melody sorter	BD BioScience	RRID:SCR_023209; https://www.bdbiosciences.com/en-us/products/instruments/flow-cytometers/research-cell-sorters/bd-facsmelody
Countess II	Life technologies	Cat# AMQAS1000R; https://www.thermofisher.com/order/catalog/product/AMQAX2000?SID=srch-srp-AMQAX2000
Confocal Laser scanning microscope	Olympus	Cat# FV1200; RRID:SCR_016264, Cat# FV3000 RRID:SCR_017015
Envisu SD-OCT R4300	Leica Systems	https://www.leica-microsystems.com/products/light-microscopes/p/envisu-r-class/
Espion Electrodiagnostic rodent system	Diagnosys LLC	https://www.diagnosysllc.com/preclinical/preclinical-products/
FilterMax F3 Multi-Mode Microplate Reader	Molecular Devices	https://www.moleculardevices.com/products/microplate-readers/multi-mode-readers/filtermax-f3-f5-readers
Keyence microscope	Keyence	Cat# BZ-X810; RRID:SCR_025160

### Experimental model and study participant details

#### *In vivo* animal studies

All mice (species *Mus musculus*) used in this study were housed at the State University of New York, College of Optometry, following the Institutional Animal Care and Use Committee-approved protocols (IACUC, SW2021-12-1, SW2024-12-1). The *Ascl1-creERT2* strain (ID 12882, generated by Dr. Jane Johnson,^40^ the *R26-stop-flox-CAG-tdTomato* strain (also known as Ai14, ID 007908), and the Dicer conditional knockout strain (*Dicer*^*f/f,*^ ID 006001,^43^ were obtained from the Jackson Laboratory (key resources table) Males and females were used, with experimental groups consisting typically of more than one litter. Littermates were randomly assigned to experimental groups, whenever possible. Cre-negative littermates, as well as S129 background wild type mice (key resources table), were used as reference controls. Time point of analysis included postnatal day (P) 4, 7, 14, 28, 56, as well as 3 and 4 months of age.

#### Cell lines

Human embryonic kidney cells (ATCC, authenticated, female origin, obtained from Dr. Ching-Hwa Sung, Cornell University) were grown and maintained in DMEM medium supplemented with 1% FBS (Fischer Scientific) and passaged when 70–80% confluency was reached, up to 30 passages.

### Method details

#### Generation of mouse strains

The *Ascl1-creERT2* strain (ID 12882, The Jackson Laboratory, key resources table, generated by Dr. Jane Johnson,^40^ was crossed with the *R26-stop-flox-CAG-tdTomato* strain (also known as Ai14, ID 007908, The Jackson Laboratory, key resources table) to create the *Ascl1CreER: stop*^*f/f*^*-tdTomato* strain, further referred to as wild type (wt). This wild type strain was then crossed with the Dicer conditional knockout strain (*Dicer*^*f/f,*^ ID 006001, The Jackson Laboratory, key resources table ^43^) to create the *Ascl1CreER: Dicer*^*f/f*^: *stop*^*f/f*^*-tdTomato,* further referred to as the Ascl-Dicer-cKO strain (cKO). The homozygous Dicer-cKO_RPC_ mouse will have a functionless Dicer enzyme in late RPCs due to the excision of exon 23 of the *Dicer1* gene via Cre recombinase.^43^ Heterozygous mice (Dicer^f/wt)^were not used in this study.

#### Cre induction

Genotyping was done using the primers listed in Table S6, key resources table. *Cre*-positive, not tamoxifen-treated, mice were checked for reporter induction. Successful deletion of exon 23 of *Dicer1* was confirmed via PCR for retinal lysates at P4, P7, and adult mice. Tamoxifen (Sigma, St. Louis, MO) was administered intraperitoneally at 75 mg/kg in corn oil for three consecutive days to initiate the recombination of the floxed alleles for inducing tdTomato at postnatal day (P) 1–3, P4-6, P9-11, or P13-15, or to initiate Dicer deletion at P1-3.

#### EdU labeling

Labeling of dividing cells using 5-Ethynyl-2′-deoxyuridine (EdU) was conducted at P3. EdU (Santa Cruz, cat. sc-284628) was reconstituted in sterile PBS, and 25 μL were injected subcutaneously into the back (skinfold) of the P3 old pups of unknown gender (10 mg/mL).^165^

#### Retinal spectral-domain optical coherence tomography (SD-OCT) imaging

*In vivo*, retinal structure and integrity were assessed using spectral-domain optical coherence tomography (SD-OCT) imaging using the Envisu R2200 SD-OCT device (Bioptigen, Durham, NC). Animals were anesthetized using 75–100 mg/kg Ketamine and 5–10 mg/kg Xylazine dissolved in sterile saline, injected intraperitoneally. Pupils were dilated with phenylephrine hydrochloride (2.5%) and tropicamide (0.5%). Methylcellulose was used to keep the corneas lubricated during the scans. Rectangular and radial volume scans were obtained while centered on the optic nerve head (1.4 × 1.4 mm, 1000 A-scans/B-scan X 15 frames/B-scan). For image analysis and measurements, retinal layers were manually segmented in a 9 × 9 plot in four regional quadrants (superior-inferior, nasal-temporal). Thicknesses were measured using DiverRelease_2_4 software. The total retinal thickness was defined as the distance between the inner border of the retinal nerve fiber layer (RNFL or NFL) and the outer border of the retinal pigment epithelium (RPE). All nuclear and plexiform layers were analyzed, including the nerve fiber layer/ganglion cell layer (NFL/GCL), inner plexiform layer (IPL), inner nuclear layer (INL), outer plexiform layer, and outer nuclear layer (ONL). The inner and outer segments, as well as the RPE, were measured individually. The distance between the outer border of the outer limiting membrane (OLM, also known as external limiting membrane, ELM) and the outer border of the RPE was examined as a whole, allowing for more accurate measurement of overall outer retinal thickness, which can be a valuable biomarker for various retinal conditions.^166^^,^^167^ Thickness measurements were conducted in the central areas, within a radius of approximately 650 μm from the optic nerve (ON).

#### Electroretinogram (ERG) recordings

For electroretinography, mice were dark-adapted overnight and anesthetized the following day via an intraperitoneal injection of 75–100 mg/kg Ketamine and 5–10 mg/kg Xylazine dissolved in sterile saline. Recordings were performed using the Espion Electrodiagnostic rodent system (Diagnosys LLC, Lowell, MA, USA) in a darkroom. Pupils were dilated with 1% tropicamide ophthalmic solution, and the eyes were lubricated using 1% methylcellulose. During the recording, mice were kept on a heat pad to maintain a constant body temperature. A gold wire electrode served as the recording electrode, while needles inserted into the cheek and back of the mouse served as reference and ground electrodes, respectively. A handheld, portable stimulator was used as the light source. Full-field 5-ms flashes were used to elicit responses. Dark-adapted ERGs were obtained with light intensities ranging from 0.001 to 64 cd s/m^2^. To assess photoreceptor functionality, scotopic and photopic ERG a- and b-wave amplitudes were measured from the baseline to the a-wave trough and from the a-wave trough to the b-wave peak, respectively. For an in-depth analysis of the scotopic b-wave, the Naka-Rushton equation was employed, where V(I) = Vmax (In)/(In + Kn), as shown in Equation 1. The intensity–response amplitudes were fitted using the generalized equation: “V” is the amplitude at stimulus intensity “I”. “Vmax” is the maximum/saturated amplitude and an indirect measurement of responsiveness. “K” is the semi-saturation constant representing the stimulus intensity I at which half of the saturated amplitude Vmax is reached. Hence, K is an indirect measurement of sensitivity. “n” is the slope of the fit curve and an indirect measurement of heterogeneity.^168^^,^^169^

(Equation 1) $$V\left(I\right)=\setminus frac\left\{V_{\left\{\max\right\}et}al\;\setminus cdot\;I^{ˆ}n\right\}\left\{I^{ˆ}n+K\_n\right\}$$

Generalized Naka-Rushton equation for estimating fit parameters.

To assess the function of the inner retinal layers, oscillatory potentials (OPs) and positive scotopic threshold responses were analyzed. OPs are low-amplitude wavelets superimposed on the ascending b-wave and are believed to be generated by the inhibitory feedback interactions between amacrine, bipolar, and ganglion cells.^170^^,^^171^^,^^172^^,^^173^ Total OP amplitude was determined by adding the amplitudes of each OP wavelet (OP1, OP2, OP3, and OP4). A single OP wavelet amplitude was calculated from the trough to the peak of that wavelet, as previously described.^174^ Scotopic threshold responses (STRs) were generated in the proximal retina and are believed to reflect the activity of retinal ganglion and amacrine cells.^175^^,^^176^ Positive scotopic threshold responses are waveforms with positive polarity, similar to the b-wave. Still, they are elicited at light levels too low to generate the b-wave produced by bipolar cells.^177^ The pSTR amplitude was evaluated from the pre-stimulus baseline to the peak of the positive deflection.

#### Tissue preparation for immunofluorescent labeling

Mice were euthanized, and eyes were marked at the nasal side, enucleated, and transferred to pre-chilled phosphate-buffered saline (PBS, Fisher Scientific, Hampton, NH, USA). For retinal cross sections, the eyeball was fixed in cold 4% paraformaldehyde (PFA, VWR International, Radnor, PA, USA, 4°C) for 20 min. The cornea, lens, iris, and vitreous body were removed (video available in,^178^ and eyecups were fixed for an additional 20 min in cold 4% PFA. After fixation, eyecups were subsequently washed for 10 min in pre-chilled PBS, and incubated with 30% sucrose (Fisher Scientific, Hampton, NH, USA) in PBS overnight at 4°C. The tissue was embedded in O.C.T. embedding medium (Sakura Finetek, Torrance, CA, USA) with nasal-temporal orientation and frozen at −80°C. The frozen tissue was cross-sectioned into 12 μm-thick sections for subsequent immunofluorescent labeling. For labeling, frozen sections were dehydrated at 37°C for 20 min, then fixed for 20 min with 4% PFA, and subsequently washed in PBS. Sections were incubated in blocking solution (10% horse serum, Fisher Scientific, Hampton, NH, USA, in PBS; with 0.5% Triton X-100, Sigma-Aldrich, St. Louis, MO, USA) for at least 1 h at RT, then incubated with primary antibodies (Tables S7 and S8) overnight. Secondary antibody incubation was done on the following day for 1 h at RT. All antibodies were diluted in 10% horse serum. 4,6-Diamidino-2-phenylindole (DAPI, Sigma-Aldrich, St. Louis, MO, USA, 1:2,000) was used for counterstaining. For EdU labeling, the Click-iT EdU Alexa Fluor 647 Imaging Kit (Catalog #C10340, Fisher Scientific) was used, following the manufacturer’s instructions. Slides were mounted with cover glasses and mounting medium (Invitrogen, Waltham, MA, USA). Only validated antibodies were used in this study. Validation was based on item information given by the vendor as well as publications.

For retinal flatmounts and RPE preparation, muscles and connective tissues on the sclera were removed from the eyeball to facilitate tissue flattening. The cornea, iris, lens, and vitreous body were removed, and the RPE (with attached retina) was carefully transferred onto filter paper. The tissue was cut radially (4 times), and the retina was gently detached and fixed with cold 4% PFA for at least 30 min up to 1 h at RT. The RPE (with sclera) was fixed with cold 4% PFA for 1–2 h at RT. After fixation, the tissue was washed three times in PBS at RT for 10 min and stained following the protocol as described above and the markers listed in Tables S7 and S8, and key resources table.

#### Microscopy and cell count

Retinal eye cups were imaged using a confocal laser scanning microscope (FM1-200 and FV3000, Olympus, Shinjuku City, Tokyo, Japan) and the Fluoview FV31S software for whole tissue imaging. Whole cup stitched images were taken with 10× and 20× objectives. Images of peripheral areas (close to the ora serrata) and central areas (within 500 μm distance from the optic nerve [ON]) were taken with 20× or 40× objectives as z-stacks. For confocal analysis, image parameters were defined by control/wild type groups and used (reloaded) for all conditions. Images were processed and analyzed using Adobe Bridge, Adobe Photoshop, and Affinity. Cells per field (field = full image) or photoreceptor segments per field (field = full image) were counted for at least four images per mouse per staining protocol from central and peripheral cross-sections. The number of rod columns was counted for 10 areas per image for at least four images of the central regions per mouse. For P56 analysis, at least six mice per group were evaluated per group and condition (power analysis 85%); the exact n for the particular cell counts is given in the respective legends. For color-blind friendly figures, either gray-scale or green/magenta images were chosen or generated using Photoshop. Several lab members analyzed images under blinded conditions.

### Fluorescence-activated cell sorting

P7 wild type and Dicer-cKO retinas were checked for successful recombination and reporter induction using a fluorescence microscope (Keyence BZ-X810, KEYENCE, Elmwood Park, NJ, USA). For each sort, the retinas of 1–3 reporter+ mice were pooled and dissociated in DNase/Papain (50 μL/500 μL respectively, Worthington Biomedical, Lakewood, NJ, USA) for 20 min at 37°C on the shaker (Thermomixer, Fisher Scientific, Hampton, NH, USA), triturated, mixed with Ovomucoid (500 μL, Worthington Biomedical, Lakewood, NJ, USA), and centrifuged for 10 min at 300 × g. The pellet was resuspended in 800 μL DNase/Ovomucoid/Neurobasal without phenol red medium (Gibco, Hampton, NH, USA) solution per retina (1:1:10, respectively). Cells were filtered through a 35-μm filter, sorted using a 100-micron nozzle (BD Melody, BD Bioscience, Franklin Lakes, NJ, USA), and collected into two chilled FBS-coated tubes containing Neurobasal medium without phenol red. Cells with the brightest fluorescence were considered “positives” (RPC-progeny fraction), and cells with no fluorescence (“negatives”) were considered as the non-progeny fraction. Debris was excluded. After collection, the Tomato+ fraction and the Tomato-negative fraction were post-sorted, and a fraction of each sort was plated in a 48-well plate to confirm and validate purity and viability using 7-AAD viability staining (BD Bioscience). After the sort, cells were spun for 10 min at 300 × g at 4 °C. Pellet was either resuspended in HBSS for scRNA-seq and cell numbers were ascertained using an automated cell counter (Countess, Thermo Fisher), or homogenized in RLT lysis buffer (miRNeasy Tissue/Cells Advanced Micro Kit, 217684, Qiagen, Hilden, Germany) for subsequent RNA extraction.

#### Single-cell RNA sequencing and data analysis

Single-cell RNA sequencing was performed using pre-templated instant partitions (PIP) sequencing (PIPseq,^179^). The T20 kit (≤20,000 cells output, FBS-SCR-T20-4-V4.05, Fluent Biosciences) was used according to the manufacturer’s instructions and split in half (2× “T10”). In brief, tubes containing template beads were thawed on ice, and the required number of input cells was added per tube (i.e., approximately 25,000–30,000 live cells per T10 sample. The suspension was mixed gently by pipetting and processed further according to the manufacturer’s instructions. After the cDNA synthesis, purification, and library preparation, the concentration, integrity, and quality were assessed using the Agilent 2100 Bioanalyzer System and the high-sensitivity DNA kit (Agilent Technologies, Santa Clara, CA, USA). Illumina-compatible libraries were sequenced using the NovaSeq X + Sequencing System (Illumina, 40,000 reads per cell) at the NYU Genome Technology Center. FASTQ files were preprocessed using PIP Seeker, a comprehensive analysis solution that performs barcode matching, quality control, mapping with the STAR aligner40, deduplication, transcript counting, cell calling, clustering, differential expression, and cell type annotation. Resulting matrices from PIPseeker were imported into R (version 4.5.1) and processed using Monocle3 (monocle3_1.4.26)^180^^,^^181^^,^^182^ for downstream analysis. Matrices were aggregated, and first-pass filtering for cell quality was performed, keeping cells with <25% reads coming from mitochondrial transcripts and displaying total transcript numbers between 500 and 10,000 transcripts per cell. Dimension reduction was performed using the top 3234 transcripts displaying high variance across the data using UMAP dimension reduction^183^ on the top 17 PCAs (umap.min_dist = 0.1, umap.n.neighbors = 15,umap.metric = “euclidean”). Cells were clustered within the reduced dimension space using the cluster_cells function (reduction_method = “UMAP”, cluster_method = “louvain”, num_iter = 5, partition_qval = 0.01). Cell type calls were performed based on enrichment of transcripts within individual clusters using established markers.^48^ Similar analyses were utilized to determine major amacrine cell subtypes after subsetting of amacrine cells from the total datasets. Fastq files are deposited at Gene Expression Omnibus (GEO, https://www.ncbi.nlm.nih.gov/geo), accession number GSE288381.

#### RNA extraction and RNA sequencing

Total RNA was isolated using the RNeasy Plus Mini Kit (Qiagen, Hilden, Germany) per the manufacturer’s instructions. RNA quantity and quality were assessed via NanoDrop 2000 (Thermo Scientific, Waltham, MA, USA) as well as the RNA 6000 Pico Kit (Agilent Technologies, Santa Clara, CA, USA) for 2100 Bioanalyzer Systems. All RNA samples were submitted to the NYU Langone Health Genome Technology Center for processing.

The SMART-Seq HT Kit (Takara) was utilized to generate complementary DNA (cDNA) from 1 ng of total RNA input. The synthesis process included 13 PCR cycles. The quality and concentration of the stock RNA were assessed using an Agilent Pico Chip on the Bioanalyzer system, ensuring high-quality RNA inputs. For library preparation, cDNA was quantified using the Invitrogen Quant-iT system and subsequently diluted to a concentration of 0.3 ng/μL for library preparation. Libraries were prepared using the Nextera XT DNA Library Preparation Kit, employing a total of 12 PCR cycles to amplify and uniquely barcode the samples. The quality of the generated libraries was assessed using the Agilent High Sensitivity TapeStation to verify fragment sizes and integrity. Additionally, the concentration of libraries was measured with the Invitrogen Quant-iT system to ensure accurate normalization before pooling. For sequencing, libraries were pooled in equimolar ratios and sequenced on the Illumina NovaSeq X+ platform using paired-end 100-cycle reads.

Small RNA libraries were prepared from 10 ng of purified miRNA using the SMARTer smRNA-Seq Kit for Illumina (Takara Bio), following the manufacturer’s protocol. The kit utilizes a ligation-free method based on SMART (Switching Mechanism at the 5′ end of RNA Template) technology to generate high-quality libraries from low-input small RNA. After adapter ligation and cDNA synthesis, libraries were amplified using PCR and purified using AMPure XP beads according to the protocol specifications. The quality and size distribution of the final libraries were assessed using the Agilent High Sensitivity D1000 ScreenTape on the TapeStation 4200 system (Agilent Technologies) to confirm the presence of expected small RNA insert sizes (∼140–150 bp for miRNA). Library concentrations were measured using the Quant-iT dsDNA High Sensitivity Assay Kit (Invitrogen) to ensure accurate quantification. Libraries were normalized to equimolar concentrations based on fluorometric measurements and pooled accordingly. Sequencing was performed on the Illumina NovaSeq X+ platform using paired-end 100-cycle reads, providing high-depth coverage suitable for small RNA transcriptome analysis.

#### Bulk RNA-seq data processing and analysis

All sequencing data were processed by the Applied Bioinformatics Laboratories (ABL) at New York University School of Medicine using standard bioinformatics pipelines. Cutadapt (v5.0)^184^ was used to cut the adaptors from the small RNA-seq data. Sequencing reads from both small and large RNA-Seq projects were mapped to the reference genome (mm10) using the STAR aligner (v2.5.0c).^185^ Alignments were guided by a Gene Transfer Format (GTF) file. The mean read insert sizes and their standard deviations were calculated using Picard tools (v.1.126) (http://broadinstitute.github.io/picard). The read count tables were generated using HTSeq (v0.6.0),^186^ normalized based on their library size factors using DEseq2,^187^ and differential expression analysis was performed. The Read Per Million (RPM) normalized BigWig files were generated using BEDTools (v2.17.0)^188^ and bedGraphToBigWig tool (v4). Gene set enrichment analysis was performed using GSEA tool^189^. To compare the level of similarity among the samples and their replicates, we used two methods: principal-component analysis and Euclidean distance-based sample clustering. All the downstream statistical analyses and generating heatmaps and plots were performed in R environment (v3.1.1) (https://www.r-project.org/). Because of the possibility of a global effect in mature miRNA levels in our Dicer1 knockout (cKO) model, we made count tables for all genes and used the none miRNA genes as our spike-in factor. Spike-in normalization is generally used when gene expression is affected globally. We then used Geometric Library Size Factor (GLSF) normalization as described in DESeq R package for our spike-in factors and applied it to normalize the miRNA genes. Raw files (fastq) and RPM-normalized BigWig files were used to visualize genomic data in interactive genomic browsers (IGV). Fastq files and Bigwig files were deposited at Gene Expression Omnibus (GEO, https://www.ncbi.nlm.nih.gov/geo), accession number GSE288381.

#### miRNA-target interaction analysis and prediction tools to identify RPC miRNA targets

Different prediction tools and genome browsers were used to find putative targets (correct and latest transcript) of late RPC miRNAs (Nanostring dataset from,^101^ available at GEO (GSE135835, under SuperSeries accession number GSE135846), including miRWalk, STarMir, and microT (Diana tools).^161^ miRWalk (http://mirwalk.umm.uni-heidelberg.de) is a database of predicted, as well as experimentally validated, miRNA-target interaction pairs within the complete sequence of genes resulting from the TarPmiR algorithm. TarPmiR is a random-forest-based approach that was trained with 13 features, including Seed match, Accessibility, AU content, exon preference, and the length of the target mRNA region. It also integrates results from other databases with predicted and validated miRNA-target interactions^190^; STarMir web server (http://sfold.wadsworth.org/starmir.html) is an application module of the Sfold RNA package (http://sfold.wadsworth.org), which predicts miRNA binding sites on a target mRNA.^104^ STarMir is a logistic prediction model developed with miRNA binding data from CLIP studies, which predicts miRNA binding sites and computes comprehensive sequence, thermodynamic, and target structure features for any given gene. The sequence information for several miRNAs (miRNA IDs) and our single target mRNA (RefSeq ID) were entered manually, and the HITS-CLIP mouse model for prediction was selected. The miRNA-mRNA binding sites results predicted from the STarMir web server range from one up to around 40 hybrid conformations, which were cross-referenced with those from miRWalk. In contrast to TargetScan, these tools utilize the most current and up-to-date mRNA transcript (https://mart.ensembl.org/info/genome/genebuild/canonical.html), resulting in updated matches of miRNA:mRNA interactions. However, TargetScan was also used as a reference.

#### Luciferase assay

The 3′ UTR of *Elavl3* (obtained from UCSC Genome Browser, https://genome.ucsc.edu/), was cloned into the IV-80 pmiRGLO vector (Addgene plasmid #78128, obtained from Dr. Volker Busskamp, University of Bonn,.^191^ The restriction enzymes NheI and XbaI were used with specific primers for the 3′ UTR region to generate the construct (Table S6, key resources table) as previously described.^164^ The empty IV-80 vector (without a 3′UTR) was a negative control for the assay. These constructs were transfected with mimics (Dharmacon Horizon, Lafayette, CO, USA) for the RPC miRNA miR-25-3p, miR-20a-5p, miR-15a-5p, and the proneuronal miRNA miR-124-3p, as well as a control mimic (C.elegans, Table S9, KST). Four independent experiments were conducted with three technical replicates using HEK293T cells (ATCC, obtained from Dr. Ching-Hwa Sung, Cornell University) and Lipofectamine 3000 (L3000015, Invitrogen, Waltham, MA, USA). 48 h after transfection, Dual-Glo Luciferase reagents were added to the cells (E2920, Promega, Madison, WI, USA). Luciferase activity (Firefly and Renilla) was measured using a plate reader (FilterMax F3, Molecular Devices, San Jose, CA, USA). Data was normalized against negative controls (Plasmid only), and RPC-miRNA treatments were compared to control mimic-transfected conditions. Six independent experiments were conducted with three technical replicates.

### Quantification and statistical analysis

Statistical analyses were performed using SPSS or Sigma Plot by using the Mann-Whitney U-Test, a non-parametric test for independent samples. The Mann–Whitney U test is preferable to the t-test when the data are ordinal but not interval-scaled, hence the spacing between adjacent values of the scale cannot be assumed to be constant. The Holm-Bonferroni method was used to correct for multiple comparisons. Based on power analysis (85% power, alpha 0.5) and previous studies, an n of at least five mice per group was used (detailed numbers below). Only animals that could not be adequately analyzed, i.e., methodological complications (for instance, anesthesia complications, noisy ERG recordings, missing or insufficient reporter labeling) were excluded, which was rarely the case. For randomization and to reduce the badge effect, animals of several litters were analyzed. The genotype or any other identifier was withheld from the investigator evaluating cell numbers or expression levels at the time point of analysis to guarantee unbiased results. All details about the particular sample size n, biological and technical replicates, information about the region analyzed, data shown (mean, SD, or SEM), confidence intervals, and *p*-values are given in the main results text, Figures, Figure legends, and the Supplemental Figures and their legends. The number of biological and technical replicates, as well as the statistical test information is also given below for the particular method:

#### OCT

Mice of various litters and of both sexes were evaluated by three independent investigators. The particular animal numbers per group were P28 wt *n* = 13, P28 cKO *n* = 10, P56 wt *n* = 8, P56 cKO *n* = 9, 3-month wt *n* = 8, 3-month cKO *n* = 4. Data are shown as mean ± S.D. Statistical analyses were conducted using the Mann-Whitney test, *p* ≤ 0.05 indicated by ∗∗, and *p* ≤ 0.01 indicated by ∗∗∗ were assumed as significantly different.

#### ERG

Mice of various litters and of both sexes were evaluated by three independent investigators. The particular animal numbers per group were: P28 wt *n* = 9, P28 cKO *n* = 10, P56 wt *n* = 11, P56 cKO *n* = 7, 3-month wt *n* = 5, 3-month cKO *n* = 5. Data are shown as mean ± SEM. Statistical analysis was conducted using the Mann-Whitney test, *p* ≤ 0.05 indicated by ∗∗, and *p* ≤ 0.01 indicated by ∗∗∗ were assumed as significantly different.

#### Micrograph evaluation and cell counts

Mice of various litters and of both sexes (if determinable) were evaluated by three independent investigators. The numbers per group for young mice were: P4 wt *n* = 4, P4 cKO *n* = 2, P7 wt *n* = 4, P7 cKO *n* = 4, P14 wt *n* = 4, P14 cKO *n* = 3, P21 wt *n* = 5, at least 4 images per retina or RPE. The numbers per group for adult mice were: P28 wt *n* = 5, P28 cKO *n* = 4, P56 wt: *n* = 6, P56 cKO *n* = 7, 3-month wt *n* = 3, 3-month cKO *n* = 4, 4-month-old cKO *n* = 4; For cell quantification, the particular mouse numbers were: Sox9/GS quantification (4 images per mouse): P56 wt: *n* = 7, P56 cKO: *n* = 7; Otx2+ photoreceptor (PR) rows evaluation (10 averaged values per image) in the central or peripheral ONL in wt (*n* = 8, baseline), P28 cKO (*n* = 4), P56 cKO (*n* = 6), 3-month cKO (*n* = 4), and 4-month-old cKO mice (*n* = 4); Otx2/PKC quantification (4 images per mouse): P56 wt: *n* = 6, P56 cKO: *n* = 6; Sox9/GS HuC/D/Pax6 quantification (4 images per mouse): P56 wt: *n* = 5, P56 cKO: *n* = 6; Calbindin, Calretinin quantification (4 images per mouse): P56 wt: *n* = 6, P56 cKO: *n* = 6; All value are expressed as mean ± S.D., Statistical analyses were conducted using the Mann-Whitney test, ∗: *p* ≤ 0.05; ∗∗: *p* ≤ 0.01; ∗∗∗: *p* ≤ 0.001 were assumed as significantly different.

#### Bulk and scRNA-seq

For bulk RNA seq (siRNA and mRNA), a total of 4 P7 wild type and 4 Dicer-cKO mice were pooled and processed in 4 independent experiments per condition. Data is expressed in normalized counts. For scRNA-Seq, 3 P7 wt and 3 P7 cKO mice were pooled, one technical replicate per condition.

#### Luciferase assay

Six independent experiments were conducted with three technical replicates. Data is given in mean ± S.D., Mann-Whitney U-Test: ∗: *p* < 0.05.

## References

[1] Cook T. (2003). Cell diversity in the retina: more than meets the eye. Bioessays.

[2] Jin K., Xiang M. (2017). Transitional Progenitors during Vertebrate Retinogenesis. Mol. Neurobiol..

[3] Marquardt T., Gruss P. (2002). Generating neuronal diversity in the retina: one for nearly all. Trends Neurosci..

[4] Rapaport D.H., Wong L.L., Wood E.D., Yasumura D., LaVail M.M. (2004). Timing and topography of cell genesis in the rat retina. J. Comp. Neurol..

[5] Xiang M. (2013). Intrinsic control of mammalian retinogenesis. Cell. Mol. Life Sci..

[6] Andreazzoli M. (2009). Molecular regulation of vertebrate retina cell fate. Birth Defects Res. C Embryo Today..

[7] Hoang T., Wang J., Boyd P., Wang F., Santiago C., Jiang L., Yoo S., Lahne M., Todd L.J., Jia M. (2020). Gene regulatory networks controlling vertebrate retinal regeneration. Science.

[8] Hahn J., Monavarfeshani A., Qiao M., Kao A.H., Kölsch Y., Kumar A., Kunze V.P., Rasys A.M., Richardson R., Wekselblatt J.B. (2023). Evolution of neuronal cell classes and types in the vertebrate retina. Nature.

[9] Young R.W. (1985). Cell differentiation in the retina of the mouse. Anat. Rec..

[10] Prada C., Puga J., Pérez-Méndez L., Ramírez G., López And R., Ramírez G. (1991). Spatial and Temporal Patterns of Neurogenesis in the Chick Retina. Eur. J. Neurosci..

[11] LaVail M.M., Unoki K., Yasumura D., Matthes M.T., Yancopoulos G.D., Steinberg R.H. (1992). Multiple growth factors, cytokines, and neurotrophins rescue photoreceptors from the damaging effects of constant light. Proc. Natl. Acad. Sci. USA.

[12] Cepko C.L., Austin C.P., Yang X., Alexiades M., Ezzeddine D. (1996). Cell fate determination in the vertebrate retina. Proc. Natl. Acad. Sci. USA.

[13] Brzezinski J.A., Kim E.J., Johnson J.E., Reh T.A. (2011). Ascl1 expression defines a subpopulation of lineage-restricted progenitors in the mammalian retina. Development.

[14] Prusky G.T., Alam N.M., Beekman S., Douglas R.M. (2004). Rapid quantification of adult and developing mouse spatial vision using a virtual optomotor system. Investig. Ophthalmol. Vis. Sci..

[15] Bian S., Xu T.L., Sun T. (2013). Tuning the cell fate of neurons and glia by microRNAs. Curr. Opin. Neurobiol..

[16] Foshay K.M., Gallicano G.I. (2009). miR-17 family miRNAs are expressed during early mammalian development and regulate stem cell differentiation. Dev. Biol..

[17] Lee S.W., Oh Y.M., Lu Y.L., Kim W.K., Yoo A.S. (2018). MicroRNAs Overcome Cell Fate Barrier by Reducing EZH2-Controlled REST Stability during Neuronal Conversion of Human Adult Fibroblasts. Dev. Cell.

[18] Lu D., Davis M.P.A., Abreu-Goodger C., Wang W., Campos L.S., Siede J., Vigorito E., Skarnes W.C., Dunham I., Enright A.J., Liu P. (2012). MiR-25 regulates Wwp2 and Fbxw7 and promotes reprogramming of mouse fibroblast cells to iPSCs. PLoS One.

[19] He L., Hannon G.J. (2004). MicroRNAs: small RNAs with a big role in gene regulation. Nat. Rev. Genet..

[20] Fairchild C.L.A., Cheema S.K., Wong J., Hino K., Simó S., La Torre A. (2019). Let-7 regulates cell cycle dynamics in the developing cerebral cortex and retina. Sci. Rep..

[21] La Torre A., Georgi S., Reh T.A. (2013). Conserved microRNA pathway regulates developmental timing of retinal neurogenesis. Proc. Natl. Acad. Sci. USA.

[22] Akerblom M., Jakobsson J. (2013). MicroRNAs as Neuronal Fate Determinants. Neuroscientist.

[23] Ambros V. (2011). MicroRNAs and developmental timing. Curr. Opin. Genet. Dev..

[24] Amini-Farsani Z., Asgharzade S. (2020). The impact of miR-183/182/96 gene regulation on the maturation, survival, and function of photoreceptor cells in the retina. J. Comp. Neurol..

[25] Bueno M.J., Malumbres M. (2011). MicroRNAs and the cell cycle. Biochim. Biophys. Acta.

[26] Coolen M., Katz S., Bally-Cuif L. (2013). miR-9: a versatile regulator of neurogenesis. Front. Cell. Neurosci..

[27] Corbin R., Olsson-Carter K., Slack F. (2009). The role of microRNAs in synaptic development and function. BMB Rep..

[28] Sun A.X., Crabtree G.R., Yoo A.S. (2013). MicroRNAs: regulators of neuronal fate. Curr. Opin. Cell Biol..

[29] Vo N.K., Cambronne X.A., Goodman R.H. (2010). MicroRNA pathways in neural development and plasticity. Curr. Opin. Neurobiol..

[30] Lee R.C., Feinbaum R.L., Ambros V. (1993). The C. elegans heterochronic gene lin-4 encodes small RNAs with antisense complementarity to lin-14. Cell.

[31] Gurtan A.M., Sharp P.A. (2013). The role of miRNAs in regulating gene expression networks. J. Mol. Biol..

[32] Sundermeier T.R., Palczewski K. (2012). The physiological impact of microRNA gene regulation in the retina. Cell. Mol. Life Sci..

[33] Zuzic M., Rojo Arias J.E., Wohl S.G., Busskamp V. (2019). Retinal miRNA Functions in Health and Disease. Genes.

[34] Damiani D., Alexander J.J., O'Rourke J.R., McManus M., Jadhav A.P., Cepko C.L., Hauswirth W.W., Harfe B.D., Strettoi E. (2008). Dicer inactivation leads to progressive functional and structural degeneration of the mouse retina. J. Neurosci..

[35] Decembrini S., Andreazzoli M., Barsacchi G., Cremisi F. (2008). Dicer inactivation causes heterochronic retinogenesis in Xenopus laevis. Int. J. Dev. Biol..

[36] Iida A., Shinoe T., Baba Y., Mano H., Watanabe S. (2011). Dicer plays essential roles for retinal development by regulation of survival and differentiation. Investig. Ophthalmol. Vis. Sci..

[37] Pinter R., Hindges R. (2010). Perturbations of microRNA function in mouse dicer mutants produce retinal defects and lead to aberrant axon pathfinding at the optic chiasm. PLoS One.

[38] Georgi S.A., Reh T.A. (2010). Dicer is required for the transition from early to late progenitor state in the developing mouse retina. J. Neurosci..

[39] Davis N., Mor E., Ashery-Padan R. (2011). Roles for Dicer1 in the patterning and differentiation of the optic cup neuroepithelium. Development.

[40] Kim E.J., Ables J.L., Dickel L.K., Eisch A.J., Johnson J.E. (2011). Ascl1 (Mash1) defines cells with long-term neurogenic potential in subgranular and subventricular zones in adult mouse brain. PLoS One.

[41] Kim E.J., Leung C.T., Reed R.R., Johnson J.E. (2007). In vivo analysis of Ascl1 defined progenitors reveals distinct developmental dynamics during adult neurogenesis and gliogenesis. J. Neurosci..

[42] Nelson B.R., Ueki Y., Reardon S., Karl M.O., Georgi S., Hartman B.H., Lamba D.A., Reh T.A. (2011). Genome-wide analysis of Muller glial differentiation reveals a requirement for Notch signaling in postmitotic cells to maintain the glial fate. PLoS One.

[43] Harfe B.D., McManus M.T., Mansfield J.H., Hornstein E., Tabin C.J. (2005). The RNaseIII enzyme Dicer is required for morphogenesis but not patterning of the vertebrate limb. Proc. Natl. Acad. Sci. USA.

[44] Young R.W. (1985). Cell proliferation during postnatal development of the retina in the mouse. Brain Res..

[45] Godecke N., Zha L., Spencer S., Behme S., Riemer P., Rehli M., Hauser H., Wirth D. (2017). Controlled re-activation of epigenetically silenced Tet promoter-driven transgene expression by targeted demethylation. Nucleic Acids Res..

[46] Long M.A., Rossi F.M.V. (2009). Silencing inhibits Cre-mediated recombination of the Z/AP and Z/EG reporters in adult cells. PLoS One.

[47] Whitney I.E., Keeley P.W., St John A.J., Kautzman A.G., Kay J.N., Reese B.E. (2014). Sox2 regulates cholinergic amacrine cell positioning and dendritic stratification in the retina. J. Neurosci..

[48] Clark B.S., Stein-O'Brien G.L., Shiau F., Cannon G.H., Davis-Marcisak E., Sherman T., Santiago C.P., Hoang T.V., Rajaii F., James-Esposito R.E. (2019). Single-Cell RNA-Seq Analysis of Retinal Development Identifies NFI Factors as Regulating Mitotic Exit and Late-Born Cell Specification. Neuron.

[49] Blackshaw S., Harpavat S., Trimarchi J., Cai L., Huang H., Kuo W.P., Weber G., Lee K., Fraioli R.E., Cho S.H. (2004). Genomic analysis of mouse retinal development. PLoS Biol..

[50] Macosko E.Z., Basu A., Satija R., Nemesh J., Shekhar K., Goldman M., Tirosh I., Bialas A.R., Kamitaki N., Martersteck E.M. (2015). Highly Parallel Genome-wide Expression Profiling of Individual Cells Using Nanoliter Droplets. Cell.

[51] Wohl S.G., Reh T.A. (2016). The microRNA expression profile of mouse Muller glia in vivo and in vitro. Sci. Rep..

[52] Jadhav A.P., Roesch K., Cepko C.L. (2009). Development and neurogenic potential of Müller glial cells in the vertebrate retina. Prog. Retin. Eye Res..

[53] Roesch K., Jadhav A.P., Trimarchi J.M., Stadler M.B., Roska B., Sun B.B., Cepko C.L. (2008). The transcriptome of retinal Müller glial cells. J. Comp. Neurol..

[54] Akimoto M., Cheng H., Zhu D., Brzezinski J.A., Khanna R., Filippova E., Oh E.C.T., Jing Y., Linares J.L., Brooks M. (2006). Targeting of GFP to newborn rods by Nrl promoter and temporal expression profiling of flow-sorted photoreceptors. Proc. Natl. Acad. Sci. USA.

[55] Shekhar K., Lapan S.W., Whitney I.E., Tran N.M., Macosko E.Z., Kowalczyk M., Adiconis X., Levin J.Z., Nemesh J., Goldman M. (2016). Comprehensive Classification of Retinal Bipolar Neurons by Single-Cell Transcriptomics. Cell.

[56] Kim J.W., Yang H.J., Brooks M.J., Zelinger L., Karakülah G., Gotoh N., Boleda A., Gieser L., Giuste F., Whitaker D.T. (2016). NRL-Regulated Transcriptome Dynamics of Developing Rod Photoreceptors. Cell Rep..

[57] Bumsted-O'Brien K.M., Hendrickson A., Haverkamp S., Ashery-Padan R., Schulte D. (2007). Expression of the homeodomain transcription factor Meis2 in the embryonic and postnatal retina. J. Comp. Neurol..

[58] Dupacova N., Antosova B., Paces J., Kozmik Z. (2021). Meis homeobox genes control progenitor competence in the retina. Proc. Natl. Acad. Sci. USA.

[59] Yan W., Laboulaye M.A., Tran N.M., Whitney I.E., Benhar I., Sanes J.R. (2020). Mouse Retinal Cell Atlas: Molecular Identification of over Sixty Amacrine Cell Types. J. Neurosci..

[60] Dyer M.A., Cepko C.L. (2001). p27Kip1 and p57Kip2 regulate proliferation in distinct retinal progenitor cell populations. J. Neurosci..

[61] Dyer M.A., Cepko C.L. (2001). The p57Kip2 cyclin kinase inhibitor is expressed by a restricted set of amacrine cells in the rodent retina. J. Comp. Neurol..

[62] Lu Y.L., Liu Y., McCoy M.J., Yoo A.S. (2021). MiR-124 synergism with ELAVL3 enhances target gene expression to promote neuronal maturity. Proc. Natl. Acad. Sci. USA.

[63] Wu M., Deng Q., Lei X., Du Y., Shen Y. (2021). Elavl2 Regulates Retinal Function Via Modulating the Differentiation of Amacrine Cells Subtype. Investig. Ophthalmol. Vis. Sci..

[64] Bronicki L.M., Jasmin B.J. (2013). Emerging complexity of the HuD/ELAVl4 gene; implications for neuronal development, function, and dysfunction. RNA.

[65] Wutikeli H., Yu Y., Zhang T., Cao J., Nawy S., Shen Y. (2025). Role of Elavl-like RNA-binding protein in retinal development and signal transduction. Biochim. Biophys. Acta. Mol. Basis Dis..

[66] Yokoi S., Udagawa T., Fujioka Y., Honda D., Okado H., Watanabe H., Katsuno M., Ishigaki S., Sobue G. (2017). 3'UTR Length-Dependent Control of SynGAP Isoform alpha2 mRNA by FUS and ELAV-like Proteins Promotes Dendritic Spine Maturation and Cognitive Function. Cell Rep..

[67] Wohl S.G., Jorstad N.L., Levine E.M., Reh T.A. (2017). Muller glial microRNAs are required for the maintenance of glial homeostasis and retinal architecture. Nat. Commun..

[68] Ferreira H., Martins J., Moreira P.I., Ambrósio A.F., Castelo-Branco M., Serranho P., Bernardes R. (2021). Longitudinal normative OCT retinal thickness data for wild-type mice, and characterization of changes in the 3xTg-AD mice model of Alzheimer's disease. Aging.

[69] Ferguson L.R., Grover S., Dominguez J.M., Balaiya S., Chalam K.V. (2014). Retinal thickness measurement obtained with spectral domain optical coherence tomography assisted optical biopsy accurately correlates with ex vivo histology. PLoS One.

[70] Larbi D., Rief A.M., Kang S., Chen S., Batsuuri K., Fuhrmann S., Viswanathan S., Wohl S.G. (2025). Dicer Loss in Muller Glia Leads to a Defined Sequence of Pathological Events Beginning With Cone Dysfunction. Investig. Ophthalmol. Vis. Sci..

[71] Li Y., Zhang Y., Chen S., Vernon G., Wong W.T., Qian H. (2018). Light-Dependent OCT Structure Changes in Photoreceptor Degenerative rd 10 Mouse Retina. Investig. Ophthalmol. Vis. Sci..

[72] Adachi K., Takahashi S., Yamauchi K., Mounai N., Tanabu R., Nakazawa M. (2016). Optical Coherence Tomography of Retinal Degeneration in Royal College of Surgeons Rats and Its Correlation with Morphology and Electroretinography. PLoS One.

[73] Jones B.W., Marc R.E. (2005). Retinal remodeling during retinal degeneration. Exp. Eye Res..

[74] Ohana R., Weiman-Kelman B., Raviv S., Tamm E.R., Pasmanik-Chor M., Rinon A., Netanely D., Shamir R., Solomon A.S., Ashery-Padan R. (2015). MicroRNAs are essential for differentiation of the retinal pigmented epithelium and maturation of adjacent photoreceptors. Development.

[75] Sundermeier T.R., Sakami S., Sahu B., Howell S.J., Gao S., Dong Z., Golczak M., Maeda A., Palczewski K. (2017). MicroRNA-processing Enzymes Are Essential for Survival and Function of Mature Retinal Pigmented Epithelial Cells in Mice. J. Biol. Chem..

[76] Greferath U., Grünert U., Wässle H. (1990). Rod bipolar cells in the mammalian retina show protein kinase C-like immunoreactivity. J. Comp. Neurol..

[77] Wassle H., Yamashita M., Greferath U., Grunert U., Muller F. (1991). The rod bipolar cell of the mammalian retina. Vis. Neurosci..

[78] Kato M., Sudou N., Nomura-Komoike K., Iida T., Fujieda H. (2022). Age- and cell cycle-related expression patterns of transcription factors and cell cycle regulators in Muller glia. Sci. Rep..

[79] Joly S., Pernet V., Samardzija M., Grimm C. (2011). Pax6-positive Muller glia cells express cell cycle markers but do not proliferate after photoreceptor injury in the mouse retina. Glia.

[80] Bernardos R.L., Barthel L.K., Meyers J.R., Raymond P.A. (2007). Late-stage neuronal progenitors in the retina are radial Müller glia that function as retinal stem cells. J. Neurosci..

[81] Gargini C., Terzibasi E., Mazzoni F., Strettoi E. (2007). Retinal organization in the retinal degeneration 10 (rd10) mutant mouse: a morphological and ERG study. J. Comp. Neurol..

[82] Voinescu P.E., Kay J.N., Sanes J.R. (2009). Birthdays of retinal amacrine cell subtypes are systematically related to their molecular identity and soma position. J. Comp. Neurol..

[83] Balasubramanian R., Gan L. (2014). Development of Retinal Amacrine Cells and Their Dendritic Stratification. Curr. Ophthalmol. Rep..

[84] Perron M., Furrer M.P., Wegnez M., Théodore L. (1999). Xenopus elav-like genes are differentially expressed during neurogenesis. Mech. Dev..

[85] Good P.J. (1995). A conserved family of elav-like genes in vertebrates. Proc. Natl. Acad. Sci. USA.

[86] Amato M.A., Boy S., Arnault E., Girard M., Della Puppa A., Sharif A., Perron M. (2005). Comparison of the expression patterns of five neural RNA binding proteins in the Xenopus retina. J. Comp. Neurol..

[87] Lagnado L. (1998). Retinal processing: amacrine cells keep it short and sweet. Curr. Biol..

[88] Cheloufi S., Dos Santos C.O., Chong M.M.W., Hannon G.J. (2010). A dicer-independent miRNA biogenesis pathway that requires Ago catalysis. Nature.

[89] Chong M.M.W., Zhang G., Cheloufi S., Neubert T.A., Hannon G.J., Littman D.R. (2010). Canonical and alternate functions of the microRNA biogenesis machinery. Genes Dev..

[90] Kim Y.K., Kim B., Kim V.N. (2016). Re-evaluation of the roles of DROSHA, Export in 5, and DICER in microRNA biogenesis. Proc. Natl. Acad. Sci. USA.

[91] Mattar P., Ericson J., Blackshaw S., Cayouette M. (2015). A conserved regulatory logic controls temporal identity in mouse neural progenitors. Neuron.

[92] Mattar P., Jolicoeur C., Dang T., Shah S., Clark B.S., Cayouette M. (2021). A Casz1-NuRD complex regulates temporal identity transitions in neural progenitors. Sci. Rep..

[93] Mattar P., Stevanovic M., Nad I., Cayouette M. (2018). Casz1 controls higher-order nuclear organization in rod photoreceptors. Proc. Natl. Acad. Sci. USA.

[94] Poche R.A., Furuta Y., Chaboissier M.C., Schedl A., Behringer R.R. (2008). Sox9 is expressed in mouse multipotent retinal progenitor cells and functions in Muller glial cell development. J. Comp. Neurol..

[95] Zhu M.Y., Gasperowicz M., Chow R.L. (2013). The expression of NOTCH2, HES1 and SOX9 during mouse retinal development. Gene Expr. Patterns.

[96] Tomita K., Ishibashi M., Nakahara K., Ang S.L., Nakanishi S., Guillemot F., Kageyama R. (1996). Mammalian hairy and Enhancer of split homolog 1 regulates differentiation of retinal neurons and is essential for eye morphogenesis. Neuron.

[97] Kageyama R., Ishibashi M., Takebayashi K., Tomita K. (1997). bHLH transcription factors and mammalian neuronal differentiation. Int. J. Biochem. Cell Biol..

[98] Furukawa T., Mukherjee S., Bao Z.Z., Morrow E.M., Cepko C.L. (2000). rax, Hes1, and notch1 promote the formation of Muller glia by postnatal retinal progenitor cells. Neuron.

[99] Takatsuka K., Hatakeyama J., Bessho Y., Kageyama R. (2004). Roles of the bHLH gene Hes1 in retinal morphogenesis. Brain Res..

[100] Wohl S.G., D’Amore P.A. (2025). Encyclopedia of the Eye, vol. 1.

[101] Genini S., Guziewicz K.E., Beltran W.A., Aguirre G.D. (2014). Altered miRNA expression in canine retinas during normal development and in models of retinal degeneration. BMC Genomics.

[102] Hackler L., Wan J., Swaroop A., Qian J., Zack D.J. (2010). MicroRNA profile of the developing mouse retina. Invest. Ophthalmol. Vis. Sci..

[103] Wohl S.G., Hooper M.J., Reh T.A. (2019). MicroRNAs miR-25, let-7 and miR-124 regulate the neurogenic potential of Muller glia in mice. Development.

[104] Rennie W., Liu C., Carmack C.S., Wolenc A., Kanoria S., Lu J., Long D., Ding Y. (2014). STarMir: a web server for prediction of microRNA binding sites. Nucleic Acids Res..

[105] De Pietri Tonelli D., Pulvers J.N., Haffner C., Murchison E.P., Hannon G.J., Huttner W.B. (2008). miRNAs are essential for survival and differentiation of newborn neurons but not for expansion of neural progenitors during early neurogenesis in the mouse embryonic neocortex. Development.

[106] Davis T.H., Cuellar T.L., Koch S.M., Barker A.J., Harfe B.D., McManus M.T., Ullian E.M. (2008). Conditional loss of Dicer disrupts cellular and tissue morphogenesis in the cortex and hippocampus. J. Neurosci..

[107] Kawase-Koga Y., Low R., Otaegi G., Pollock A., Deng H., Eisenhaber F., Maurer-Stroh S., Sun T. (2010). RNAase-III enzyme Dicer maintains signaling pathways for differentiation and survival in mouse cortical neural stem cells. J. Cell Sci..

[108] McLoughlin H.S., Fineberg S.K., Ghosh L.L., Tecedor L., Davidson B.L. (2012). Dicer is required for proliferation, viability, migration and differentiation in corticoneurogenesis. Neuroscience.

[109] Zindy F., Lee Y., Kawauchi D., Ayrault O., Merzoug L.B., Li Y., McKinnon P.J., Roussel M.F. (2015). Dicer Is Required for Normal Cerebellar Development and to Restrain Medulloblastoma Formation. PLoS One.

[110] Rajaram K., Harding R.L., Bailey T., Patton J.G., Hyde D.R. (2014). Dynamic miRNA expression patterns during retinal regeneration in zebrafish: reduced dicer or miRNA expression suppresses proliferation of Muller glia-derived neuronal progenitor cells. Dev. Dyn..

[111] Konar G.J., Ferguson C., Flickinger Z., Kent M.R., Patton J.G. (2020). miRNAs and Muller Glia Reprogramming During Retina Regeneration. Front. Cell Dev. Biol..

[112] Lagos-Quintana M., Rauhut R., Yalcin A., Meyer J., Lendeckel W., Tuschl T. (2002). Identification of tissue-specific microRNAs from mouse. Curr. Biol..

[113] Rowan S., Cepko C.L. (2004). Genetic analysis of the homeodomain transcription factor Chx10 in the retina using a novel multifunctional BAC transgenic mouse reporter. Dev. Biol..

[114] Belecky-Adams T., Tomarev S., Li H.S., Ploder L., McInnes R.R., Sundin O., Adler R. (1997). Pax-6, Prox 1, and Chx10 homeobox gene expression correlates with phenotypic fate of retinal precursor cells. Investig. Ophthalmol. Vis. Sci..

[115] Burmeister M., Novak J., Liang M.Y., Basu S., Ploder L., Hawes N.L., Vidgen D., Hoover F., Goldman D., Kalnins V.I. (1996). Ocular retardation mouse caused by Chx10 homeobox null allele: impaired retinal progenitor proliferation and bipolar cell differentiation. Nat. Genet..

[116] Green E.S., Stubbs J.L., Levine E.M. (2003). Genetic rescue of cell number in a mouse model of microphthalmia: interactions between Chx10 and G1-phase cell cycle regulators. Development.

[117] Sigulinsky C.L., Green E.S., Clark A.M., Levine E.M. (2008). Vsx2/Chx10 ensures the correct timing and magnitude of Hedgehog signaling in the mouse retina. Dev. Biol..

[118] Jin K., Jiang H., Xiao D., Zou M., Zhu J., Xiang M. (2015). Tfap2a and 2b act downstream of Ptf1a to promote amacrine cell differentiation during retinogenesis. Mol. Brain.

[119] Park S.J., Lei W., Pisano J., Orpia A., Minehart J., Pottackal J., Hanke-Gogokhia C., Zapadka T.E., Clarkson-Paredes C., Popratiloff A. (2023). Molecular identification of wide-field amacrine cells in mouse retina that encode stimulus orientation. bioRxiv.

[120] Perez-Leon J.A., Espinal-Centeno A., Mendoza-Gonzalez Z., Camacho A.C., Lopez A.B., Perez R.A., Miranda M. (2022). Identification of amacrine neurons with a glycinergic and GABAergic phenotype in the mouse retina. Med. Res. Arch..

[121] Li S., Mo Z., Yang X., Price S.M., Shen M.M., Xiang M. (2004). Foxn4 controls the genesis of amacrine and horizontal cells by retinal progenitors. Neuron.

[122] Fujitani Y., Fujitani S., Luo H., Qiu F., Burlison J., Long Q., Kawaguchi Y., Edlund H., MacDonald R.J., Furukawa T. (2006). Ptf1a determines horizontal and amacrine cell fates during mouse retinal development. Development.

[123] Sato S., Inoue T., Terada K., Matsuo I., Aizawa S., Tano Y., Fujikado T., Furukawa T. (2007). Dkk3-Cre BAC transgenic mouse line: a tool for highly efficient gene deletion in retinal progenitor cells. Genesis.

[124] Gallina D., Palazzo I., Steffenson L., Todd L., Fischer A.J. (2016). Wnt/beta-catenin-signaling and the formation of Muller glia-derived progenitors in the chick retina. Dev. Neurobiol..

[125] Zhu L., Shen W., Zhang T., Wang Y., Bahrami B., Zhou F., Gillies M.C. (2018). Characterization of canonical Wnt signalling changes after induced disruption of Muller cell in murine retina. Exp. Eye Res..

[126] Muranishi Y., Furukawa T. (2012). BAC-Dkk3-EGFP transgenic mouse: an in vivo analytical tool for Dkk3 expression. J. Biomed. Biotechnol..

[127] Ramachandran R., Zhao X.F., Goldman D. (2011). Ascl1a/Dkk/beta-catenin signaling pathway is necessary and glycogen synthase kinase-3beta inhibition is sufficient for zebrafish retina regeneration. Proc. Natl. Acad. Sci. USA.

[128] Shu J., Xia Z., Li L., Liang E.T., Slipek N., Shen D., Foo J., Subramanian S., Steer C.J. (2012). Dose-dependent differential mRNA target selection and regulation by let-7a-7f and miR-17-92 cluster microRNAs. RNA Biol..

[129] Song X., Liang Y., Sang Y., Li Y., Zhang H., Chen B., Du L., Liu Y., Wang L., Zhao W. (2020). circHMCU Promotes Proliferation and Metastasis of Breast Cancer by Sponging the let-7 Family. Mol. Ther. Nucleic Acids.

[130] Tu F., Li M., Chen Y., Chu H., Wang S., Hai L., Xie T., Geng F., Zhao T., Wang Q., Feng Z. (2022). Let-7i-3p inhibits the cell cycle, proliferation, invasion, and migration of colorectal cancer cells via downregulating CCND1. Open Med..

[131] Wang L.J., Cai H.Q. (2020). Let-7b downgrades CCND1 to repress osteogenic proliferation and differentiation of MC3T3-E1 cells: An implication in osteoporosis. Kaohsiung J. Med. Sci..

[132] Barton K.M., Levine E.M. (2008). Expression patterns and cell cycle profiles of PCNA, MCM6, cyclin D1, cyclin A2, cyclin B1, and phosphorylated histone H3 in the developing mouse retina. Dev. Dyn..

[133] Trimarchi J.M., Stadler M.B., Cepko C.L. (2008). Individual retinal progenitor cells display extensive heterogeneity of gene expression. PLoS One.

[134] Dyer M.A., Cepko C.L. (2000). Control of Muller glial cell proliferation and activation following retinal injury. Nat. Neurosci..

[135] Das G., Choi Y., Sicinski P., Levine E.M. (2009). Cyclin D1 fine-tunes the neurogenic output of embryonic retinal progenitor cells. Neural Dev..

[136] Das G., Clark A.M., Levine E.M. (2012). Cyclin D1 inactivation extends proliferation and alters histogenesis in the postnatal mouse retina. Dev. Dyn..

[137] Goldman D. (2014). Müller glial cell reprogramming and retina regeneration. Nat. Rev. Neurosci..

[138] Ramachandran R., Fausett B.V., Goldman D. (2010). Ascl1a regulates Müller glia dedifferentiation and retinal regeneration through a Lin-28-dependent, let-7 microRNA signalling pathway. Nat. Cell Biol..

[139] Hojo M., Ohtsuka T., Hashimoto N., Gradwohl G., Guillemot F., Kageyama R. (2000). Glial cell fate specification modulated by the bHLH gene Hes5 in mouse retina. Development.

[140] Turner D.L., Cepko C.L. (1987). A common progenitor for neurons and glia persists in rat retina late in development. Nature.

[141] Sundermeier T.R., Zhang N., Vinberg F., Mustafi D., Kohno H., Golczak M., Bai X., Maeda A., Kefalov V.J., Palczewski K. (2014). DICER1 is essential for survival of postmitotic rod photoreceptor cells in mice. FASEB J..

[142] Aldunate E.Z., Di Foggia V., Di Marco F., Hervas L.A., Ribeiro J.C., Holder D.L., Patel A., Jannini T.B., Thompson D.A., Martinez-Barbera J.P. (2019). Conditional Dicer1 depletion using Chrnb4-Cre leads to cone cell death and impaired photopic vision. Sci. Rep..

[143] Grosshans H., Chatterjee S. (2010). MicroRNAses and the regulated degradation of mature animal miRNAs. Adv. Exp. Med. Biol..

[144] Gantier M.P., McCoy C.E., Rusinova I., Saulep D., Wang D., Xu D., Irving A.T., Behlke M.A., Hertzog P.J., Mackay F., Williams B.R.G. (2011). Analysis of microRNA turnover in mammalian cells following Dicer1 ablation. Nucleic Acids Res..

[145] Winter J., Diederichs S. (2011). Argonaute proteins regulate microRNA stability: Increased microRNA abundance by Argonaute proteins is due to microRNA stabilization. RNA Biol..

[146] Perez-Ortin J.E., Alepuz P., Chavez S., Choder M. (2013). Eukaryotic mRNA decay: methodologies, pathways, and links to other stages of gene expression. J. Mol. Biol..

[147] Kingston E.R., Bartel D.P. (2019). Global analyses of the dynamics of mammalian microRNA metabolism. Genome Res..

[148] Yang J.S., Lai E.C. (2011). Alternative miRNA biogenesis pathways and the interpretation of core miRNA pathway mutants. Mol. Cell.

[149] Remez L.A., Onishi A., Menuchin-Lasowski Y., Biran A., Blackshaw S., Wahlin K.J., Zack D.J., Ashery-Padan R. (2017). Pax6 is essential for the generation of late-born retinal neurons and for inhibition of photoreceptor-fate during late stages of retinogenesis. Dev. Biol..

[150] Conte I., Carrella S., Avellino R., Karali M., Marco-Ferreres R., Bovolenta P., Banfi S. (2010). miR-204 is required for lens and retinal development via Meis2 targeting. Proc. Natl. Acad. Sci. USA.

[151] Dyer M.A., Cepko C.L. (2000). p57(Kip2) regulates progenitor cell proliferation and amacrine interneuron development in the mouse retina. Development.

[152] Marquardt T., Ashery-Padan R., Andrejewski N., Scardigli R., Guillemot F., Gruss P. (2001). Pax6 is required for the multipotent state of retinal progenitor cells. Cell.

[153] Oron-Karni V., Farhy C., Elgart M., Marquardt T., Remizova L., Yaron O., Xie Q., Cvekl A., Ashery-Padan R. (2008). Dual requirement for Pax6 in retinal progenitor cells. Development.

[154] Li Y., Shen Y., Cai D., Shen Y. (2021). Sox2 knockdown in the neonatal retina causes cell fate to switch from amacrine to bipolar. Brain Res..

[155] Soller M., White K. (2004). Elav. Curr. Biol..

[156] Perrone-Bizzozero N., Bolognani F. (2002). Role of HuD and other RNA-binding proteins in neural development and plasticity. J. Neurosci. Res..

[157] Mulligan M.R., Bicknell L.S. (2023). The molecular genetics of nELAVL in brain development and disease. Eur. J. Hum. Genet..

[158] Ueki Y., Wilken M.S., Cox K.E., Chipman L., Jorstad N., Sternhagen K., Simic M., Ullom K., Nakafuku M., Reh T.A. (2015). Transgenic expression of the proneural transcription factor Ascl1 in Müller glia stimulates retinal regeneration in young mice. Proc. Natl. Acad. Sci. USA.

[159] Yao K., Qiu S., Tian L., Snider W.D., Flannery J.G., Schaffer D.V., Chen B. (2016). Wnt Regulates Proliferation and Neurogenic Potential of Muller Glial Cells via a Lin28/let-7 miRNA-Dependent Pathway in Adult Mammalian Retinas. Cell Rep..

[160] Le N., Vu T.D., Palazzo I., Pulya R., Kim Y., Blackshaw S., Hoang T. (2023). Robust reprogramming of glia into neurons by inhibition of Notch signaling and NFI factors in adult mammalian retina. bioRxiv.

[161] Tastsoglou S., Alexiou A., Karagkouni D., Skoufos G., Zacharopoulou E., Hatzigeorgiou A.G. (2023). DIANA-microT 2023: including predicted targets of virally encoded miRNAs. Nucleic Acids Res..

[162] Brummer A., Hausser J. (2014). MicroRNA binding sites in the coding region of mRNAs: extending the repertoire of post-transcriptional gene regulation. Bioessays.

[163] Buhagiar A.F., Kleaveland B. (2024). To kill a microRNA: emerging concepts in target-directed microRNA degradation. Nucleic Acids Res..

[164] Kutsche L.K., Gysi D.M., Fallmann J., Lenk K., Petri R., Swiersy A., Klapper S.D., Pircs K., Khattak S., Stadler P.F. (2018). Combined Experimental and System-Level Analyses Reveal the Complex Regulatory Network of miR-124 during Human Neurogenesis. Cell Syst..

[165] Kaufman M.L., Goodson N.B., Park K.U., Schwanke M., Office E., Schneider S.R., Abraham J., Hensley A., Jones K.L., Brzezinski J.A. (2021). Initiation of Otx2 expression in the developing mouse retina requires a unique enhancer and either Ascl1 or Neurog2 activity. Development.

[166] Karampelas M., Sim D.A., Keane P.A. (2014). Spectral-domain OCT of the RPE. Optical coherence tomography has evolved to the extent that we can now image the retinal microstructure. Retin. Physician.

[167] Pandya B.U., Grinton M., Mandelcorn E.D., Felfeli T. (2024). RETINAL OPTICAL COHERENCE TOMOGRAPHY IMAGING BIOMARKERS: A Review of the Literature. Retina.

[168] Severns M.L., Johnson M.A. (1993). The care and fitting of Naka-Rushton functions to electroretinographic intensity-response data. Doc. Ophthalmol..

[169] Evans L.S., Peachey N.S., Marchese A.L. (1993). Comparison of three methods of estimating the parameters of the Naka-Rushton equation. Doc. Ophthalmol..

[170] Cobb W., Morton H.B. (1952). The human retinogram in response to high-intensity flashes. Electroencephalogr. Clin. Neurophysiol..

[171] Liao F., Liu H., Milla-Navarro S., Villa P.d.l., Germain F. (2023). Origin of Retinal Oscillatory Potentials in the Mouse, a Tool to Specifically Locate Retinal Damage. Int. J. Mol. Sci..

[172] Wachtmeister L. (1998). Oscillatory potentials in the retina: what do they reveal. Prog. Retin. Eye Res..

[173] Wachtmeister L., Dowling J.E. (1978). The oscillatory potentials of the mudpuppy retina. Investig. Ophthalmol. Vis. Sci..

[174] Akula J.D., Mocko J.A., Moskowitz A., Hansen R.M., Fulton A.B. (2007). The oscillatory potentials of the dark-adapted electroretinogram in retinopathy of prematurity. Investig. Ophthalmol. Vis. Sci..

[175] Naarendorp F., Sieving P.A. (1991). The scotopic threshold response of the cat ERG is suppressed selectively by GABA and glycine. Vision Res..

[176] Sieving P.A., Frishman L.J., Steinberg R.H. (1986). Scotopic threshold response of proximal retina in cat. J. Neurophysiol..

[177] Saszik S., Bilotta J. (2001). Constant dark-rearing effects on visual adaptation of the zebrafish ERG. Int. J. Dev. Neurosci..

[178] Kang S., Wohl S.G. (2022). Primary Cell Cultures to Study the Regeneration Potential of Murine Muller Glia after MicroRNA Treatment. J. Vis. Exp..

[179] Lee S.H., Won Y., Gibbs D., Caldwell B., Goldstein A., Choi E., Goldenring J.R. (2024). Amphiregulin Switches Progenitor Cell Fate for Lineage Commitment During Gastric Mucosal Regeneration. Gastroenterology.

[180] Cao J., Spielmann M., Qiu X., Huang X., Ibrahim D.M., Hill A.J., Zhang F., Mundlos S., Christiansen L., Steemers F.J. (2019). The single-cell transcriptional landscape of mammalian organogenesis. Nature.

[181] Qiu X., Mao Q., Tang Y., Wang L., Chawla R., Pliner H.A., Trapnell C. (2017). Reversed graph embedding resolves complex single-cell trajectories. Nat. Methods.

[182] Trapnell C., Cacchiarelli D., Grimsby J., Pokharel P., Li S., Morse M., Lennon N.J., Livak K.J., Mikkelsen T.S., Rinn J.L. (2014). The dynamics and regulators of cell fate decisions are revealed by pseudotemporal ordering of single cells. Nat. Biotechnol..

[183] McInnes L., Healy J., Melville J. (2020). UMAP: Uniform Manifold Approximation and Projection for Dimension Reduction. arXiv.

[184] Martin M. (2011). Cutadapt removes adapter sequences from high-throughput sequencing reads. EMBnet. J..

[185] Dobin A., Davis C.A., Schlesinger F., Drenkow J., Zaleski C., Jha S., Batut P., Chaisson M., Gingeras T.R. (2013). STAR: ultrafast universal RNA-seq aligner. Bioinformatics.

[186] Anders S., Pyl P.T., Huber W. (2015). HTSeq--a Python framework to work with high-throughput sequencing data. Bioinformatics.

[187] Love M.I., Huber W., Anders S. (2014). Moderated estimation of fold change and dispersion for RNA-seq data with DESeq2. Genome Biol..

[188] Quinlan A.R., Hall I.M. (2010). BEDTools: a flexible suite of utilities for comparing genomic features. Bioinformatics.

[189] Subramanian A., Tamayo P., Mootha V.K., Mukherjee S., Ebert B.L., Gillette M.A., Paulovich A., Pomeroy S.L., Golub T.R., Lander E.S., Mesirov J.P. (2005). Gene set enrichment analysis: a knowledge-based approach for interpreting genome-wide expression profiles. Proc. Natl. Acad. Sci. USA.

[190] Sticht C., De La Torre C., Parveen A., Gretz N. (2018). miRWalk: An online resource for prediction of microRNA binding sites. PLoS One.

[191] Eichelser C., Stückrath I., Müller V., Milde-Langosch K., Wikman H., Pantel K., Schwarzenbach H. (2014). Increased serum levels of circulating exosomal microRNA-373 in receptor-negative breast cancer patients. Oncotarget.

