# Supplementary material for: Dicer is essential for proper maturation, composition, and function in the postnatal retina
Source: iScience. 2025 Oct 17;28(11):113794. doi: 10.1016/j.isci.2025.113794 (PMC12616091; doi:10.1016/j.isci.2025.113794)
Supplement: Document S1. Figures S1–S10 and Tables S1–S9 [file mmc1.pdf]

iScience, Volume *28*

Supplemental information

Dicer is essential for proper maturation, composition, and function in the postnatal retina

Seoyoung Kang, Daniel Larbi, Eik Bruns, Konstantin Hahne, Alireza Khodadadi-Jamayran, Chaitra Sreenivasaiah, Mariana Lima Carneiro, Monica Andrade, Khulan Batsuuri, Shaoheng Chen, Julia Jager, Suresh Viswanathan, Brian Stewart Clark, and Stefanie Gabriele Wohl

Supplementary
Figures and
Tables

Kang et al., 2025

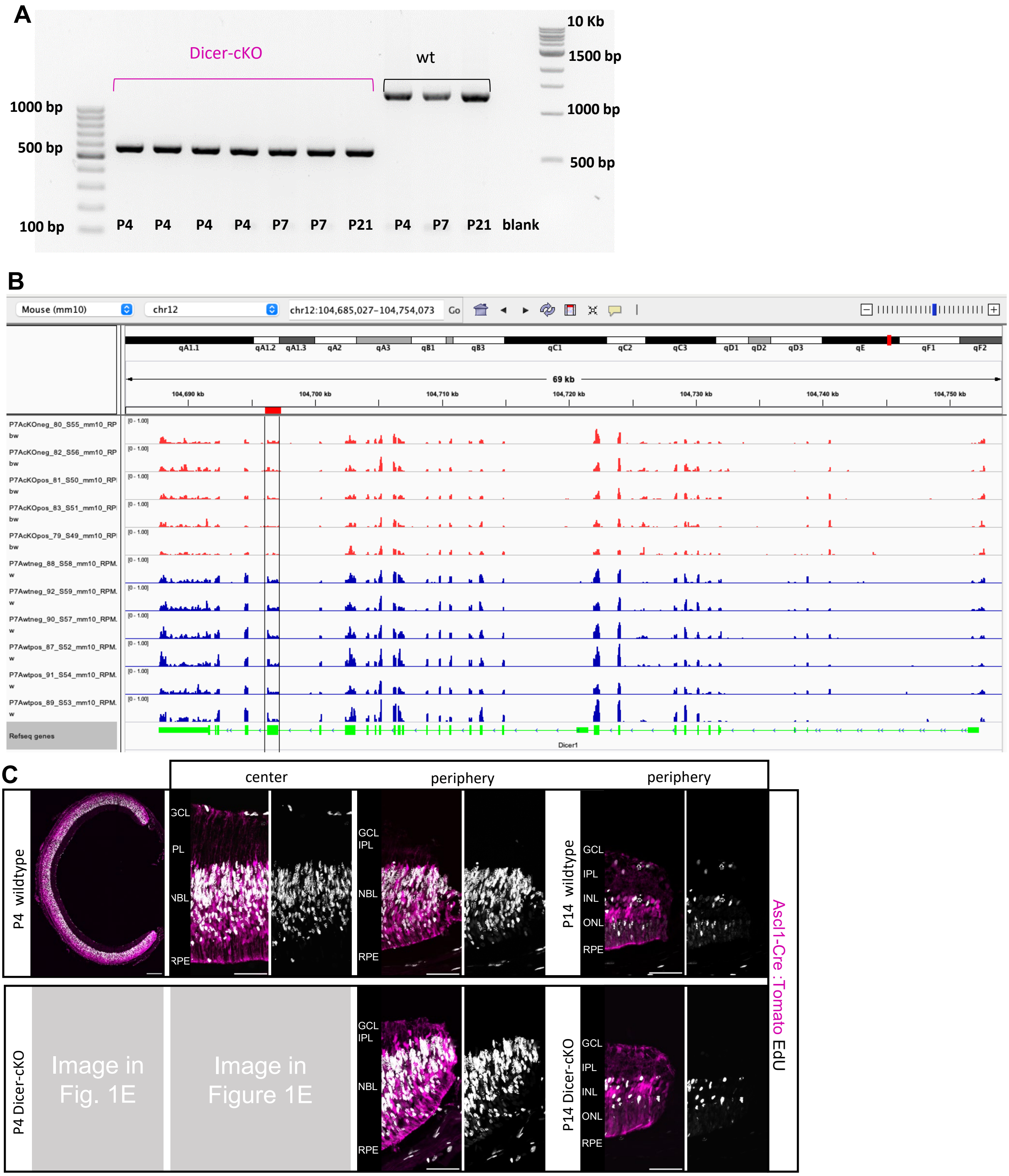

Figure S1. Dicer depletion in late proliferating retinal progenitor cells (related to Figure 1)**. A:** Gel image visualizing successful exon 23 deletion of *Dicer1* (~550 bp transcript) in P4, P7, and P21 retinal lysates of Ascl1-Cre:Dicer cKO mice (Dicer-cKO) mice as well as unaffected Dicer genes (1300 bp transcript) in P4, P7 and P21 wildtype (wt) retinal lysates. **B**: Screenshot of bulk RNA-seq data (BigWig files using Integrative Genomics Viewer IGV) showing exon 23 loss in P7 Dicer-cKO (AcKOpos) FACS-purified tomato+ cells as well as unaffected exons in wildtype (Awtpos) and reporter-negative cells (AcKOneg). **C:** P4 and P14 retinal cross sections with insets in higher magnification of central and peripheral areas, visualizing endogenous reporter expression of RPC progenies labeled at P1-3 as well as EdU-labeled cells at P3. Scale bar in while retinal image in C 200 µm, scale bars in higher magnification images: 50 µm. GCL: ganglion cell layer, IPL: inner plexiform layer, INL: inner nuclear layer, OPL: outer plexiform layer, ONL: outer nuclear layer, NBL: neuroblastic layer, RPE: retinal pigment epithelium.

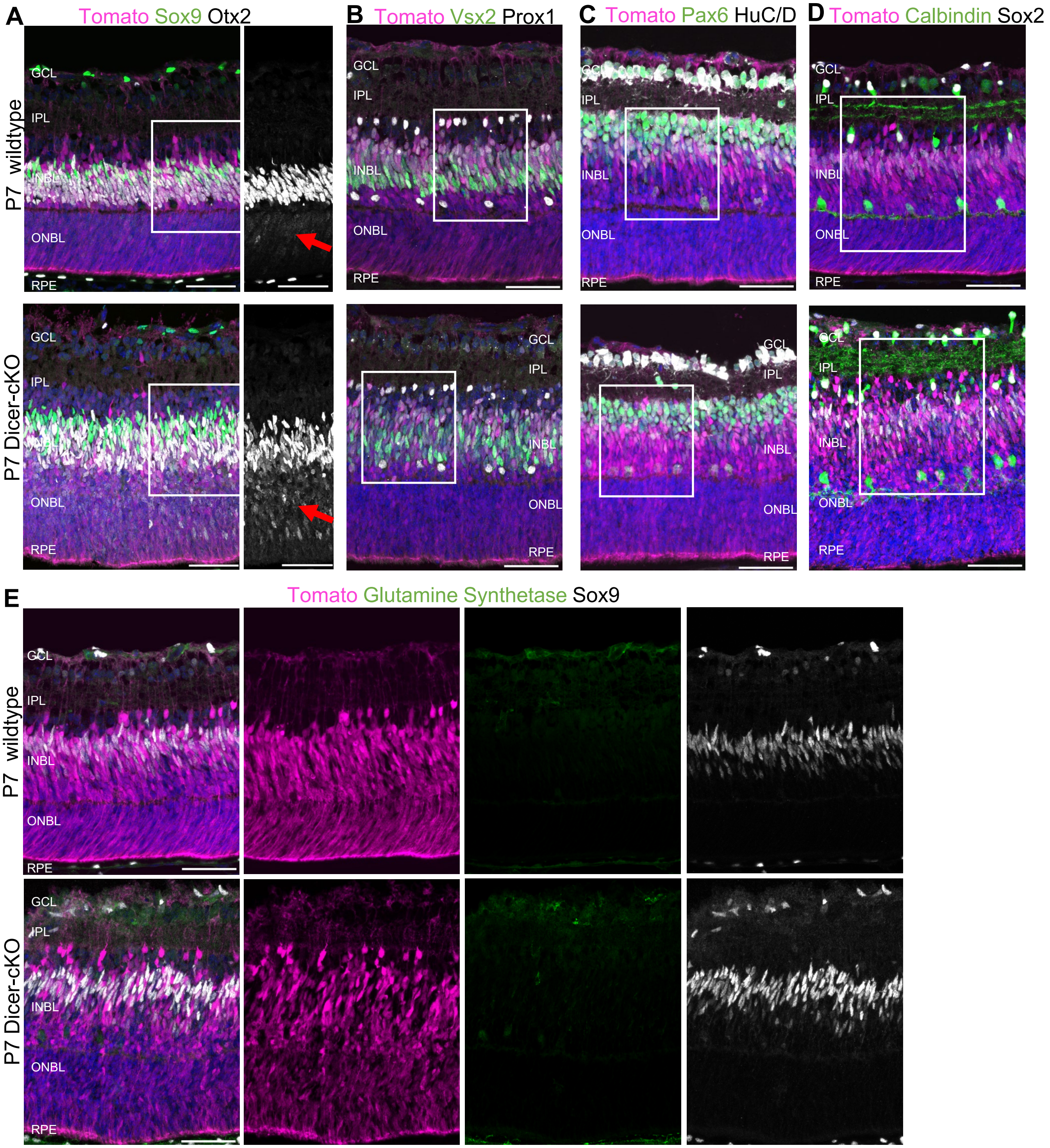

Figure S2. Dicer loss in late RPCs results in altered cellular structures of RPC progenies (related to Figure 1)**. A-E:** Immunofluorescent labeling with antibodies against Sox9, Otx2 (A), Vsx2, Prox1 (B), Pax6, HuC/D (C), Calbindin, Sox2 (D), and glutamine synthetase (GS), Sox9 (E) of P7 wildtype or Dicer-cKO central retinal cross sections to characterize Tomato+ RPC progenies in the INL. Insets in B-E are shown in the main Figure 2. Red arrows indicate Otx2+ cells in the ONBL. Scale bars in A-E: 50 µm. GCL: ganglion cell layer, IPL: inner plexiform layer, INBL: inner neuroblastic layer, ONBL: outer neuroblastic layer, RPE: retinal pigment epithelium.

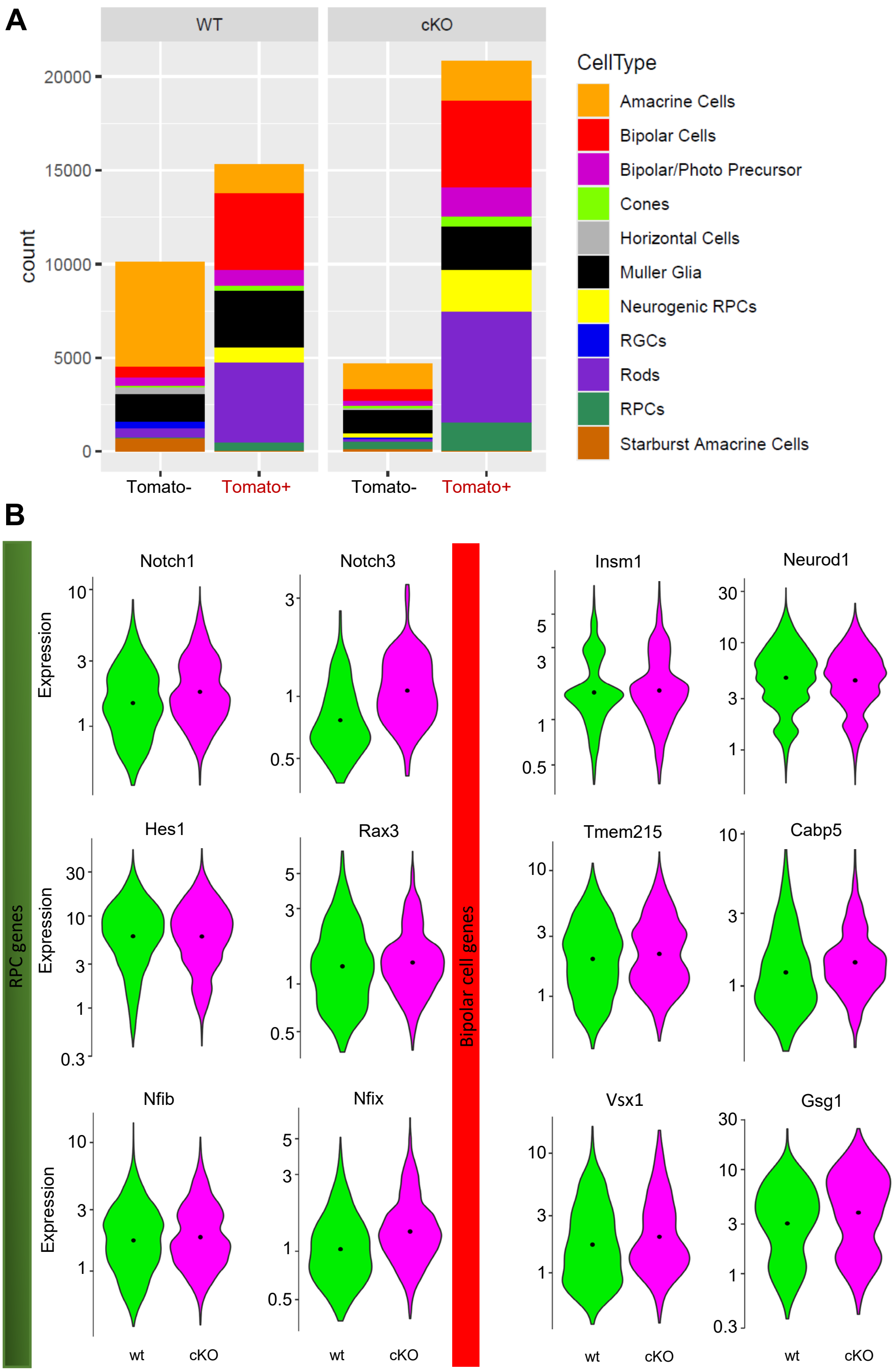

Figure S3. scRNA-Seq of P7 progenies reveals altered populations and gene expression (related to Figure 2). **A:** Numbers (counts) of scRNA-seq captured cells of FACS-purified P7 progenitor populations of Ascl-Cre:tdTomato wildtype and Dicer-cKO mice, as well as corresponding Tomato-negative fractions, colored by annotated cell type as determined by marker gene expression. **B:** Violin plots of the cellular expression of marker genes of RPCs, Müller glia, and bipolar cells facetted by genotype. RPCs: retinal progenitor cells, RGCs: retinal ganglion cells.

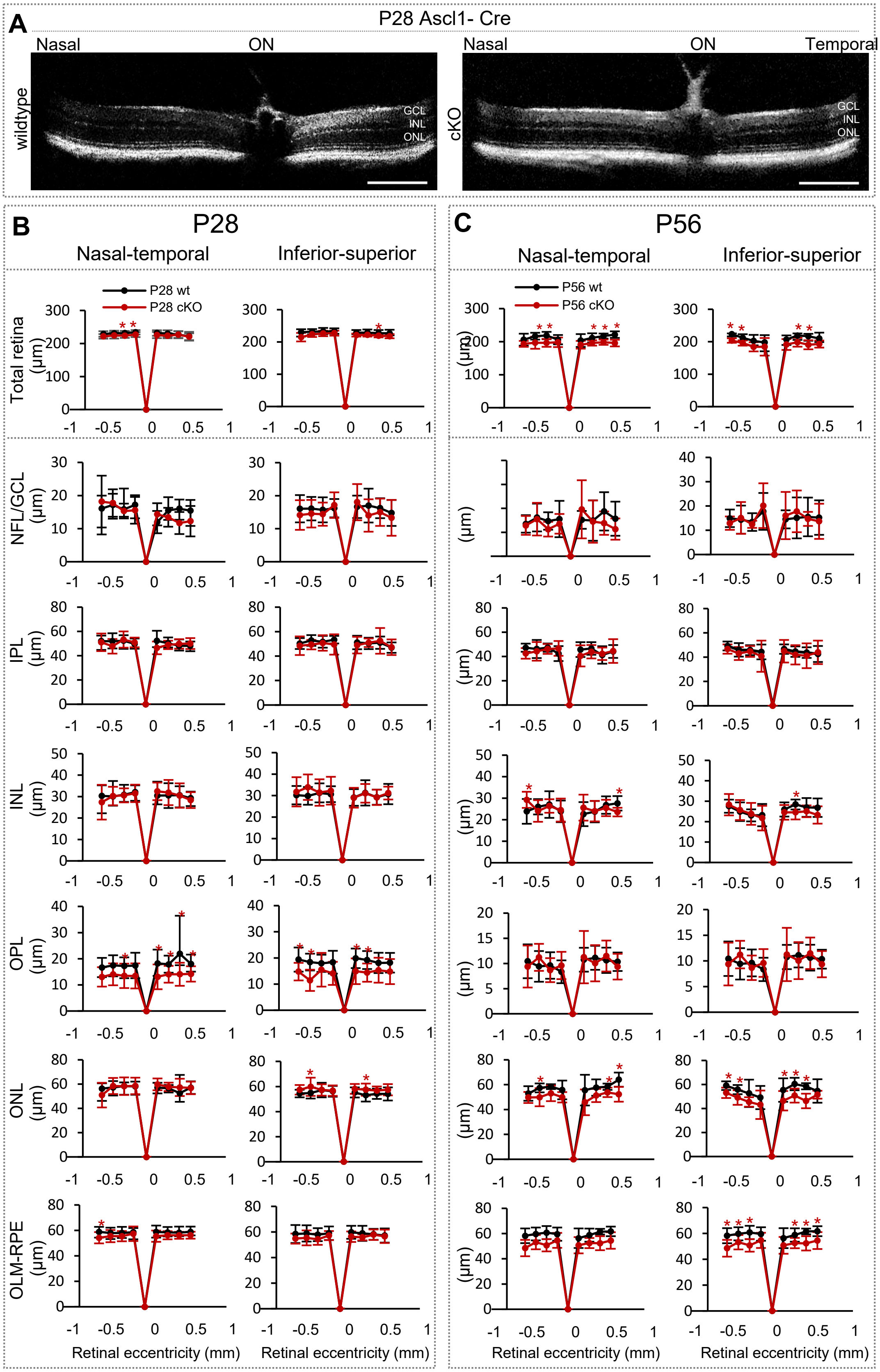

Figure S4: P28 and P56 Dicer-cKO retinas have normal appearance *in vivo* and minimal laminar alterations (related to Figure 4)**. A:** Spectral-domain optical coherence tomography (SD-OCT) images of postnatal day P28 wildtype (wt) and Ascl1-Cre:Dicer cKO$_{RPC}$ retinas (cKO), scale bars: 200 µm. **B-C:** Spider plots of the thickness (µm) of the total retina and individual layers measured at the nasal-temporal or inferior-superior axis, of P28 (B) wt (n=13) and P28 cKO (n=10) mice and (C) p56 wt (n=8) and P56 cKO (n=9) mice. Mean ± S.D. Significant differences are indicated, Mann-Whitney-U-test: *: p ≤ 0.05. NFL: nerve fiber layer, GCL: ganglion cell layer, IPL: inner plexiform, INL: inner nuclear layer, OPL: outer plexiform layer, ONL: outer nuclear layer, OLM: outer limiting membrane, RPE: retina pigment epithelium, ON: optic nerve.

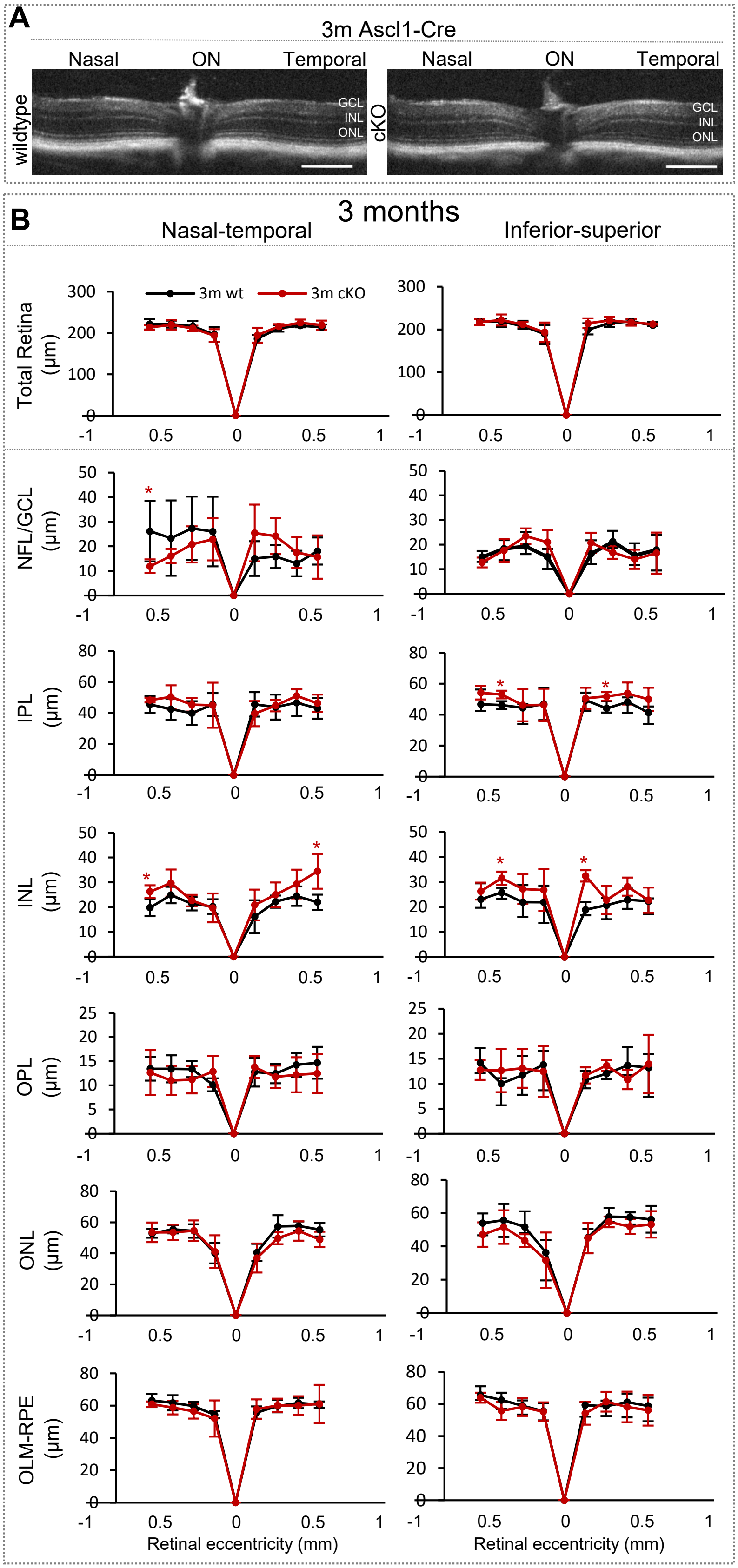

Figure S5: Three-month Dicer-cKO retinas show minimal laminar alterations (related to Figure 4)**. A:** Spectral-domain optical coherence tomography (SD-OCT) images of 3-month-old wildtype (wt) and Ascl1-Cre:Dicer cKO$_{RPC}$ mouse (cKO) retinas, scale bars 200 µm. **B:** Spider plots of the thickness (µm) of the total retina and individual layers measured at the nasal-temporal or superior-inferior axis, of 3-month old wt (n=8) and 3 month cKO (n=4) mice. mean ± S.D. Significant differences are indicated, Mann-Whitney-U-test: *: p ≤ 0.05. NFL: nerve fiber layer, GCL: ganglion cell layer, IPL: inner plexiform, INL: inner nuclear layer, OPL: outer plexiform layer, ONL: outer nuclear layer, OLM: outer limiting membrane, RPE: retina pigment epithelium, ON: optic nerve.

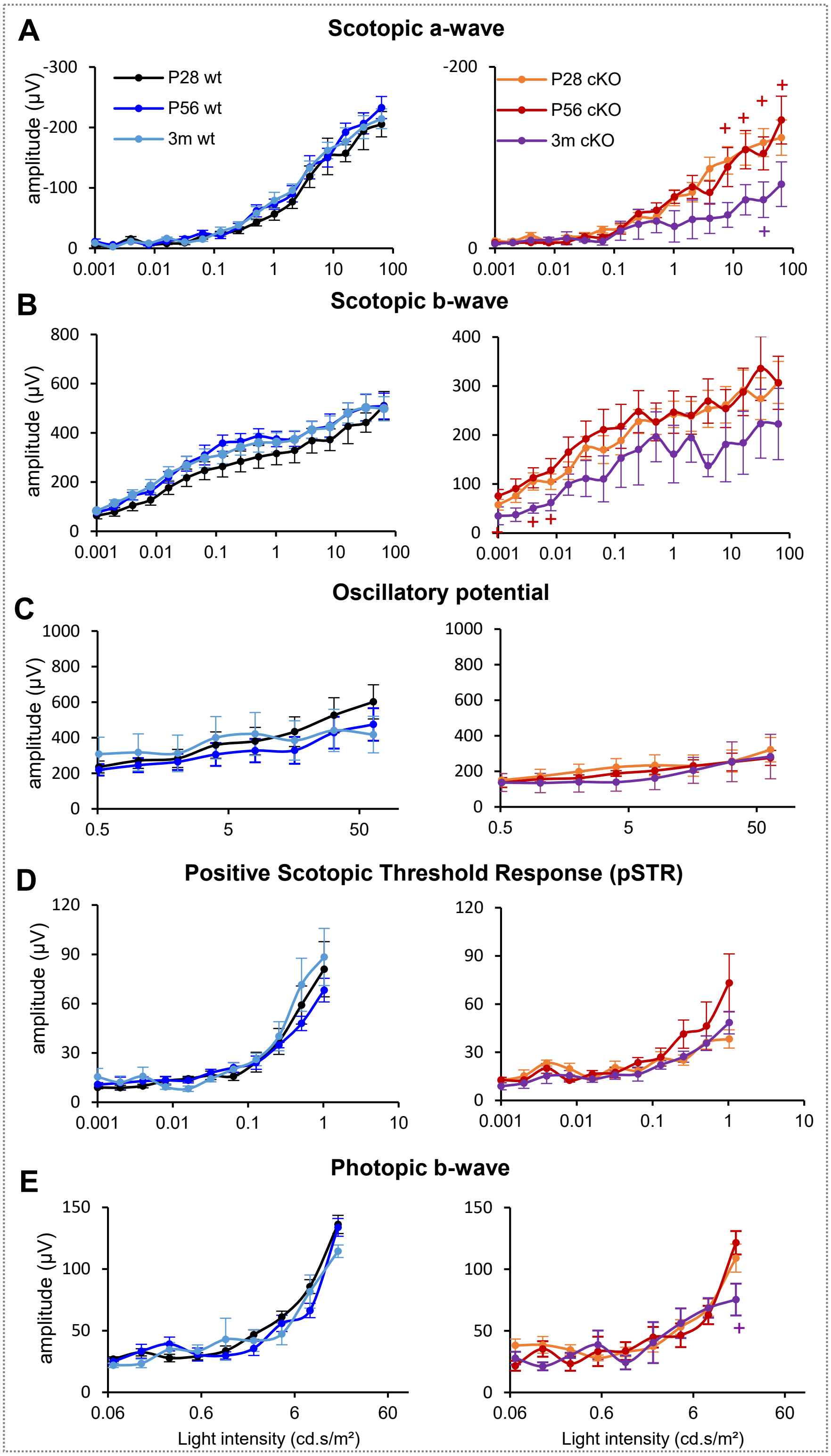

Figure S6: miRNA loss in late RPCs leads to impairments of rod function but only minimal alteration in cone function (related to Figures 4-8)**. A-B:** a-wave (A) and b-wave (B) of full-field scotopic electroretinogram recordings of postnatal day P28, P56, and 3-month-old wildtype (wt: P28: n=9, P56: n=11, 3m: n=5) and Ascl1-Cre:Dicer-cKO$_{RPC}$ mice (cKO, P28: n=10, P56: n=7, 3m: n=5). **C:** Full-field scotopic electroretinogram recordings showing oscillatory potential amplitudes as selected wave forms and intensity-dependent graphs for wildtypes (P28: n=8, P56: n=6, 3m: n=4) and Dicer-cKO mice (P28: n=5, P56: n=6, 3m: n=4). **D:** Positive scotopic threshold response (pSTR) of wildtypes (P28: n=9, P56: n=10, 3m: n=5) and Dicer-cKO mice (P28: n=10, P56: n=6, 3m: n=5). **E:** Full-field photopic electroretinogram recordings showing b-wave amplitudes as selected wave forms and intensity-dependent graphs of wildtypes (wt, P28: n=10, P56: n=11, 3m: n=5) and Dicer-cKO mice (P28: n=10, P56: n=7, 3m: n=5). All values are given as mean ± SEM. Mann-Whitney-U-test: wt vs. wt or cKO vs. cKO: +: $p \leq 0.05$.

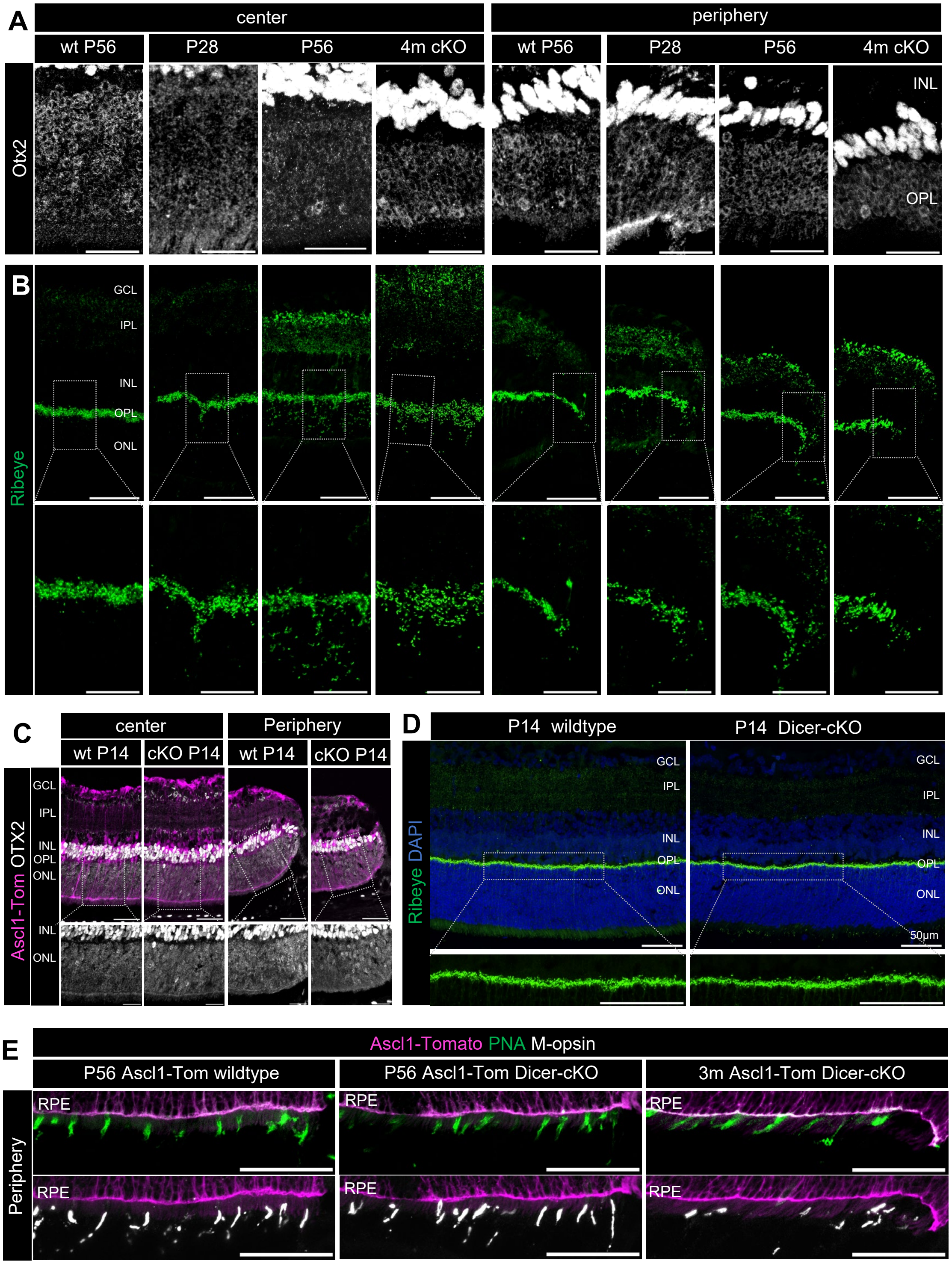

Figure S7: miRNA loss in late RPCs leads to reduced rod function and subsequent rod degeneration (related to Figures 4 and 5)**. A:** Immunofluorescent labeling using antibodies against Otx2 to label photoreceptor nuclei in the ONL in central or peripheral wildtype (wt) or Ascl1-Cre:Dicer cKO$_{RPC}$ (Dicer-cKO, cKO) retinas. **B:** Ribeye labeling to visualize ribbon synapse connections in central or peripheral retinas of P28 and P56 wildtype or Dicer-cKO retinas. **C:** Immunofluorescent labeling with antibodies against Otx2 in central or peripheral areas of P14 wildtype or Dicer-cKO retinas. **D:** Ribeye labeling in central or peripheral areas, as well as DAPI nuclear staining of P14 wildtype or Dicer-cKO retinas. **E:** Antibodies staining against peanut agglutinin (PNA) or to label entire cones or M-opsin to label L/M cones of peripheral P56 wildtype or Dicer–cKO retinas. Scale bars in A: 25 µm, in B, C: 50 µm, insets: 25 µm, in D, E: 50 µm. GCL: ganglion cell layer, IPL: inner plexiform layer, INL: inner nuclear layer, OPL: outer plexiform layer, ONL: outer nuclear layer, RPE: retinal pigment epithelium.

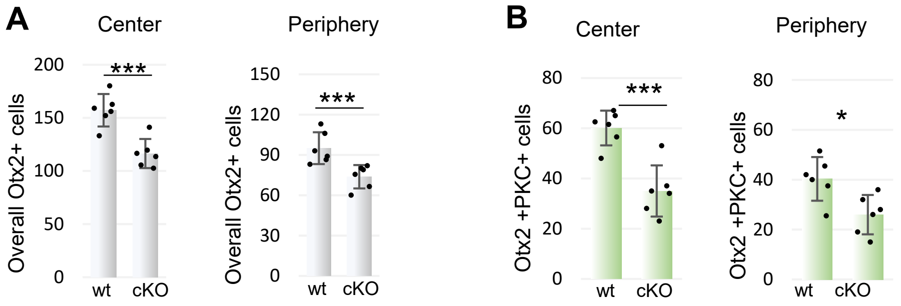

Figure S8. Bipolar cell numbers in P56 et and cKO retinas (related to Figure 6)**: A-B:** Absolute overall number of Otx2+ bipolar cells (BCs, A) and Otx2+ PKC+ rod BCs (B) in the central and peripheral retina of P56 wildtypes or Dicer-cKO mice; wt: n=6, cKO: n=6, mean ± S.D., Mann-Whitney-U-test: *: $p \leq 0.05$; **: $p \leq 0.01$; ***: $p \leq 0.005$. Cells per field.

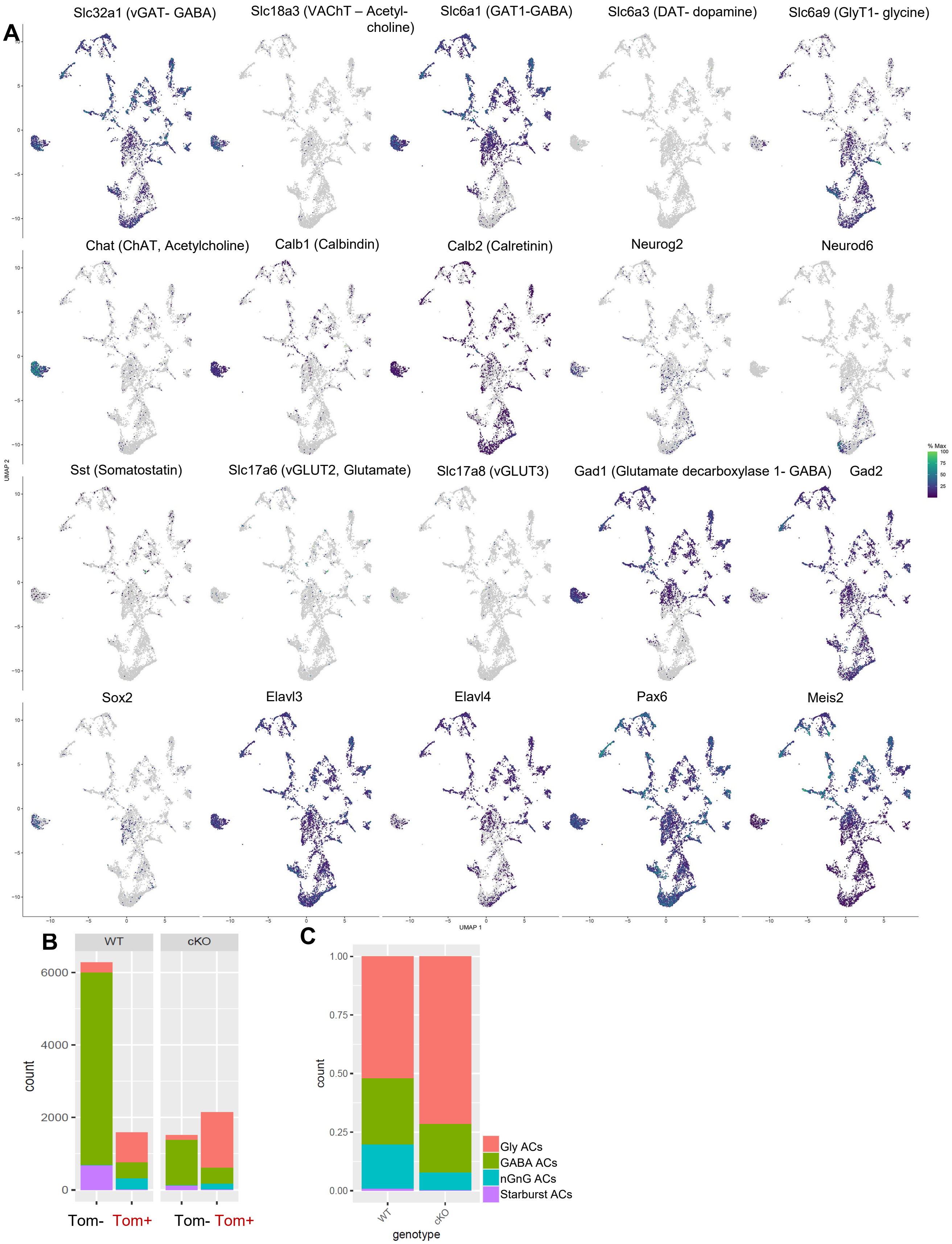

Figure S9. scRNA-Seq of P7 progenies shows altered amacrine cell populations (related to Figure 7). **A:** UMAP-dimension reduction of scRNA-seq FACS-purified integrated P7 Tomato+ wildtype (WT) and Dicer-cKO (cKO) amacrine cell (AC) progenies colored by annotated marker gene expression. **B:** AC type counts in scRNA-seq P7 wt and cKO AC Tomato+ progenies and Tomato- non-progenies, colored by annotated cell type as determined by marker gene expression. **C:** AC type proportions in scRNA-seq P7 wt and cKO AC progenies colored by annotated cell type as determined by marker gene expression.

3’UTR-seedless region of Elavl3

Output for StarMir — Prediction model training data (species): HITS-CLIP (mouse) — Target: Elavl3; miRNA: microRNA

Interactive site viewer for miRNA: all and target: Elavl3

3'UTR-seed | 3'UTR-seedless | CDS-seed | CDS-seedless | 5'UTR-seed | 5'UTR-seedless

miRNA	Site_Position	Hybrid Conformation	LogitProb†	Score	3'_bp*	ΔG_{hybrid}	ΔG_{nucl}*	ΔG_{total}*	Site_Access*	Upstream_Access (15nt)*	Dwstream_Access (15nt)*	Upstream_AU (30nt)*	Site_Location*
mmu-miR-25-3p	2025-2043	View	0.810	0.536	0	-21.300	0	-3.450	0.529	0.983	0.415	0.800	0.142
mmu-miR-25-3p	2025-2039	View	0.800	0.536	0	-15.800	0	-3.957	0.655	0.983	0.376	0.800	0.142
mmu-miR-25-3p	2289-2299	View	0.781	0.536	0	-20.200	-1.013	-7.103	0.400	0.788	0.240	0.700	0.217
mmu-miR-25-3p	2176-2188	View	0.770	0.536	0	-19.200	-2.154	-5.358	0.383	0.639	0.492	0.767	0.185
mmu-miR-25-3p	2376-2395	View	0.768	0.536	0	-17.600	0	-5.450	0.511	0.963	0.853	0.500	0.242
mmu-miR-25-3p	2289-2304	View	0.768	0.536	0	-22	-1.287	-6.087	0.367	0.788	0.200	0.700	0.217
mmu-miR-25-3p	2289-2306	View	0.689	0.536	0	-24.500	-1.287	-0.904	0.381	0.788	0.156	0.700	0.217
mmu-miR-25-3p	2533-2550	View	0.673	0.536	0	-22.700	-0.020	-9.655	0.330	0.507	0.646	0.533	0.287

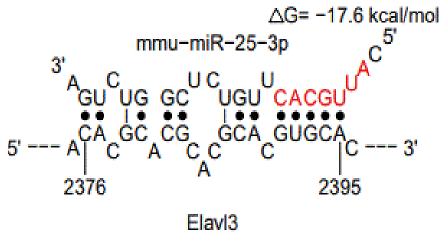

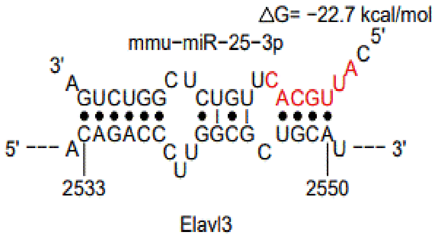

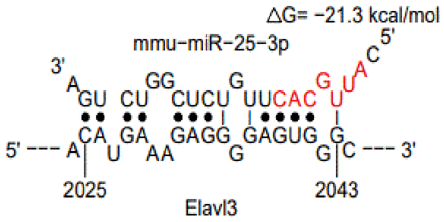

CDS-seed region of Elavl3

Output for StarMir — Prediction model training data (species): HITS-CLIP (mouse) — Target: Elavl3; miRNA: microRNA

Interactive site viewer for miRNA: all and target: Elavl3

3'UTR-seed | 3'UTR-seedless | CDS-seed | CDS-seedless | 5'UTR-seed | 5'UTR-seedless

miRNA	Site_Position	Hybrid Conformation	LogitProb†	Score	Seed_Position	Seed_Type*	3'_bp*	ΔG_{hybrid}	ΔG_{nucl}*	ΔG_{total}*	Site_Access*	Seed_Access*	Upstream_Access (15nt)*	Dwstream_Access (15nt)*	Upstream_AU (30nt)*	Dwstream_AU (30nt)*	Site_
mmu-miR-25-3p	1369-1399	View	0.551	0.536	1394-1399	offset-6mer	1	-27	-0.294	-12.139	0.441	0.507	0.484	0.365	0.400	0.467	0.855

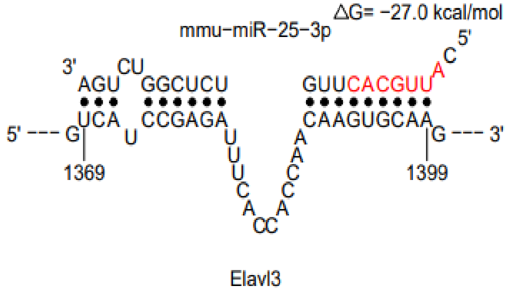

Figure S10. Additional predicted binding sites for miR-25 (related to Figure 9). Screenshot of StarMiR results (tables) and interaction schematics showing additional miR-25-3p binding sites in the 3’UTR and CDS in the *Elavl3* mRNA. miRNA seed sequences are indicated in red. Guide values for ΔG (hybrid stability): < -15 kcal/mol, probability: ≥ 0.5 indicate high hybrid stability/probability. Selected highlighted examples are shown as interaction schematics.

gene	P7 w1	P7 wt2	P7 wt3	P7 wt4	P7 cKO1	P7 cKO2	P7 cKO3	P7 cKO4
Aqp4	463	1123	821	1022	21	11	279	17
Ascl1	905	1243	1119	1134	1850	1371	1480	2161
Bak1	33	27	25	31	18	26	30	22
Bax	3322	1362	1025	1471	1459	1090	1217	1297
Cabp5	7363	8027	6363	5923	1003	2247	2549	1617
Calb1	260	707	532	570	50	598	615	552
Calb2	747	1830	2044	962	995	1820	2124	1204
Car2	65035	34155	27823	60612	29335	60450	53574	67692
Casp3	4455	9286	6090	6035	5604	7923	6312	6328
Casp6	1770	957	857	1307	1451	1789	1441	1696
Casp7	960	657	524	528	447	691	657	596
Casp8	124	156	213	154	111	355	226	335
Casp9	1935	1425	1605	1784	2423	1558	1527	1548
Casp9	1935	1425	1605	1784	2423	1558	1527	1548
Casz1	3608	1808	2007	2702	5183	2478	2415	2487
Ccnd1	1901	831	746	1577	4608	1705	1377	1772
Dkk3	62122	66867	52061	66636	72876	80585	69856	86045
Elavl3	568	539	416	524	940	795	1053	628
Elavl4	474	1691	1883	1325	645	1770	2136	1883
Glul	4866	3972	3273	4559	3974	4607	4153	4391
Gnat1	7988	982	2591	2727	1431	1370	1964	782
Gsg1	3536	1901	1337	1426	1296	779	854	944
Hes1	6897	3766	3186	6446	3775	6674	5497	12750
Hes5	4097	3078	2436	3571	2097	4577	3339	6878
Insm1	4341	3776	5683	5630	2149	5723	4603	3895
Meis2	4527	5633	6541	5873	6782	6486	5960	5822
Mki67	365	1286	2185	607	2918	4375	2138	2942
Neurod1	17289	33342	47585	60398	31479	57001	51259	64913
Neurog2	1447	2230	1437	2005	2131	1807	2285	4585
Nfia	2582	10110	7619	8745	3417	9313	9005	11303
Nfib	8202	24350	18898	17728	12847	21371	18302	20667
Nfic	3121	846	832	1580	3395	1223	1487	1491
Nfix	2178	1407	842	2142	2056	1815	2430	1882
Notch1	4613	2308	1477	3540	12170	3299	3726	4973
Notch3	226	179	127	254	369	211	206	225
Nr2e3	122293	32227	49395	53307	67437	50596	48604	40599
Nrl	41994	13677	28423	23027	20758	23018	22588	16834
Olig2	265	255	288	281	702	370	326	496
Otx2	49237	70426	63653	49407	64394	59837	47575	58020
Pax6	6914	12443	9566	12470	9859	16936	16905	19142
Pcna	4225	4488	5994	3858	3990	6432	5823	5565
Pcp2	1785	574	207	1177	109	93	186	118
Pde6b	79832	27069	50624	42745	43809	40666	43775	25862
Prkca	6784	11944	8331	10395	2627	4534	5128	5316
Rax	9037	1959	1509	4305	8091	2714	3536	4361
Rcvrn	13063	1993	3483	3868	1914	2473	3773	1939
Rho	210769	37802	85932	91998	63431	68416	90446	49855
Rlbp1	60708	30255	24088	39149	37776	42692	37809	44712
Slc1a3	44546	80362	63417	75463	34539	96305	79555	100466
Snap25	62349	93462	117633	95424	35215	93656	84557	67788
Sox2	1264	1812	1313	2734	1260	2957	2123	4242
Sox9	6186	5875	4630	7677	9887	7070	8033	12869
Sun1	7488	3580	4068	5399	10112	4527	5474	5085
Syne1	130	241	137	238	121	155	149	145
Tgs1	2217	7109	7328	4371	2806	5683	5088	5653
Tmem215	13993	25853	17930	18902	5208	9888	10337	8836
Trpm1	6924	4607	2201	3453	618	932	1554	1080
Vsx1	4454	10353	7915	6397	1616	3777	3754	3025
Vsx2	102155	75834	59592	72119	54051	42040	43055	44939

Table S1: Expression (normalized counts) of 60 selected genes (alphabetical order) in P7 wildtype (wt) and Dicer-cKO from bulk RNA-seq. Data related to Figures 2 and 9.

gene/miR	Accession number	wt	wt2	wt3	cko1	cKO2	cKO3	% reduction
Mirlet7b	MIMAT0000522	140279	115562	117639	54744	89637	67492	-43
Mir183	MIMAT0000212	58101	80167	61463	10395	30107	16059	-72
Mirlet7g	MIMAT0000121	34245	28671	25254	12371	31046	14956	-34
Mirlet7i	MIMAT0000122	18260	15204	14813	6818	12077	7622	-45
Mir342	MIMAT0000590	15699	14074	10122	6207	13386	9176	-28
Mir204	MIMAT0000237	15898	11639	11801	3210	13630	10734	-30
Mir182	MIMAT0000211	7100	9398	6927	2041	9029	3638	-37
Mir423	MIMAT0003454	5293	5817	5007	3412	5077	4633	-19
Mir130b	MIMAT0000387	5453	5102	4643	3795	4856	3300	-21
Mir328	MIMAT0000565	5206	5173	4809	4987	6823	5365	13
Mir23b	MIMAT0000125	4779	4611	3805	1840	4092	3094	-32
Mir99b	MIMAT0000132	3913	4101	3233	1294	3180	2218	-40
Mir98	MIMAT0000545	4273	3363	2821	704	2289	956	-62
Mir674	MIMAT0003740	3610	3202	2990	3405	3658	3024	3
Mir130a	MIMAT0000141	3520	3241	2954	5124	4031	2977	25
Mirlet7f	MIMAT0000525	4148	3037	2489	1046	3119	1424	-42
Mir210	MIMAT0000658	3560	2613	3055	2652	2989	1932	-18
Mirlet7c	MIMAT0000523	3289	2808	2355	1006	2062	1456	-46
Mir151	MIMAT0004536	2558	2624	1968	1504	2021	1760	-26
Mir30a	MIMAT0000128	2549	2138	1843	1540	2605	1365	-16
Mir744	MIMAT0004187	2172	2158	1931	962	1487	1488	-37
Mir92b	MIMAT0004899	1923	1827	1801	469	1430	1958	-31
Mirlet7a	MIMAT0000521	1998	1965	1502	601	1785	1010	-38
Mir15b	MIMAT0000124	1887	1853	1366	635	1794	1172	-29
Mir149	MIMAT0000159	1830	1531	1540	876	2098	1418	-10
Mir296	MIMAT0000374	1525	1449	1256	1312	1549	1239	-3
Mir484	MIMAT0003127	1415	1474	1106	351	1330	911	-35
Mir298	MIMAT0000376	1316	1373	1104	1354	983	686	-20
Mir181c	MIMAT0000674	1016	1484	1116	643	909	549	-42
Mir7a	MIMAT0000677	1267	1147	1119	720	1290	849	-19
Mir532	MIMAT0004781	1300	1202	1016	969	1314	1052	-5
Mir23a	MIMAT0000532	1231	1022	820	460	942	737	-30
Mir26b	MIMAT0000534	1171	1051	773	176	1064	512	-42
Mir382	MIMAT0000747	1052	1080	701	564	843	486	-33
Mir672	MIMAT0003735	1012	845	786	247	766	447	-45
Mir106b	MIMAT0000386	888	883	727	584	716	485	-29
Mir184	MIMAT0000213	800	870	708	247	861	906	-15
Mir96	MIMAT0000541	687	891	741	347	823	352	-34
Mir140	MIMAT0000151	658	665	612	638	855	589	8
Mir211	MIMAT0000668	994	322	504	573	558	223	-26
Mir335	MIMAT0000766	732	480	599	125	1001	482	-11
Mir384	MIMAT0004745	759	483	521	133	651	269	-40
Mir361	MIMAT0000704	524	648	437	469	488	382	-17
Mir34a	MIMAT0000542	474	623	506	174	306	271	-53
Mir340	MIMAT0004651	602	452	477	123	464	209	-48
Mir326	MIMAT0000559	502	447	422	499	566	276	-2
Mir30b	MIMAT0000130	447	442	405	149	598	299	-19
Mir323	MIMAT0000551	466	472	321	136	448	181	-39
Mir351	MIMAT0000609	431	396	381	147	441	387	-19
Mir676	MIMAT0003782	404	435	354	153	326	281	-36

Table S2: Top 50 highly expressed P7 wildtype (wt) miRNAs and their expression levels in the P7 Dicer-cKO retina (highest to lowest wt expression, normalized counts, bulk RNA-seq), Data related to Figure 9.

	GO biological process complete	Mus musculus - REFLIST (21836)	171	expected	over/ under	fold Enrichment	raw P-value	FDR
1	cell-cell adhesion involved in synapse maturation (GO:0090125)	2	2	0.02	+	> 100	6.10E-05	3.99E-03
2	positive regulation of neuromuscular synaptic transmission (GO:1900075)	3	2	0.02	+	85.13	1.82E-04	9.28E-03
3	regulation of neuromuscular synaptic transmission (GO:1900073)	3	2	0.02	+	85.13	1.82E-04	9.25E-03
4	nucleokinesis involved in cell motility in cerebral cortex radial glia guided migration (GO:0021817)	3	2	0.02	+	85.13	1.82E-04	9.22E-03
5	ascending aorta morphogenesis (GO:0035910)	4	2	0.03	+	63.85	3.62E-04	1.58E-02
6	nuclear migration along microtubule (GO:0030473)	4	2	0.03	+	63.85	3.62E-04	1.58E-02
7	modulation of microtubule cytoskeleton involved in cerebral cortex radial glia guided migration (GO:0021815)	4	2	0.03	+	63.85	3.62E-04	1.57E-02
8	negative regulation of amacrine cell differentiation (GO:1902870)	4	2	0.03	+	63.85	3.62E-04	1.57E-02
9	retrograde trans-synaptic signaling by trans-synaptic protein complex (GO:0098942)	5	2	0.04	+	51.08	6.00E-04	2.27E-02
10	ascending aorta development (GO:0035905)	5	2	0.04	+	51.08	6.00E-04	2.26E-02
11	positive regulation of basement membrane assembly involved in embryonic body morphogenesis (GO:1904261)	5	2	0.04	+	51.08	6.00E-04	2.25E-02
12	regulation of basement membrane assembly involved in embryonic body morphogenesis (GO:1904259)	5	2	0.04	+	51.08	6.00E-04	2.25E-02
13	metanephric nephron tubule morphogenesis (GO:0072282)	8	3	0.06	+	47.89	2.57E-05	1.92E-03
14	primitive erythrocyte differentiation (GO:0060319)	6	2	0.05	+	42.57	8.96E-04	3.12E-02
15	vascular endothelial growth factor receptor-2 signaling pathway (GO:0036324)	6	2	0.05	+	42.57	8.96E-04	3.11E-02
16	NMDA glutamate receptor clustering (GO:0097114)	6	2	0.05	+	42.57	8.96E-04	3.10E-02
17	cell motility involved in cerebral cortex radial glia guided migration (GO:0021814)	6	2	0.05	+	42.57	8.96E-04	3.10E-02
18	positive regulation of peptidyl-lysine acetylation (GO:2000758)	7	2	0.05	+	36.48	1.25E-03	4.00E-02
19	contact inhibition (GO:0060242)	7	2	0.05	+	36.48	1.25E-03	4.00E-02
20	negative regulation of hepatocyte proliferation (GO:2000346)	7	2	0.05	+	36.48	1.25E-03	3.99E-02
21	pointed-end actin filament capping (GO:0051694)	7	2	0.05	+	36.48	1.25E-03	3.98E-02
22	positive regulation of extracellular matrix assembly (GO:1901203)	14	4	0.11	+	36.48	3.42E-06	3.41E-04
23	angiogenesis involved in coronary vascular morphogenesis (GO:0060978)	7	2	0.05	+	36.48	1.25E-03	3.97E-02
24	negative regulation of photoreceptor cell differentiation (GO:0046533)	7	2	0.05	+	36.48	1.25E-03	3.96E-02
25	postsynaptic density protein 95 clustering (GO:0097119)	7	2	0.05	+	36.48	1.25E-03	3.95E-02
26	common bile duct development (GO:0061009)	7	2	0.05	+	36.48	1.25E-03	3.95E-02
27	metanephric tubule morphogenesis (GO:0072173)	11	3	0.09	+	34.83	7.43E-05	4.74E-03
28	commissural neuron axon guidance (GO:0071679)	15	4	0.12	+	34.05	4.63E-06	4.42E-04
29	detection of cell density (GO:0060245)	8	2	0.06	+	31.92	1.66E-03	4.91E-02
30	negative regulation of hepatocyte apoptotic process (GO:1903944)	8	2	0.06	+	31.92	1.66E-03	4.90E-02
31	male sex determination (GO:0030238)	16	4	0.13	+	31.92	6.14E-06	5.67E-04
32	regulation of photoreceptor cell differentiation (GO:0046532)	8	2	0.06	+	31.92	1.66E-03	4.89E-02
33	regulation of amacrine cell differentiation (GO:1902869)	8	2	0.06	+	31.92	1.66E-03	4.88E-02
34	positive regulation of vascular endothelial growth factor receptor signaling pathway (GO:0030949)	14	3	0.11	+	27.36	1.61E-04	8.59E-03
35	metanephric nephron morphogenesis (GO:0072273)	19	4	0.15	+	26.88	1.28E-05	1.08E-03
36	regulation of extracellular matrix assembly (GO:1901201)	20	4	0.16	+	25.54	1.60E-05	1.29E-03
37	angiogenesis involved in wound healing (GO:0060055)	16	3	0.13	+	23.94	2.45E-04	1.18E-02
38	endothelial cell morphogenesis (GO:0001886)	17	3	0.13	+	22.53	2.96E-04	1.37E-02
39	peptidyl-tyrosine autophosphorylation (GO:0038083)	17	3	0.13	+	22.53	2.96E-04	1.36E-02
40	ventricular trabecula myocardium morphogenesis (GO:0003222)	17	3	0.13	+	22.53	2.96E-04	1.36E-02
41	positive regulation of branching involved in ureteric bud morphogenesis (GO:0090190)	23	4	0.18	+	22.21	2.86E-05	2.06E-03
42	metanephric nephron tubule development (GO:0072234)	18	3	0.14	+	21.28	3.53E-04	1.56E-02
43	microtubule anchoring (GO:0034453)	24	4	0.19	+	21.28	3.41E-05	2.38E-03
44	regulation of branching involved in ureteric bud morphogenesis (GO:0090189)	25	4	0.2	+	20.43	4.04E-05	2.76E-03
45	metanephros morphogenesis (GO:0003338)	26	4	0.2	+	19.65	4.74E-05	3.19E-03
46	nuclear migration (GO:0007097)	33	5	0.26	+	19.35	5.52E-06	5.19E-04
47	pharyngeal system development (GO:0060037)	34	5	0.27	+	18.78	6.43E-06	5.87E-04
48	metanephric nephron epithelium development (GO:0072243)	21	3	0.16	+	18.24	5.66E-04	2.16E-02
49	positive regulation of mesenchymal cell proliferation (GO:0002053)	35	5	0.27	+	18.24	7.45E-06	6.73E-04
50	sex determination (GO:0007530)	28	4	0.22	+	18.24	6.42E-05	4.16E-03

Table S3: Gene Ontology (GO) analysis showing the top 50 biological processes of genes identified as P7 miRNA targets and to be upregulated in the P7 cKO. Data related to Figure 9.

	miR ID	Accession number	Nanostring P2 RPC counts	RNA-Seq bulk P7 RPC count
**1**	**mmu-miR-9**	**MIMAT0000142**	**63981**	**83**
**2**	**mmu-miR-16**	**MIMAT0000527**	**35845**	**40**
3	mmu-miR-204	MIMAT0000237	25967	9191
**4**	**mmu-miR-181a**	**MIMAT0000210**	**25080**	**100**
**5**	**mmu-miR-20a**	**MIMAT0000529**	**21395**	**0**
**6**	**mmu-miR-25**	**MIMAT0000652**	**20511**	**0**
7	mmu-miR-15b	MIMAT0000124	20032	1200
8	mmu-let-7g	MIMAT0000121	15750	19458
**9**	**mmu-let-7d**	**MIMAT0000383**	**15516**	**0**
10	mmu-miR-96	MIMAT0000541	12811	507
11	mmu-let-7i	MIMAT0000122	12334	8839
**12**	**mmu-miR-125a-5p**	**MIMAT0000135**	**9841**	**0**
13	mmu-let-7a	MIMAT0000521	9477	1132
**14**	**mmu-miR-15a**	**MIMAT0000526**	**8593**	**81**
**15**	**mmu-miR-1944**	**MIMAT0009409**	**8207**	**Not found**
16	mmu-miR-183	MIMAT0000212	7352	18854
17	mmu-let-7c	MIMAT0000523	7030	1508
18	mmu-miR-124	MIMAT0000134	7015	190
19	mmu-let-7b	MIMAT0000522	6889	70624
**20**	**mmu-miR-19a**	**MIMAT0000651**	**6501**	**0**
21	mmu-miR-125b-5p	MIMAT0000136	4547	80
22	mmu-miR-301a	MIMAT0000379	4339	Not found
23	mmu-miR-30c	MIMAT0000514	4087	40
24	mmu-let-7e	MIMAT0000524	4076	0
25	mmu-miR-135a	MIMAT0000147	3862	30
26	mmu-miR-130a	MIMAT0000141	3847	4044
27	mmu-let-7f	MIMAT0000525	3836	1863
28	mmu-miR-19b	MIMAT0000513	3405	7
29	mmu-miR-130b	MIMAT0000387	2976	3983
30	mmu-miR-1937a	MIMAT0009401	2592	Not found
31	mmu-miR-182	MIMAT0000211	2267	4903
32	mmu-miR-93	MIMAT0000540	2106	0
33	mmu-miR-342-3p	MIMAT0000590	1897	9589
34	mmu-miR-106b	MIMAT0000386	1689	595
35	mmu-miR-99b	MIMAT0000132	1669	2231
36	mmu-miR-103	MIMAT0000546	1479	20
37	mmu-miR-340-5p	MIMAT0004651	1380	265
38	mmu-miR-99a	MIMAT0000131	1375	185
39	mmu-miR-335-5p	MIMAT0000766	1352	536
40	mmu-miR-30d	MIMAT0000515	1330	0
41	mmu-miR-1937c	MIMAT0009429	1299	Not found
42	mmu-miR-350	MIMAT0000605	1212	261
43	mmu-miR-872	MIMAT0004934	1043	192
44	mmu-miR-210	MIMAT0000658	964	2525
45	mmu-miR-151-5p	MIMAT0004536	934	1762
46	mmu-miR-191	MIMAT0000221	917	0
47	mmu-miR-148a	MIMAT0000516	822	32
48	mmu-miR-30b	MIMAT0000130	770	349
49	mmu-miR-29c	MIMAT0000536	760	0
50	mmu-miR-30a	MIMAT0000128	691	1837

Table S4. 50 miRNAs highly expressed in P2 RPCs (Nanostring, *Wohl et. al., 2019*) and P7 RPCs/PCs (bulk RNA-Seq, averaged normalized counts). Top 10 miRNAs found only at P2 are highlighted in dark blue. *Expression levels *per se* cannot be compared as data were obtained from different techniques. Data related to Figure 9.

miRNA	Tools	Ensembl ID	Ensembl transcript	RefSeq ID	miRNA binding site in mRNA	binding region in mRNA	true target predicted probability	thermodynamic stability energy of duplex, < -15	miRNA seed binding
mmu-miR-20a-5p	miRWalk	ENSMUST00000003501.9	Canonical-current	NM_010487.2	660-676	CDS	1	-20.6	0
	STarMir	ENSMUST00000003501.9	Canonical-current	NM_010487.2	2389-2436	3'UTR	0.405	-15.7	1
	DT: TarBase	ENSMUST00000003501.9	n/a	n/a	n/a	n/a	n/a	n/a	n/a
	TargetScan	ENSMUST00000003501.7	Canonical - outdated	n/a	n/a	n/a	n/a	n/a	n/a
mmu-miR-15b-5p	miRWalk	ENSMUST00000003501.9	Canonical-current	NM_010487.2	2585-2609	3'UTR	0.846	-18.6	1
	STarMir	ENSMUST00000003501.9	Canonical-current	NM_010487.2	2586-2601	3'UTR	0.433	-23.1	0
	DT: TarBase	ENSMUST00000003501.9	n/a	n/a	n/a	n/a	n/a	n/a	n/a
	TargetScan	ENSMUST00000003501.7	Canonical - outdated	n/a	n/a	n/a	n/a	n/a	n/a
mmu-miR-25-3p	miRWalk	ENSMUST00000003501.9	Canonical-current	NM_010487.2	1755-1806	3'UTR	0.846	-21.2	1
	STarMir	ENSMUST00000003501.9	Canonical-current	NM_010487.2	1756-1774	3'UTR	0.411	-25.1	0
	STarMir	ENSMUST00000003501.9	Canonical-current	NM_010487.2	1369-1399	CDS	0.551	-27	0
	STarMir	ENSMUST00000003501.9	Canonical-current	NM_010487.2	2025-2043	3'UTR	0.810	-21.3	0
	STarMir	ENSMUST00000003501.9	Canonical-current	NM_010487.2	2376-2395	3'UTR	0.768	-17.6	0
	STarMir	ENSMUST00000003501.9	Canonical-current	NM_010487.2	2533-2550	3'UTR	0.673	-22.7	0
	DT: TarBase	ENSMUST00000003501.9	n/a	n/a	n/a	n/a	n/a	n/a	n/a
	TargetScan	ENSMUST00000003501.7	Canonical - outdated	n/a	n/a	n/a	n/a	n/a	n/a
mmu-miR-124-3p	miRWalk	ENSMUST00000003501.9	Canonical-current	NM_010487.2	3147-3164	3'UTR	0.923	-23.3	0
	STarMir	ENSMUST00000003501.9	Canonical-current	NM_010487.2	3148-3163	3'UTR	0.667	-28.5	0
	DT: TarBase	ENSMUST00000003501.9	n/a	n/a	n/a	n/a	n/a	n/a	n/a
	TargetScan	ENSMUST00000003501.7	Canonical - outdated	n/a	n/a	n/a	n/a	n/a	n/a

Table S5: List of predicted P2 RPC miRNAs, prediction tools used, and outcomes for *Elavl3* mRNA interaction. Guide values for ΔG (hybrid stability): < -15 kcal/mol, probability: ≥ 0.5 indicate high hybrid stability/probability. CDS: coding sequence, outdated Ensembl IDs are shown in red, matching sequences across different tools are shown in green. DT: Diana Tools. Data related to Figure 9.

Gene name	Forward sequence (5' to 3')	Reverse sequence (3' to 5')
Ascl1Cre wildtype	TCC AAC GAC TTG AAC TCT ATG G	CCA GGA CTC AAT ACG CAG GG
Ascl1Cre mutant	AAC TTT CCT CCG GGG CTC GTT TC	CGC CTG GCG ATC CCT GAA CAT G
tdTomato wildtype	AAG GGA GCT GCA GTG GAG TA	CCG AAA ATC TGT GGG AAG TC
tdTomato mutant	CTG TTC CTG TAC GGC ATG G	GGC ATT AAA GCA GCG TAT CC
Dicer	CCTGACAGTGACGGTCCAAAG	CATGACTCTTCAACTCAAACT
Dicer deletion	CCT GAC AGT GAC GGT CCA AAG	CCT GAG CAA GGC AAG TCA TTC
Elavl3 3'UTR	AATTCTAGTTGTTTAAACGAGCTCGG CATTTATTGCCCTCCCTCCC	CAGCTTGCATGCCTGCAGGTCGACT TTAAGATTCTGAACTTTTTATT TTCTGGCA TG

Table S6: Genotyping Primers and primers for *Elavl3 3' UTR* amplification, related to STAR Methods.

Antibody	Concentration	Company, Catalog #	Reference
**Primary antibodies/dyes**			
mouse anti Brn3a	1:500	Millipore, MAB1585	(Schneider et al., 2001)
mouse anti Calbindin	1:500	Millipore, ABN 2192	(Wassle et al., 1998)
goat anti ChAT	1:250	Millipore, AB144P	(West Greenlee et al., 1998)
mouse anti glutamine synthetase (GS)	1:500	Millipore, MAB302	(Grossman et al., 1994)
rabbit anti HuC/D	1:250	Fisher, A-21271	(Marusich et al., 1994)
rabbit anti Ki67	1:250	Abcam, ab15580	(Gerdes et al., 1984)
rabbit anti M-Opsin	1:300	Sigma-Millipore, AB5407	(Applebury et al., 2000)
goat anti Otx2	1:250 – 1:500	R&D Systems, AF1979	(Martinez-Morales et al., 2004)
rabbit anti Pax6	1:250	Invitrogen, 42-6600	(Marquardt et al., 2001)
Peanut agglutinin (PNA) fluorescein (a lectin)	1:300	Vector labs, FL-1071	(Blanks and Johnson, 1983)
rat anti Phosphohistone3 (PH3)	1:250	Novus, 102133-030	(Dhomen et al., 2006)
mouse anti PKC	1:500	Millipore, P5704	(Greferath et al., 1990)
guinea pig anti Prox1	1:250	SYSY antibodies, 509 005	(Dyer et al., 2003)
rat anti RFP (tdTomato)	1:1000	Antibodies online, ABIN334653	validation based on previous work (Wohl et al., 2019, Wohl et al., 2017)
rabbit anti Ribeye	1:500	SYSY antibodies, 192 103	(Schmitz et al., 2000)
goat anti Sox2	1:500	R&D Systems, AF2018	(Catena et al., 2004)
rabbit anti Sox9	1:250	Millipore, AB5535	(Poche et al., 2008)
sheep anti Vsx2	1:250	Fisher, 50-210-1941	(Burmeister et al., 1996)
rabbit anti ZO-1	1:300	Invitrogen, 61-730-0	(Konari et al., 1995)

Table S7: Primary antibodies, related to STAR Methods.

Antibody	Concentration	Company, Catalog #	Reference
**Secondary antibodies**			
Rhodamine Red 570 - AffiniPure $F(ab')_2$ Fragment Donkey Anti-Rat IgG (H+L)	1:1000	Jackson ImmunoResearch Laboratories, Inc. 712-296-150	validation based on previous work (Wohl et al., 2019, Wohl et al., 2017)
Alexa Fluor 488 - AffiniPure $F(ab')_2$ Fragment Donkey Anti-Goat IgG (H+L)	1:500	Jackson ImmunoResearch Laboratories, Inc 705-546-147	validation based on previous work (Wohl et al., 2019, Wohl et al., 2017)
Alexa Fluor 647 - AffiniPure $F(ab')_2$ Fragment Donkey Anti-Goat IgG (H+L)	1:500	Jackson ImmunoResearch Laboratories, Inc. 705-606-147	validation based on previous work (Wohl et al., 2019, Wohl et al., 2017)
Alexa Fluor 488- AffiniPure $F(ab')_2$ Fragment Donkey Anti-Mouse IgG (H+L)	1:500	Jackson ImmunoResearch Laboratories, Inc. 715-546-150	validation based on previous work (Wohl et al., 2019, Wohl et al., 2017)
Alexa Fluor 488 - AffiniPure $F(ab')_2$ Fragment Donkey Anti-Rabbit IgG (H+L)	1:500	Jackson ImmunoResearch Laboratories, Inc. 711-546-152	validation based on previous work (Wohl et al., 2019, Wohl et al., 2017)
Alexa Fluor 647 - AffiniPure $F(ab')_2$ Fragment Donkey Anti-Rabbit IgG (H+L)	1:500	Jackson ImmunoResearch Laboratories, Inc. 711-606-152	validation based on previous work (Wohl et al., 2019, Wohl et al., 2017)
Rhodamine Red 570 - AffiniPure $F(ab')_2$ Fragment Donkey Anti-Rat IgG (H+L)	1:1000	Jackson ImmunoResearch Laboratories, Inc. 712-296-150	validation based on previous work (Wohl et al., 2019, Wohl et al., 2017)

Table S8: Secondary antibodies, related to STAR Methods.

Name	Catalog number	microRNA ID
Negative control mimic	CN-001000-01-50	MIMAT0000039
mmu-miR-25-3p mimic	C-310564-05-0020	MIMAT0000652
mmu-miR-20a-5p mimic	C-310514-05-0002	MIMAT0000529
mmu-miR-15a-5p mimic	C-310510-05-0020	MIMAT0000526
mmu-miR-124-3p mimic	C-310390-05-0020	MIMAT0000134

Table S9: miRNA mimics, related to STAR Methods.
